# High‐Coordination and Nb‐Bridging of Bimetallic Amorphous P_6_‐Nb‐W‐P_5_ Clusters in Carbon Nanospheres for High‐Performance Sodium‐Ion Hybrid Capacitors

**DOI:** 10.1002/advs.202416942

**Published:** 2025-01-23

**Authors:** Shuxiao Hu, Baoquan Liu, Fanyan Zeng, Yang Pan, Dui Ma, Meilan Xie, Shenglian Luo

**Affiliations:** ^1^ School of Materials Science and Engineering Nanchang Hangkong University Nanchang Jiangxi 330063 P. R. China; ^2^ Key Laboratory of Jiangxi Province for Persistent Pollutants Control and Resources Recycle Nanchang Hangkong University Nanchang Jiangxi 330063 P. R. China; ^3^ College of Life Science Jiangxi Normal University Nanchang Jiangxi 330022 P. R. China

**Keywords:** amorphous P_6_‐Nb‐W‐P_5_ clusters, bimetallic atomic design, high‐coordination, Nb‐bridging, sodium ion hybrid capacitors

## Abstract

Amorphous clusters are gaining prominence as prospective hosts for sodium‐ion hybrid capacitors (SIHCs), but their efficacy is still affected by atomic coordination. Optimization of ion storage and charge transport can be achieved through high coordination and bimetallic configurations. Herein, high‐coordination amorphous P_6_‐Nb‐W‐P_5_ (Nb/W‐P) clusters are skillfully tailored by bridging Nb into the second shell of W in the W‐P_5_ configuration, nested in situ in conductive and stable N, P co‐doped carbon nanospheres (Nb/W‐P@NPC). Such clusters with high atom utilization can offer profuse Na^+^ storage sites due to their high coordination. As an electron donor, Nb‐bridging can subtly modify the electronic structure of clusters, and broaden the hybridization of *d‐p* orbitals, thus improving charge transfer efficiency and fostering diversified active sites. Compared with the low‐coordinated W‐P_L_@NPC and the high‐coordinated W‐P@NPC, the reversible capacity of Nb/W‐P@NPC upgrades to 556.3 mAh g^−1^ at 0.1 A g^−1^, alongside exceptional cycling stability at high rates. When integrated into SIHCs, the high energy density and high‐power output (223.6 and 9800 W kg^−1^) are achieved. By systematically exploring the effect of high coordination and bimetallic design on the storage efficacies of amorphous clusters, this study has greatly advanced the development of SIHC technologies.

## Graphical abstract

Amorphous clusters are gaining prominence as prospective hosts for sodium‐ion hybrid capacitors (SIHCs). The tailor P_6_‐Nb‐W‐P_5_ (Nb/W‐P) clusters are nested in carbon nanospheres (Nb/W‐P@NPC), where high coordination affords abundant storage sites, and Nb‐bridging broadens the hybridization of the *d‐p* orbitals, thus improving charge transfer efficiency and fostering diversified active sites. Nb/W‐P@NPC anode exhibits improved storage properties in SIHCs.

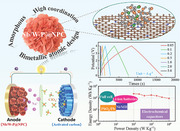

## 1 Introduction

Large‐scale energy storage systems are essential for grid stability and efficient energy utilization. Lithium/sodium‐ion batteries are well‐known for their stable voltage output, but the stringent requirements for charging and discharging platforms pose great challenges for the application of conversion‐type anodes.^[^ ^1^, ^2^ ^]^ Sodium‐ion hybrid capacitors (SIHCs) combine the high energy of battery‐type anodes with the high power of capacitor‐type cathodes, and their quasi‐triangular charge–discharge curves allow SIHCs to effectively utilize high‐energy‐density conversion‐type anodes.^[^ ^3^, ^4^ ^]^ Yet, the large radius of Na^+^ impedes its mobility within the anodes, leading to kinetic mismatches with the rapid adsorption/desorption at the cathodes. Thus, exploring anodes with wealthy storage sites and efficient charge transfer is imperative for advancing SIHCs.^[^ ^5^, ^6^ ^]^ Amorphous clusters featuring M‐N_x_ configurations offer high atomic utilization and isotropic charge transport channels, overcoming the restrictions of crystalline materials with fixed storage sites and transport paths. Such clusters in anode engineering can lower ion‐diffusion barriers and realize bulk‐phase‐indifferent fast ion storage.^[^ ^7^, ^8^ ^]^ However, the potential of amorphous clusters remains locked by their configurations, necessitating optimization of the coordination environments to enhance ion storage and migration.

Profound understanding of the M‐N_x_ configurations is essential for deciphering the correlation among structural features, electrochemical behaviors, and ion storage efficacies. Currently, the coordination number (*x*‐value) serves as the key parameter for governing the electronic distribution and *d*‐band energy levels around metal sites, which remarkably affects charge transfer and intermediate adsorption, thereby exerting profound effects on electrochemical properties.^[^ ^9^, ^10^ ^]^ Typical M‐N_4_ configurations (4 coordination sites) restrict the storage abilities due to their single electronic structure and binding sites.^[^ ^11^, ^12^ ^]^ Decreasing the coordination below 4, although low coordination can increase the bonding strength, electron concentration, and transfer efficiency,^[^ ^13^, ^14^ ^]^ it sacrifices the dynamic reconstruction of coordination bonds during conversion reactions and reduces the quantity of non‐metallic atoms available for bonding with Na^+^, thus ultimately curtailing the storage capacity. In contrast, high coordination beyond 4 can afford abundant storage sites by increasing the number of non‐metallic atoms and weakening the strength of coordination bonds, but the action mechanisms on Na^+^ storage performance of hybrid capacitors lack in‐depth exploration.

Atomic modulation strategies have become a paramount design principle for improving the energy storage performance of electrodes.^[^ ^15^, ^16^ ^]^ By precisely controlling the atomic arrangement and electronic structure of the M‐N_x_ coordinated amorphous clusters, and utilizing the differences in the electron affinity of metal atoms, fine‐tuning of electronic structure can be achieved, thereby reshaping the chemical potentials and reaction kinetics of electrodes.^[^ ^7^, ^17^ ^]^ Of note, the construction of adjacent bimetallic active sites can trigger electron delocalization for efficient electron transfer, and also induce *d*‐orbital hybridization to generate new energy levels, which affects the position and width of *d*‐band center and optimizes charge transfer efficiency.^[^ ^18^, ^19^ ^]^ Metal atoms such as Nb and W, with high chemical potentials, when coordinated with non‐metallic atoms, such as B, N, P, O, S, and Se, can reinforce the transfer efficiency and create additional sites for Na^+^ storage.^[^ ^20^, ^21^ ^]^ Particularly, P atoms, due to their high coordination ability with metal atoms and the existence of lone pairs that can easily interact with Na^+^, effectively augment the ion storage capabilities.^[^ ^22^, ^23^ ^]^ Such regulation strategies are of great importance for boosting the electrochemical properties of amorphous clusters, directly impacting the effective storage and efficient transfer of charges.

Herein, the coordination environment of W sites is meticulously tailored by controlled phosphatization, and the amorphous P_6_‐Nb‐W‐P_5_ (Nb/W‐P) clusters with high‐coordination bimetallic configuration are nested in situ in N, P co‐doped carbon nanospheres (Nb/W‐P@NPC). Systematic structural analyses and theoretical calculations reveal that the amorphous W‐P_5_ (W‐P) clusters exhibit high atom utilization and fast charge transport, and their enriched high‐coordination P also significantly increases the storage sites for Na^+^. In addition, by bridging high‐potential Nb in the second shell of W, the optimization of *d‐p* orbital energy levels and the broadening of their hybridization range are achieved, which effectively balances adsorption energy, enriches storage sites and further facilitates charge transfer. Benefiting from this design, the Nb/W‐P@NPC anode maintains a high reversible capacity of 316.7 mAh g^−1^ after 3000 cycles at 3.0 A g^−1^, with a capacity retention of nearly 100%. The high‐coordination bimetallic amorphous clusters effectively relieve the kinetic mismatch with the cathode, ensuring high energy density and high‐power output for SIHCs. These findings endorse the notion that the tuning of the coordination environments of amorphous clusters can dramatically improve the overall performance of Na^+^ storage in hybrid capacitors.

## 2 Results and Discussion

### 2.1 Structural Characterization

**Figure 1:**
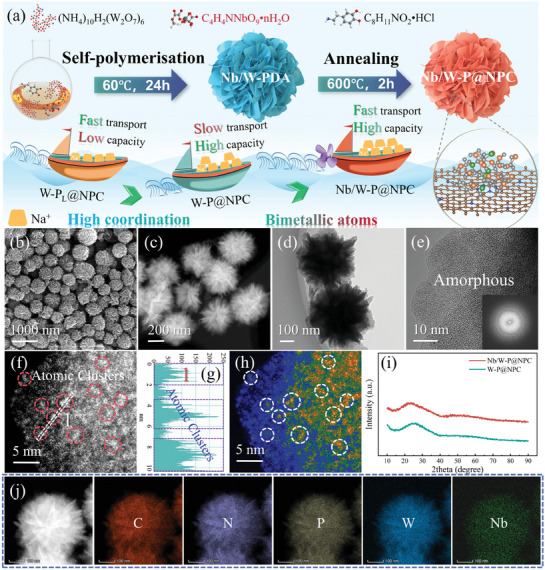
a) Brief description for the prepared process of Nb/W‐P@NPC and its structural characteristics (the amount of cargo symbolizes the capacity of the electrodes, and the traveling way reflects the difference in charge transfer ability). Morphological and microstructural features of Nb/W‐P@NPC: b) SEM image, c) HAADF‐STEM image, d) TEM image, e) HR‐TEM image with FFT image (inset), f,h) Aberration‐corrected HAADF‐STEM images, g) Inverse FFT‐derived atomic profile, i) XRD patterns of W‐P@NPC and Nb/W‐P@NPC, and j) Elemental mapping.

**Figure** **1**a delineates the synthesis process of Nb/W‐P@NPC and its structural features. Starting with the chelation of ammonium niobate oxalate (Nb source) and ammonium tungstate (W source) with dopamine (DA) in a water/ethanol solvent, Nb/W‐DA intermediates are generated. Subsequently, anisotropic growth under ammonia‐induced self‐polymerization converts these intermediates into Nb/W‐polydopamine (Nb/W‐PDA) precursors, as evidenced by X‐ray diffraction (XRD, Figure S1, Supporting Information). Scanning electron microscopy (SEM, Figure S2, Supporting Information) discloses a homogeneous 600 nm‐sized sheet‐sphere morphology. Notably, the morphology of the products evolves systematically with the mass ratio of Nb source (Figure S3, Supporting Information). The pure W source and Nb source yield distinct sheets and spheres, respectively, while the others forming intermediate morphologies. Especially at 20% Nb source, the even sheet‐sphere structure highlights the synergistic interplay between spherical growth tendency of Nb and W‐induced sheet formation. Upon phosphatization, Nb/W‐PDA is transformed into amorphous Nb/W‐P clusters nested in N, P co‐doped carbon nanospheres (NP‐C), forming Nb/W‐P@NPC composite. SEM (Figure 1b) and transmission electron microscopy (TEM, Figure 1c,d) present that the composite inherits the sheet‐sphere structure of the precursors. The absence of lattice fringes in the high‐resolution TEM (HR‐TEM) image and the existence of a diffuse spot in the fast Fourier transform (FFT, inset) image imply the amorphous nature of Nb/W‐P@NPC (Figure 1e). Particularly, aberration‐corrected high‐angle annular dark‐field scanning transmission electron microscopy (AC HAADF‐STEM, Figure 1f,h) images visualize the high dispersion of atomic clusters, revealing that the spontaneous aggregation of isolated bright spots (single Nb or W atoms) into amorphous clusters of ≈3 nm with many smaller or larger aggregates of clusters, as defined in the atomic profiles (region 1, Figure 1g). In contrast, W‐P@NPC (Figures S4 and S5, Supporting Information) exhibits loose nanosheets with rich amorphous clusters. XRD patterns (Figure 1i) further verify the amorphous structure, but the annealing (Figure S6, Supporting Information) does not trigger the Nb/W‐P@NPC crystallization into niobium or tungsten phosphides as comparisons. Raman spectra (Figure S7, Supporting Information) lack the vibration peaks in Nb/W‐P@NPC and W‐P@NPC, indicative of the weak coordination strength of the clusters. The *I*_D_/*I*_G_ ratios between the disordered *D* band (1342 cm^−1^) and graphitic *G* band (1589 cm^−1^) are close to 1, signifying the typical amorphous carbon.^[^ ^24^, ^25^ ^]^ XRD patterns and Raman spectra (Figure S8, Supporting Information) also disclose the amorphous nature of low‐coordination W‐P_L_@NPC and NP‐C. Furthermore, elemental mapping (Figure 1j and Figure S9, Supporting Information) and energy‐dispersive X‐ray spectroscopy (EDX, Figure S10, Supporting Information) attest the even distribution and types of elements in Nb/W‐P@NPC. Collectively, these findings exhibit a tailored strategy for the fabrication of Nb/W‐P@NPC with highly dispersed bimetallic amorphous clusters, highlighting its unique structural design.

**Figure 2:**
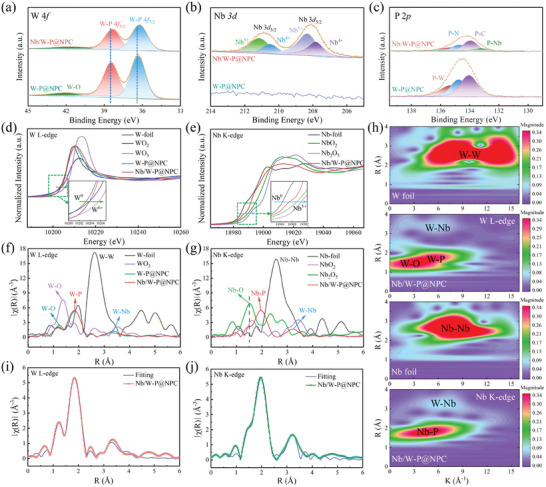
HR‐XPS spectra for a) W 4*f*, b) Nb 3*d*, and c) P 2*p* in W‐P@NPC and Nb/W‐P@NPC. d,e) Normalized XANES spectra, f,g) FT‐EXAFS curves, and h) WT‐EXAFS contours of W L‐edge and Nb K‐edge in W‐P@NPC, Nb/W‐P@NPC and reference samples, and FT‐EXAFS fittings in *R* space of i) W L‐edge and j) Nb K‐edge in Nb/W‐P@NPC.

Electron paramagnetic resonance (EPR) spectroscopy is an effective tool for identifying vacancy defects of materials. Comparative analyses (Figure S11, Supporting Information) show the intensified signals in W‐P@NPC, implying an abundance of unsaturated vacancies and edge‐exposed bonds in the high‐coordination W‐P clusters, thus offering additional storage sites.^[^ ^26^ ^]^ The diminished signals in Nb/W‐P@NPC can result from the Nb bridging with the edge‐unsaturated bonds of W‐P clusters, filling some vacancies. Regarding the porosity (Figure S12, Supporting Information), N_2_ adsorption‐desorption isotherms of the composites exhibit a type IV hysteresis loop characteristic of porous structures, facilitated by the sheet‐like surface morphology and featuring macropores. Post‐cluster etching, the specific surface area of NP‐C increases significantly, and the pores at 3.5 nm are consistent with the original cluster size. These pore features can facilitate electrolyte infiltration and accelerate reaction kinetics. As depicted by the thermogravimetric (TG) analyses in Figure S13 (Supporting Information), in addition to the initial moisture loss, thermal degradation beyond 400 °C indicates that the samples are conversed to WO_3_ or Nb_2_O_5_ alongside gas release. Combined with the result of inductively coupled plasma (ICP, atom ratio of 0.39:1 in Nb/W), the composition of Nb/W‐P@NPC is calculated to be 78.7% for Nb/W‐P and 21.3% for NP‐C, suggesting that the amorphous clusters in NP‐C is dense.^[^ ^27^, ^28^ ^]^ The appropriate carbon can effectively inhibit the agglomeration of clusters with large surface energy and high loading, and improve the conductivity and stability of the composite. X‐ray photoelectron spectroscopy (XPS) delve into the surface chemical details of samples. The full spectra and elemental contents (Figure S14 and Table S1, Supporting Information) imply a low P content in W‐P_L_@NPC and the removal of clusters in NP‐C. The HR W 4*f* spectra (**Figure** **2**a) display the characteristic peaks for W‐P bonding at 38.3 eV (4*f*_5/2_) and 36.2 eV (4*f*_7/2_), and the slight surface oxidation (W─O bonds) at 41.7 eV.^[^ ^29^, ^30^, ^31^ ^]^ Shifts of W 4*f* peaks in Nb/W‐P@NPC toward low binding energies signify that Nb‐bridging as an electron donor enhances the interaction with high‐coordination W‐P clusters and lowers the valence of W. The clear Nb 3*d* peaks in Figure 2b further imply the incorporation of Nb into the W‐P clusters.^[^ ^32^ ^]^ As for the P 2*p* spectra (Figure 2c and Figure S15, Supporting Information), NP‐C and NP‐C_PDA_ display the peaks of P─N and P─C bonding.^[^ ^33^, ^34^ ^]^ With the introduction of W‐P clusters and Nb bridging, the extra P‐W and P‐Nb peaks indicate the effective coordination of P with W and Nb. In addition, the C 1*s* spectra display the coexistence of C─C, C─N, and C─O bonds in all samples (Figure S16a, Supporting Information). For the N 1*s* spectra (Figure S16b, Supporting Information), the standard peaks for Graphite‐N, Pyrrolic‐N, and Pyridinic‐N appear in N–C at 400.7, 399.4, and 398.4 eV, respectively.^[^ ^35^ ^]^ The spectra of O 1*s* in W‐P@NPC and Nb/W‐P@NPC exhibit similar C–O and C = O characteristic peaks (Figure S17, Supporting Information), further implying the presence of slight oxidation or adsorbed oxygen on the electrode surface.^[^ ^36^ ^]^ In NP‐C, W‐P@NPC, and Nb/W‐P@NPC, these peaks are shifted toward high binding energies, accompanied by reduced intensities of Pyrrolic‐N and Pyridinic‐N after phosphatization, implying the efficacy of P in modulating the electronic structure and bonding environments of the clusters. These findings suggest that Nb/W‐P@NPC has efficient P‐W and P‐Nb coordination, as well as robust bonding between clusters and NP‐C, which together provide favorable conditions for enhanced Na^+^ storage.

X‐ray absorption spectroscopy (XAS) can reveal the coordination environments of amorphous clusters in composites. The X‐ray absorption near‐edge structure (XANES) results (Figure 2d,e) indicate that the average valences of W and Nb in Nb/W‐P@NPC are about +4 and +3, respectively, signifying their high electron‐donating ability. In Figure 2f,g and Figure S18 (Supporting Information), the extended X‐ray absorption fine structure (EXAFS) spectra show a distinct W‐P coordination peak at 1.82 Å for the W L‐edge and a Nb‐P feature at 1.95 Å for the Nb K‐edge, illustrating similar coordination environments of Nb and W with P in Nb/W‐P@NPC. The peaks of W─O and Nb─O bonds may derive from the slight surface oxidation of clusters. Intriguingly, the peaks for W‐Nb coordination at 3.34 Å in the W L‐edge and 3.22 Å in the Nb K‐edge imply the bridging between Nb and W in the second shell of W in Nb/W‐P@NPC, which facilitates an asymmetric distribution for efficient charge transfer. The coordination bond lengths can be identified using the wavelet transform (WT) of the EXAFS data (Figure 2h). The strong signals for W‐P (1.70 Å) and Nb‐P (1.84 Å) are evident in the W and Nb contour plots of Nb/W‐P@NPC. Relative to the reference WO_2_ and WO_3_ (Figure S19, Supporting Information), the signal at 1.46 Å may refer to the surface W─O bonds from slight oxidation (Figure 2h and Figure S20, Supporting Information). Correspondingly, the absence of W‐W signal (2.62 Å) and the existence of W‐Nb peak further validate the notion of W‐Nb bridging. Quantitative EXAFS fitting in both R and K spaces (Figure 2i,j, Figures S21 and S22, Supporting Information) can define the coordination environments of W and Nb. As listed in Table S2 (Supporting Information), in Nb/W‐P@NPC, the coordination number of W‐P is 4.5 and that of Nb‐P is 5.6, which implies that individual W coordinates with 5 P and each Nb bonds to 6 P, respectively, thus identifying the unique amorphous P_6_‐Nb‐W‐P_5_ configuration. The number of 4.5 for W‐P clusters in W‐P@NPC indicates that Nb bridging does not alter the initial W‐P coordination, contrasting with previously reported low‐coordination amorphous W‐P clusters (W‐P_L_@NPC, with each W coordinating only 2 P).^[^ ^29^ ^]^ These results indicate that the high‐coordination amorphous Nb/W‐P clusters are created by Nb bridging to the second shell of W in Nb/W‐P@NPC.

### 2.2 Theoretical Studies

**Figure 3:**
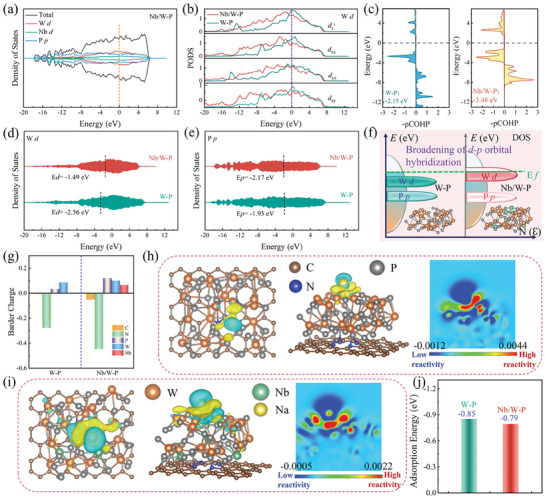
DFT analyses of amorphous W‐P and Nb/W‐P clusters: a) Calculated DOS for Nb/W‐P, b) *d* orbitals of W in different dimensions, c) pCOHP results, d) *d*‐band centers of W, and e) *p*‐band centers of P in the PDOS, f) Schematic diagram of *d*‐*p* orbital hybridization of W and P, and g) Bader charge analyses. Differential charge density distributions (different views) and cross‐sectional contours of Na^+^ adsorbed on the model surface: h) W‐P and i) Nb/W‐P, and j) corresponding adsorption energies.

Density Functional Theory (DFT) analyzes the structural characteristics of amorphous clusters in Na^+^ storage. Based on the fitted results of EXAFS, the optimized models of high‐coordination amorphous W‐P and Nb/W‐P clusters are shown in Figure S23 (Supporting Information). Near the Fermi energy level, the density of states (DOS, **Figures** **3**a and S24a, Supporting Information) indicate the high electron occupancy for both clusters, predicting efficient charge transfer. Analyses of projected DOS (PDOS, Figure 3b and Figure S24b, Supporting Information) reveal that the orbital charge densities of Nb/W‐P decrease slightly in all dimensions, which can be attributed to Nb bridging that tempers the orbital overlap between W and P, thus optimizing the electronic structure and adsorption energy of clusters. Based on the projected crystal orbital Hamilton population (pCOHP, Figure 3c) reflecting bonding strength, the bonding energy of the Nb/W‐P (−3.48 eV) is higher than that of the W‐P (−2.15 eV), indicating that Nb incorporation effectively enhances the bonding strength and reactivities of W‐P clusters for efficient Na^+^ storage.^[^ ^37^ ^]^ In Nb/W‐P, Nb bridging also induces *d‐p* orbital recalibration (Figure 3d,e), resulting in the upshift of the *d*‐band center of W (−2.56 to −1.49 eV) and the downshift of the *p*‐band center of P (−1.93 to −2.17 eV), which broadens the orbital hybridization range (Figure 3f). The bimetallic bridging accelerates charge transfer, balances Na^+^ adsorption, and generates diverse storage sites. Bader charge analyses (Figure 3g) highlight the function of Nb as an electron donor, where its high chemical potentials boost the charge density of P and facilitate charge transfer from Nb/W‐P to NP‐C. Differential charge density and cross‐sectional contours (Figure 3h,i) visually depict this phenomenon, with yellow denoting electron‐rich zones and cyan electron‐poor regions. In contrast to W‐P, Nb/W‐P exhibit distinct regions of charge depletion and accumulation upon Na^+^ adsorption, with large red zones denoting electron enrichment, signifying that Nb bridging can promote charge redistribution. The average energy of Na^+^ adsorption (Figure 3j) displays that the energy of Nb/W‐P (−0.79 eV) is slightly lower than that of W‐P (−0.85 eV), indicating that the bimetallic design can optimize charge transfer ability and adsorption behaviors of the clusters. These results suggest that Nb bridging can effectively modulate the electronic structure and architectural features of amorphous W‐P clusters, thereby improving the charge storage and transport efficiency of electrodes.

### 2.3 Storage Performance and Mechanism

**Figure 4:**
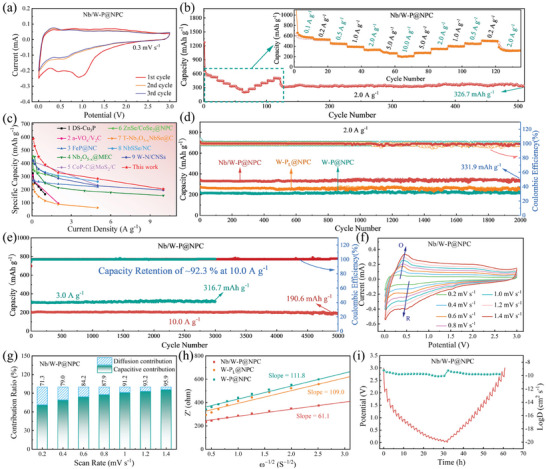
Properties of Nb/W‐P@NPC anode for SIHCs: a) Initial three CV curves at 0.3 mV s^−1^. b) Rate capability, c) Comparison of reversible capacity (0.1 A g^−1^) and rate capability with reported relevant anodes, d) Cycling stabilities of W‐P_L_@NPC, W‐P@NPC, and Nb/W‐P@NPC at 2.0 A g^−1^, e) Long cycling stabilities at 3.0 and 10.0 A g^−1^. Kinetics analyses of Nb/W‐P@NPC: f) Series CV curves and g) Capacitive contribution at various scan rates, h) Linear plots of Z′ versus *ω* ^−1/2^ of W‐P_L_@NPC, W‐P@NPC, and Nb/W‐P@NPC after 10 cycles at 2.4 V, and i) GITT profile indicating *D*_Na_^+^ during charging and discharging.

The samples are assembled into coin cells and evaluated for Na^+^ storage properties by electrochemical testing. In the initial cyclic voltammetry (CV) tests of Nb/W‐P@NPC at 0.3 mV s^−1^ (**Figure** **4**a), the reduction peaks at 0.93 and 0.45 V denote the formation of solid electrolyte interface (SEI) film on the electrode and the insertion of Na^+^ into Nb/W‐P clusters, respectively, and the oxidation peak at 0.33 V signifies the extraction of Na^+^.^[^ ^38^ ^]^ The close overlap of the subsequent CV curves initially verifies the high stability and reversibility of the electrode. Additional CV studies on W‐P@NPC and NP‐C deepen the understanding of the storage mechanisms (Figure S25, Supporting Information). The first galvanostatic charge–discharge (GCD) profiles at 0.1 A g^−1^ (Figure S26, Supporting Information) show that the reversible capacity of Nb/W‐P@NPC is as high as 556.3 mAh g^−1^, obviously surpassing that of W‐P@NPC (472.7 mAh g^−1^), W‐P_L_@NPC (385.2 mAh g^−1^), and NP‐C (296.7 mAh g^−1^), which emphasizes the efficacy of amorphous Nb/W‐P clusters in enriching storage sites. However, the initial coulombic efficiencies (≈50%) of all electrodes are unsatisfactory, probably due to the excessive consumption of Na^+^ by the side reactions during SEI formation.^[^ ^39^ ^]^ Strategies such as electrolyte modification and pre‐sodiation reportedly can alleviate this deficiency.^[^ ^40^, ^41^ ^]^ The rate capability of Nb/W‐P@NPC and corresponding smooth GCD curves (Figure 4b and Figure S27, Supporting Information) show a reversible capacity of up to 200.2 mAh g^−1^ even at 10.0 A g^−1^, with capacity virtually recovered upon current density reduction. The low‐coordination W‐P_L_@NPC benefits from high metal‐atom exposure for excellent rate capability, while the W‐P@NPC creates numerous storage sites via the high concentration of P‐bonding. The Nb/W‐P@NPC with bimetallic design further improves the storage properties of Na^+^ due to the Nb bridging and its high chemical potentials (Figure S28, Supporting Information), surpassing many reported anodes in terms of reversible capacity and rate capability (Figure 4c).^[^ ^20^, ^24^, ^26^, ^32^, ^38^, ^39^, ^42^, ^43^, ^44^ ^]^ Nb/W‐P@NPC also displays impressive cycling stability, maintaining 331.9 mAh g^−1^ reversible capacity after 2000 cycles at 2.0 A g^−1^ with 97.6% capacity retention and close to 100% Coulombic efficiency, superior to those of W‐P@NPC, W‐P_L_@NPC, and NP‐C (Figure 4d and Figure S29, Supporting Information). After 3000 cycles at 3.0 A g^−1^ (Figure 4e), Nb/W‐P@NPC maintains a high reversible capacity of 316.7 mAh g^−1^, with a capacity retention of nearly 100%. Especially at 10.0 A g^−1^, Nb/W‐P@NPC retains a reversible capacity of 190.6 mAh g^−1^ after 5000 cycles with a mean charging time of only 92.3 s (Figure S30, Supporting Information). Comparative analyses with reported anodes highlight the superior cycling stability of Nb/W‐P@NPC (Table S3, Supporting Information). These results suggest that the high coordination and Nb‐bridging of Nb/W‐P@NPC can effectively improve the storage site diversity, charge transfer efficiency and cycling stability of amorphous clusters, especially when combined with in situ carbon composites.

For insight into the storage kinetics of Na^+^ in Nb/W‐P@NPC, a series of CV tests are conducted at varying scan rates, with results shown in Figure 4f. Employing the power‐law relation: *i* = a*ν* ^b^, where *i* is the peak current, *ν* is the scan rate, and a and b are constants, the control mechanism of the reaction can be inferred from the logarithmic plot of *i* versus *ν*. Theoretically, the b‐value ranging from 0.5 to 1 implies diffusion control when close to 0.5 and surface capacitance dominance when nearing 1.^[^ ^42^, ^45^, ^46^ ^]^ As shown in Figure S31a (Supporting Information), the b‐value for Nb/W‐P@NPC approaches 1 (O1 = 0.89, R1 = 0.91), indicative of a predominant capacitive‐driven. At a set potential (*V*), the capacitive and diffusive contributions to the response current (*i*) can be quantified by the equation *i*(V) = k_1_ *v* + k_2_ *v* ^1/2^, where k_1_ *v* and k_2_ *v* ^1/2^ denote capacitive and diffusive currents, respectively.^[^ ^43^, ^47^ ^]^ At 0.8 mV s^−1^, the capacitive ratio of Nb/W‐P@NPC is 86.7% (Figure S31b, Supporting Information), rising from an initial 71.7–93.6% with escalating scan rate (Figure 4g). Comparisons with W‐P@NPC and W‐P_L_@NPC (Figures S32 and S33, Supporting Information) reveal that low‐coordinated clusters can facilitate ion mobility, while the bridging of Nb effectively improves charge transfer in the high‐coordination clusters. Electrochemical impedance spectroscopy (EIS) and galvanostatic intermittent titration technique (GITT) further assess the ion‐diffusion kinetics. In the Nyquist plots at 2.4 V (Figure S34, Supporting Information), the high‐frequency semicircle represents charge transfer resistance (*R*_ct_), while the low‐frequency sloping line denotes Warburg impedance (*W*).^[^ ^44^ ^]^ Fitting Z' versus *ω* ^−1/2^ based on the equivalent circuit (Figure 4h and Figure S34, Supporting Information) yields the *R*_ct_ and slope (*σ*) values of 123.6 Ω and 61.1 for Nb/W‐P@NPC, respectively, outperforming those of W‐P@NPC and W‐P_L_@NPC. The diffusion coefficient of Na^+^ (*D*_Na_⁺) can be analyzed from Equations (S4) and (S5) (Supporting Information), where the larger *D*_Na_⁺ corresponds to the smaller *σ* ^−0.5^, indicating enhanced diffusion with decreasing *σ*.^[^ ^48^ ^]^ The *D*_Na_⁺ can be further derived from GITT tests, as illustrated in Figure 4i and Figure S35 (Supporting Information).^[^ ^49^ ^]^ Using Fick's second law (Equation (S6), Supporting Information), the calculated *D*_Na_⁺ for Nb/W‐P@NPC ranges from 10^−8^ to 10^−10^ cm^2^ s^−1^, exceeding the range of 10^−10^ to 10^−12^ cm^2^ s^−1^ observed in W‐P@NPC. These findings indicate that the Nb‐bridging can optimize charge transfer efficiency of high‐coordination clusters, manifesting exceptional reaction kinetics during the electrochemical processes.

**Figure 5:**
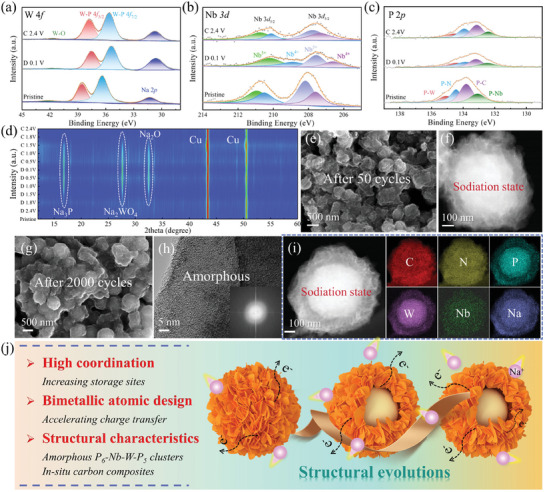
Ex situ characterizations of Nb/W‐P@NPC for SIHCs: HR‐XPS spectra for a) W 4*f*, b) Nb 3*d*, and c) P 2*p* at different sodiation states, d) Contour plot of XRD patterns at various potentials before cycle (Pristine) and after 10 cycles. After 50 cycles at 0.1 V: e) SEM image and f) HAADF‐STEM image. After 2000 cycles at 0.1 V: g) SEM image, h) TEM image with FFT image (inset), i) HAADF‐STEM image and Corresponding elemental mappings. j) Summary of the performance features and structural evolutions of Nb/W‐P@NPC during cycling.

Integrating ex situ characterization techniques enables a profound understanding of the Na^+^ storage mechanisms in Nb/W‐P@NPC. The XPS results of the electrode at different potentials are depicted in **Figures** **5**a–c and S36 (Supporting Information). In the HR W 4*f* spectra (Figure 5a), the characteristic peaks of W‐P 4*f*_5/2_ and W‐P 4*f*_7/2_ are shifted toward low binding energies in comparison with their initial positions during discharging to 0.1 V, with partial recovery upon recharge to 2.4 V, suggesting that the sodiation reaction alters the valence states of the W sites. The Na 2*p* peak at 31.2 eV may be due to the SEI formation on the electrode. Similar shifting trends are observed in the Nb 3*d* and P 2*p* peaks (Figure 5b,c), but with marked intensity variations, implying the active participation of Nb and P in Na^+^ storage via conversion reactions. Fluctuations in C 1*s* peak intensities without positional shifts (Figure S36a, Supporting Information) suggest an intercalation‐type mechanism for NP‐C in the electrode. The O 1*s* peaks are slightly shifted but obviously enhanced in intensity, indicating some changes in the valency and influenced by the oxygen‐containing groups in the SEI film and the oxygen adsorbed on the electrode surface (Nb/W‐O) (Figure S36b, Supporting Information). The ex situ XRD patterns (Figure 5d and Figure S37, Supporting Information) outline the apparent phase transitions of Nb/W‐P@NPC during charging and discharging, verifying the transitions from an initially amorphous state (as evidenced by standard peaks of the Cu substrate, PDF#85‐1326) to the emergence of new phases upon discharging. Specifically, the diffraction peaks emerging at 16.8°, 27.6°, and 32.5° can correspond to the generated Na_3_P, Na_2_WO_4_, and Na_2_O phases, respectively. The Na_3_P phase can stem from the conversion reaction of P: Nb/W‐P clusters +Na^+^ →Na_3_P+Nb/W atoms, while the Na_2_WO_4_ and Na_2_O phases reflect the slight surface oxidation (W‐O+Na^+^→Na_2_WO_4_ and W‐O+Na^+^→Na_2_O+W). Noticeably, no crystalline peaks for Nb and W phases are observed, suggesting that even after the conversion of the Nb/W‐P clusters, the Nb/W atoms remain in an amorphous state, with their crystallinity insufficient to produce XRD signals. These phenomena confirm the high electrochemical reversibility of the electrode. Thus, the spectral analyses systematically elucidate the storage mechanisms of Nb/W‐P@NPC, and validate that the high‐coordination bimetallic amorphous clusters can realize efficient Na^+^ storage.

The structural evolutions of Nb/W‐P@NPC during cycling can be effectively observed using ex situ SEM, TEM and related techniques. After 50 cycles and discharging to 0.1 V (discharging state), the SEM and TEM images (Figure 5e and Figure S38a, Supporting Information) show that the electrode still maintains the sheet‐sphere architecture uniformly wrapped by the SEI film. Comparison of the HAADF‐STEM image (Figure 5f) with the initial state (Figure 1c) reveal a tendency for amorphous Nb/W‐P clusters to migrate toward spherical centers. Even after 2000 cycles at the discharging state (Figure 5 g and Figure S39a, Supporting Information), the sheet‐sphere morphology remains intact. HAADF‐STEM image (Figure S39b, Supporting Information) further illustrates the accumulation of Nb/W‐P clusters around the spherical centers, highlighting the internal restructuring of the electrode during cycling. Notably, after various cycles, HR‐TEM images (Figure 5h and Figure S38b, Supporting Information) and their FFT images (inset) show persistent amorphous features with minimal volume changes. These observations suggest that the clusters are highly resistant to mechanical damage and can effectively relieve the structural stresses generated in Na^+^ storage, thus ensuring high structural and cycling stability. Elemental mapping (Figure 5i and Figure S39b, inset, Supporting Information) depicts a uniform dispersion of C, N and O across the electrode, while W, Nb, P, and Na are concentrated around the spherical centers, and the different contents of the two regions are listed in Table S4 (Supporting Information). These results suggest that Nb/W‐P@NPC experiences obvious structural transitions during cycling, but its amorphous clusters and in situ carbon are uniquely efficacious in maintaining structural integrity. Progressively, cycling‐induced surface remodeling of the electrode (enriched with carbon nanosheets) can offer efficient charge transfer pathways for the center‐nested Nb/W‐P clusters, thus relieving volume variations and maintaining exceptional storage properties. Figure 5j succinctly summaries the performance characteristics and structural evolutions of Nb/W‐P@NPC, re‐emphasizing the importance of high‐coordination amorphous clusters and bimetallic atom configurations in advancing Na^+^ storage.

### 2.4 Hybrid Capacitor Application

**Figure 6:**
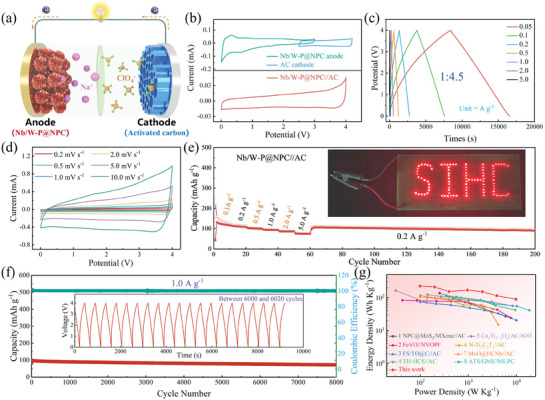
Utility of Nb/W‐P@NPC//AC SIHCs: a) Device schematic, b) CV curves of Nb/W‐P@NPC anode and commercial AC cathode in half cells (top) and full cell (bottom) at 1.0 mV s^−1^, c) GCD curves at various current densities and d) CV curves at different scan rates, e) Rate capability (Inset: LED bulbs powered by the SIHCs), f) Long cycling stability at 1.0 A g^−1^ with GCD curves (inset). g) Energy density and power output of the SIHCs compared to reported literatures.

Employing Nb/W‐P@NPC as the battery‐type anode and the commercially available activated carbon (AC) as the capacitive cathode, Nb/W‐P@NPC//AC SIHCs are fabricated and evaluated for practical applications. As presented in Figure S40 (Supporting Information), the quasi‐rectangular CV and triangular GCD curves of AC demonstrate clear capacitive adsorption‐desorption reaction mechanisms. Combined with its superior rate capability and long cycling stability, these features further indicate that AC is an ideal cathode for SIHCs. The SIHC schematic is depicted in **Figure** **6**a, where ClO_4_^‐^ ions are primarily adsorbed/desorbed on the AC surfaces, coinciding with insertion/extraction of Na⁺ in the anode. Based on the principles of capacity matching and minimizing side reactions, the voltage ranges of 0–4.0 V (Figure 6b and Figure S41, Supporting Information) and the anode‐to‐cathode mass ratio of 1:4.5 (Figure 6c, Figures S42 and S43, Supporting Information) are the optimal parameters. The CV curves of the electrodes (Figure 6b) show a hybrid storage mechanism that integrates both battery‐ and capacitor‐type characteristics in the SIHCs. The quasi‐triangular GCD curves (Figure 6c) and rectangle‐like CV curves (Figure 6d) also confirm the storage mechanisms. In terms of the total electrode mass, the SIHCs exhibit a high energy density of 223.6 Wh kg^−1^ at a power density of 99.8 W kg^−1^, maintaining an energy density of 89.3 Wh kg^−1^ even at 9800 W kg^−1^ (Figure 6e). After 8000 cycles at 1.0 A g^−1^, the device retains 73.4% of its initial capacity, with an average loss of only 0.0033% per cycle (Figure 6f). These storage properties exceed many reported SIHCs and energy storage devices (Figure 6g, Table S5 and Figure S44, Supporting Information).^[^ ^50^, ^51^, ^52^, ^53^, ^54^, ^55^, ^56^, ^57^, ^58^ ^]^ Remarkably, the SIHC effortlessly illuminates a logo consisting of 90 LED bulbs (inset, Figure 6e), exemplifying the practicality of Nb/W‐P@NPC. These findings indicate that the amorphous Nb/W‐P clusters effectively enhance storage sites and transport kinetics of the electrode, thereby enabling SIHCs with high energy density and high‐power density.

## 3 Conclusions

In conclusion, the distinctive amorphous Nb/W‐P clusters within NP‐C (Nb/W‐P@NPC) are fabricated by the bimetallic design with high coordination, and their reaction mechanisms and application potentials in Na^+^ storage are dissected. In this composite, the clusters are naturally characterized by high atom utilization and strong structural resilience, and contribute extra storage sites due to the high coordination of P atoms. Meanwhile, the high‐potential Nb‐bridging in the second shell of the W‐P_5_ clusters can effectively optimize adsorption energies and weaken the *d‐p* orbital interactions between W and P, elevate the *d*‐band center and downshift the *p*‐band center, thus facilitating charge transport and enriching storage sites. As expected, Nb/W‐P@NPC manifests exceptional anode properties, including a reversible capacity of up to 556.3 mAh g^−1^ at 0.1 A g^−1^, and impressive long cycling stability (190.6 mAh g^−1^ after 5000 cycles at 10.0 A g^−1^, with 92.3% capacity retention). The anode effectively relieves the kinetic mismatch with the AC cathode, yielding both high energy density (223.6 Wh kg^−1^) and high‐power output (9800 W kg^−1^) in SIHCs. Thus, the findings unveil the storage mechanisms of Na^+^ in high‐coordination bimetallic amorphous clusters, thereby charting a new course for the design of high‐performance electrode materials.

## Conflict of Interest

The authors declare no competing financial interest.

## Supporting information



Supporting Information

## Data Availability

The data that support the findings of this study are available from the corresponding author upon reasonable request.
