# Supplementary material for: High‐Coordination and Nb‐Bridging of Bimetallic Amorphous P6‐Nb‐W‐P5 Clusters in Carbon Nanospheres for High‐Performance Sodium‐Ion Hybrid Capacitors
Source: Adv Sci (Weinh). 2025 Jan 23;12(11):2416942. doi: 10.1002/advs.202416942 (PMC11923884; doi:10.1002/advs.202416942)
Supplement: Supplementary file 1 — Supporting Information [file ADVS-12-2416942-s001.docx]

**Supporting Information**

**High-Coordination and Nb-Bridging of Bimetallic Amorphous P_6_-Nb-W-P_5_ Clusters in Carbon Nanospheres for High-Performance Sodium-Ion Hybrid Capacitors**

*Shuxiao Hu* ^a^*,* *Baoquan Liu* ^a^*, Fanyan Zeng* ^a^**,* *Yang Pan* ^b^*,* *Dui Ma* ^a^**,* *Meilan Xie* ^a^*, Shenglian Luo ^c^**

^a^ School of Materials Science and Engineering, Nanchang Hangkong University, Nanchang 330063, Jiangxi, People’s Republic of China.

^b^ College of Life Science, Jiangxi Normal University, Nanchang 330022, Jiangxi, People’s Republic of China.

^c^ Key Laboratory of Jiangxi Province for Persistent Pollutants Control and Resources Recycle, Nanchang Hangkong University, Nanchang 330063, Jiangxi, People’s Republic of China.

E-mail: zfy1012@nchu.edu.cn (F. Y. Zeng), dui.ma@nchu.edu.cn (D. Ma), sllou@hnu.edu.cn (S. L. Luo)

**Experimental Section**

**Synthesis of Nb/W-polydopamine (Nb/W-PDA)** **nanospheres:** Initially, a flask containing 0.192 g of ammonium tungstate (W source) and 60 mL of deionized water was placed in an ion bath at 60 °C and stirred for 10 min. Subsequently, 0.048 g of ammonium niobate oxalate (Nb source) was poured in and stirred until dissolved, then 0.6 g of dopamine hydrochloride was added and the solution was sonicated for 20 min. Finally, 120 mL of anhydrous ethanol was slowly added to the solution and stirred for 50 min, followed by a slow trickle of 8 mL of ammonia water and kept at 60 °C for 24 h, yielding a brownish suspension. After repeated washing with deionized water and filtration, the Nb/W-PDA nanospheres were dried in oven-drying at 90 °C for 12 h. Additionally, the Nb/W-PDA samples with different Nb contents were prepared by altering the mass ratio of Nb/W sources under the same conditions. In addition, the pure W-PDA nanosheets, Nb-PDA nanospheres, and PDA materials were also synthesized without Nb source, W source, or both metal sources, respectively.

**Preparation** **of Nb/W-P@NPC:** Using Nb/W-PDA nanospheres with 20% Nb content as the precursors, Nb/W-PDA and NaH_2_PO_2_·H_2_O were weighed in a 1:10 mass ratio and placed in two quartz boats separately. Under Ar environment, the Nb/W-P@NPC was obtained by annealing at 600 ℃ for 2 h. Taking W-PDA as the precursors, W-P_L_@NPC (low coordination) and W-P@NPC (high coordination) were prepared by placing W-PDA and NaH_2_PO_2_·H_2_O (P source) in two quartz boats with the mass ratios of 2:5 and 1:10, respectively, and subjected to the same annealing treatment.

**Syntheses of NP-C, NP-C_PDA_ and NC:** The Nb/W-PDA nanospheres were first annealed at 500 °C for 2 h under Ar atmosphere. The Nb/W-P clusters were then selectively etched with hydrochloric acid (37%) and ammonia (28%) diluted with water at a 1:1 volume ratio, respectively, to remove the Nb and W components. After filtration and drying, NP-C was obtained by the above phosphating method (1:10 ratio), while NP-C_PDA_ and NC were prepared by treating PDA at 600 °C for 2 h with phosphating (1:10 ratio) and without P source, respectively.

**Material characterization:** Morphological and microstructural features of samples were examined by field-emission scanning electron microscopy (SEM, FEI Nova Nano SEM450) and transmission electron microscopy (TEM, FEI Talos F200X). Elemental compositions of samples were carried out via an energy dispersive X-ray (EDX) spectrometer integrated with the F200X. Atomic distribution in clusters was depicted by aberration-corrected high-angle annular dark field scanning transmission electron microscopy (AC HAADF-STEM, Thermo Scientific™ Themis Z). Phase structures of samples were identified by X-ray diffraction (XRD, Brucker D8-Advanced) and Raman spectroscopy (LabRam-010). Vacancy defects in samples were detected by electron paramagnetic resonance spectroscopy (EPR, Bruke emxplus). Porosity details of samples were quantified by Beckman Coulter SA-3100 analyzer. Elemental composition and mass contents of samples were analyzed by inductively coupled plasma (ICP, Agilent 720) and thermogravimetric testing (TG, Netzsch STA 449F3). Surface chemical states of samples were probed by X-ray photoelectron spectroscopy (XPS, Escalab 250Xi). Coordination environments of samples were analyzed by hard X-ray absorption spectroscopy (XAS) at the W L-edge and Nb K-edge using a Si (111) crystal monochromator at the BL14W beamline at the Shanghai Synchrotron Radiation Facility (SSRF). Before the synchrotron analysis, the samples were placed into aluminum sample holders and sealed using Kapton tape film. The XAS spectra were recorded at room temperature using a 4-channel Silicon Drift Detector (SDD) Bruker 5040.

**Electrochemical test:** The active materials were highly dispersed in 1-methyl-2-pyrrolidone (NMP) along with polyvinylidene fluoride (PVDF) and carbon black (Super-P) in a mass ratio of 7:2:1. Intense stirring ensured a uniform slurry, which was then evenly coated onto a copper foil and dried at 100 °C for 12 h. The foil was subsequently cut into 12-mm diameter circular slices, each with an active material loading ranging from 0.8 to 1.2 mg cm^-2^. Sodium metal served as the counter electrodes, glass fibers (GF/D, Whatman) as the separators, and 1 M NaClO_4_ in a 1:1 volume ratio of ethylene carbonate (EC) and dimethyl carbonate (DMC) with 5.0% fluoroethylene carbonate (FEC) constituted the electrolyte. The CR2032 coin cells were assembled in an Ar-filled glove box, and anode performance was assessed within the voltage window of 0.005-3 V (*vs.* Na^+^/Na). Cyclic voltammetry (CV) and electrochemical impedance spectroscopy (EIS) were conducted using a CHI 660E workstation. Galvanostatic charge-discharge (GCD) and galvanostatic intermittent titration technique (GITT) curves were recorded on a battery tester (Neware, CT4008Tn). For post-cycling structural analyses, the cells were disassembled after cycles and the electrodes were cleansed with dimethyl carbonate. *Ex-situ* techniques, such as XPS, XRD, SEM and TEM, were served to elucidate the storage mechanisms and structural evolutions. In the fabrication of sodium-ion hybrid capacitors (SIHCs), Nb/W-P@NPC (anode) was terminated at 0.005 V after 10 cycles at 0.2 A g^-1^. Commercial activated carbon (AC) as the cathode was similarly prepared on an Al foil. Following capacity matching and performance optimization, an anode-to-cathode mass ratio of 1:4.5 was determined, leading to the assembly of the SIHCs labeled as Nb/W-P@NPC//AC, and the equations for energy density (E) and power density (P) of SIHCs are given below:

P=Δ*Vi* (S1)

E=*Pt*/3600=*ΔVit* (S2)

Δ*V*=(*V*_max_+*V*_min_)/2 (S3)

where *t* is the discharge time (s), *i* is the current density (A g^-1^), and *V*_max_/*V*_min_ are the initial/final potentials of discharge curve excluding the *IR* drop.

**Theoretical calculation:** All density functional theory (DFT) computations were performed using the Vienna Ab Initio Simulation Package (VASP), adopting the generalized gradient approximation (GGA) with the Perdew-Burke-Ernzerhof (PBE) exchange-correlation functional ^[1-3]^. The description of ionic cores and treatment of valence electrons were accomplished by Projector Augmented Wave (PAW) potentials, employing a plane-wave basis set with an energy cutoff of 450 eV ^[4, 5]^. To handle fractional occupancies in the Kohn-Sham orbitals, the Gaussian smearing scheme with a broadening of 0.05 eV was implemented. Self-consistency in the electronic energy was attained when the energy difference fell below 10^-5^ eV, and geometric convergence was assumed when force changes were less than 0.03 eV Å^-1^. Dispersion interactions were accounted for using Grimme's DFT-D3 approach ^[6]^. The vacuum spacing perpendicular to the plane of the structure is 20 Å. The Brillouin zone integral utilized the surfaces structures of 2*2*1 monkhorst pack K-point sampling. Finally, the adsorption energies (*E*_ads_) were calculated as *E*_ads_=*E*_ad/sub_-*E*_ad_-*E*_sub_, where *E*_ad/sub_, *E*_ad_, and *E*_sub_ represent the total energies of the optimized adsorbate/substrate system, the isolated adsorbate, and the pristine substrate, respectively, after structural optimization.

**
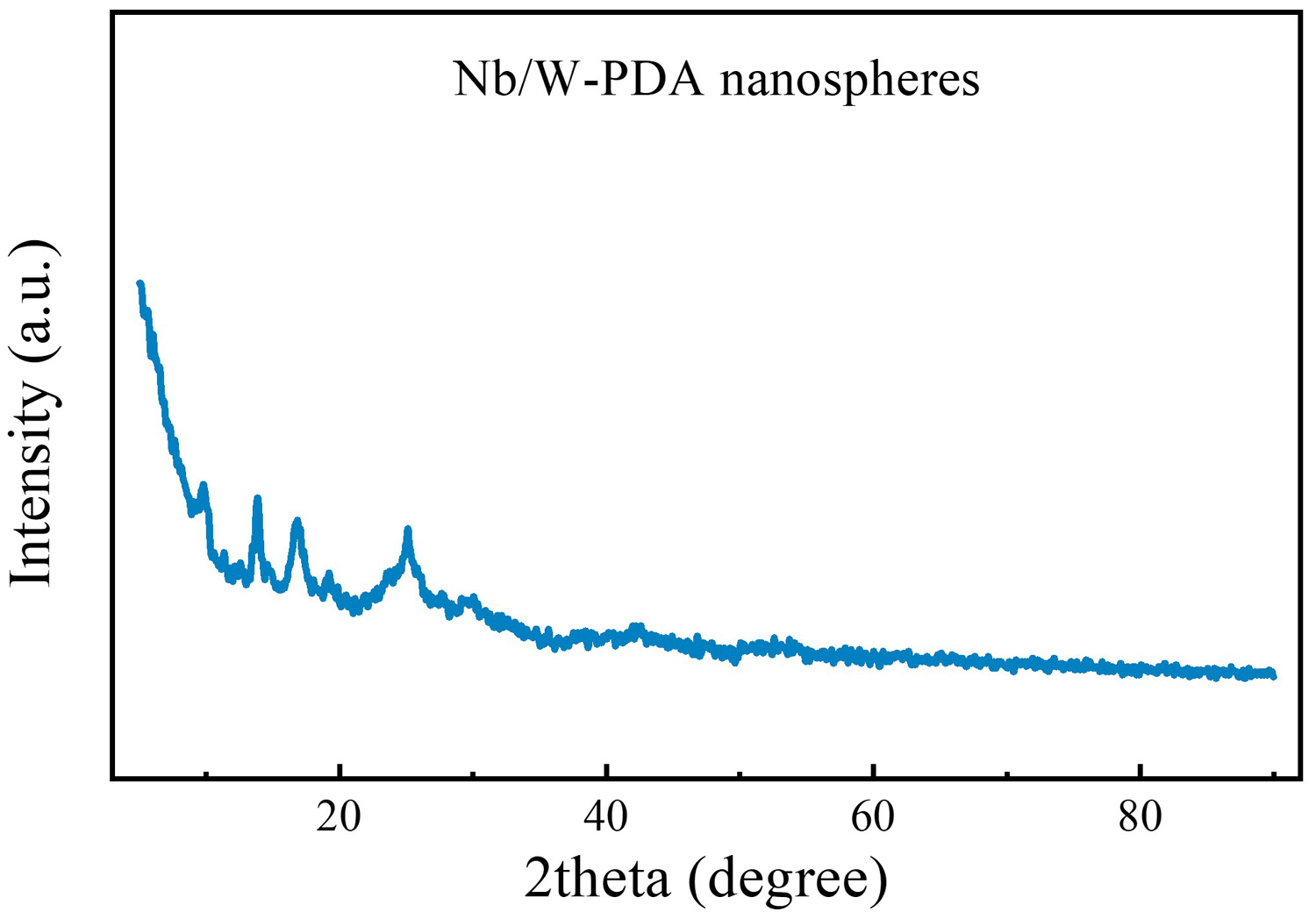
**

**Figure S1.** XRD pattern of Nb/W-PDA nanospheres.


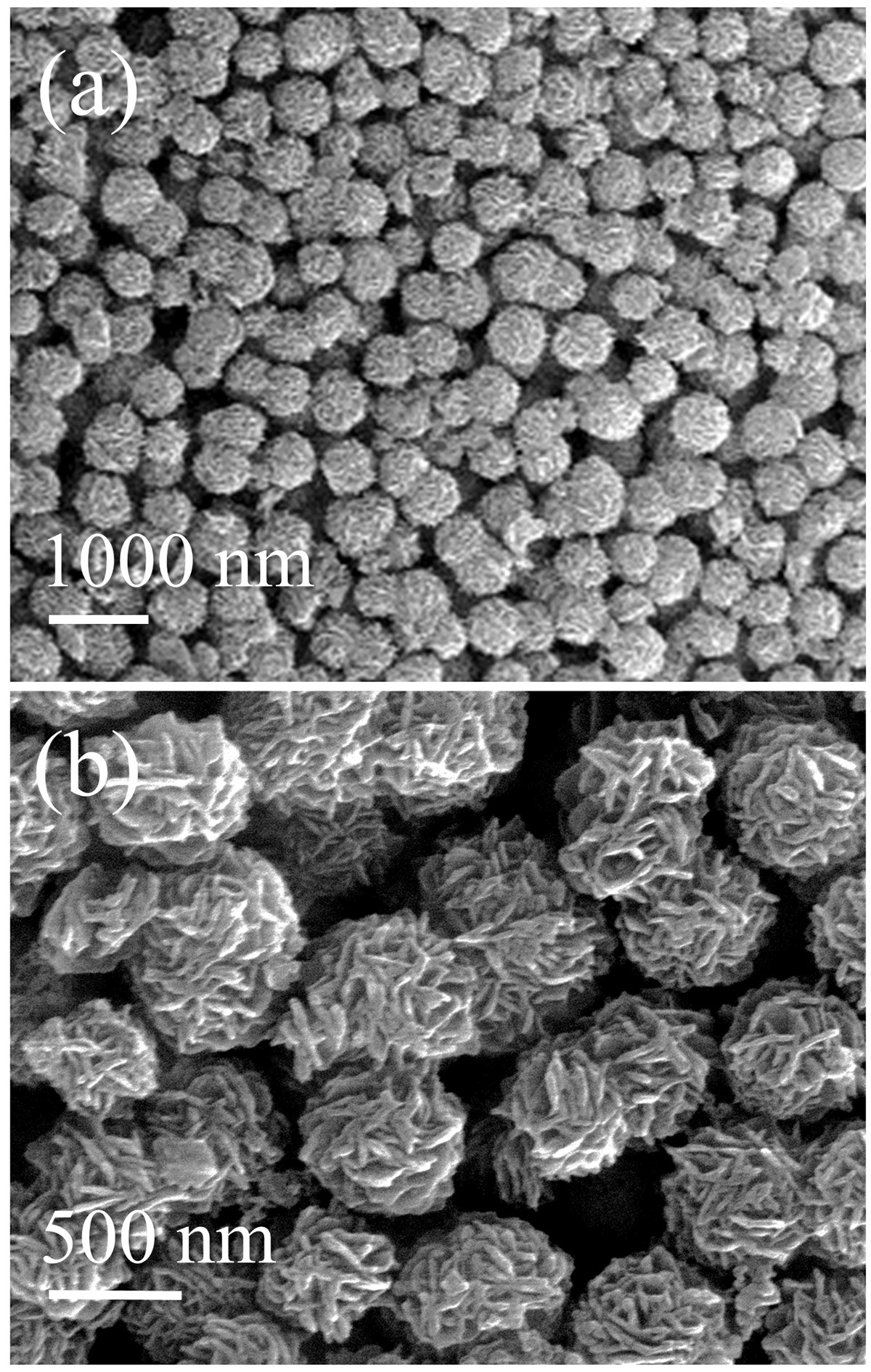


**Figure S2.** (a-b) SEM images of Nb/W-PDA nanospheres.


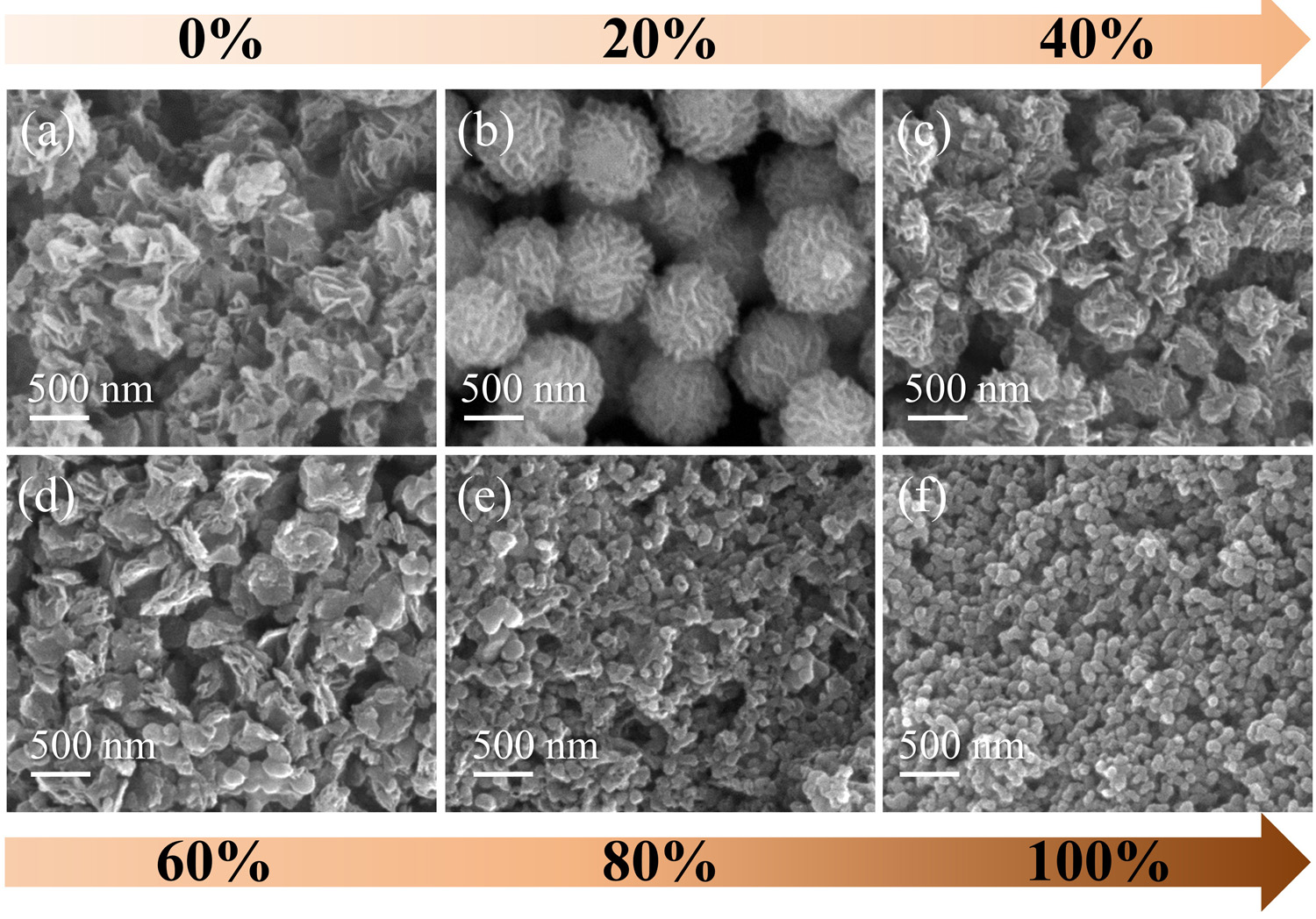


**Figure S3.** (a-f) SEM images of Nb/W-PDA with the different mass fraction of Nb source.


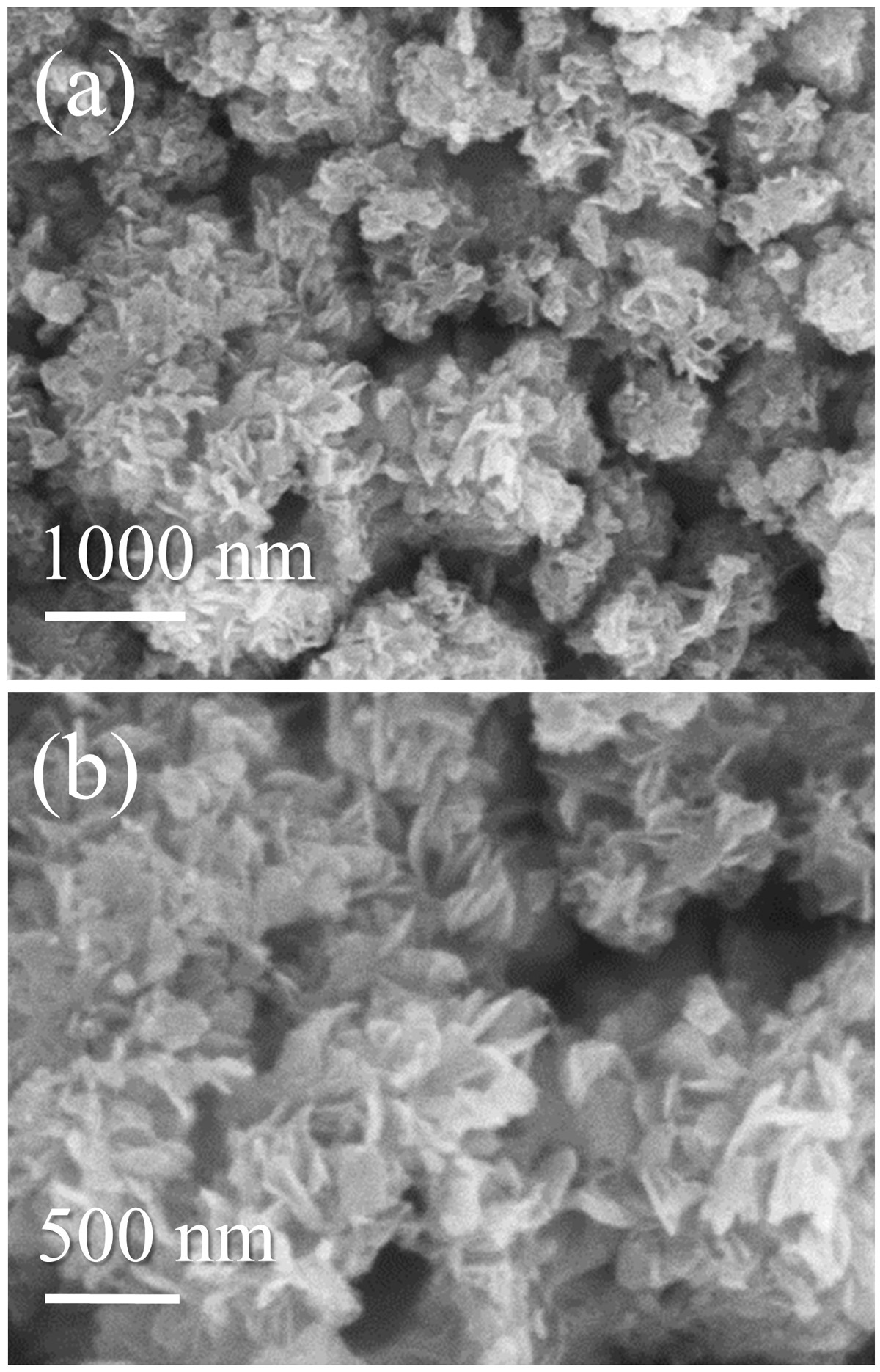


**Figure S4.** (a-b) SEM images of W-P@NPC.


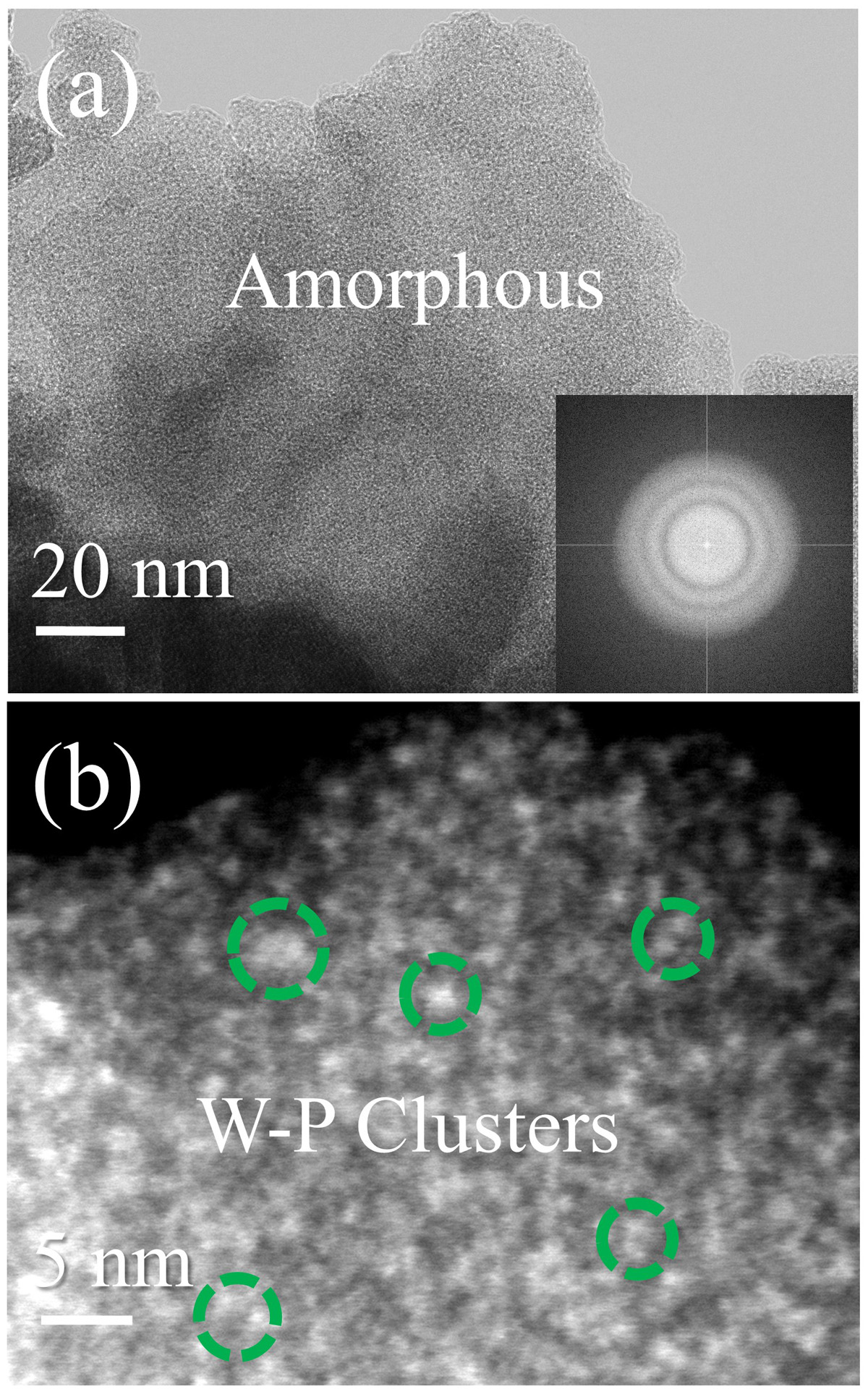


**Figure S5.** (a) TEM image with FFT image (inset) and (b) HAADF-STEM image of W-P@NPC.


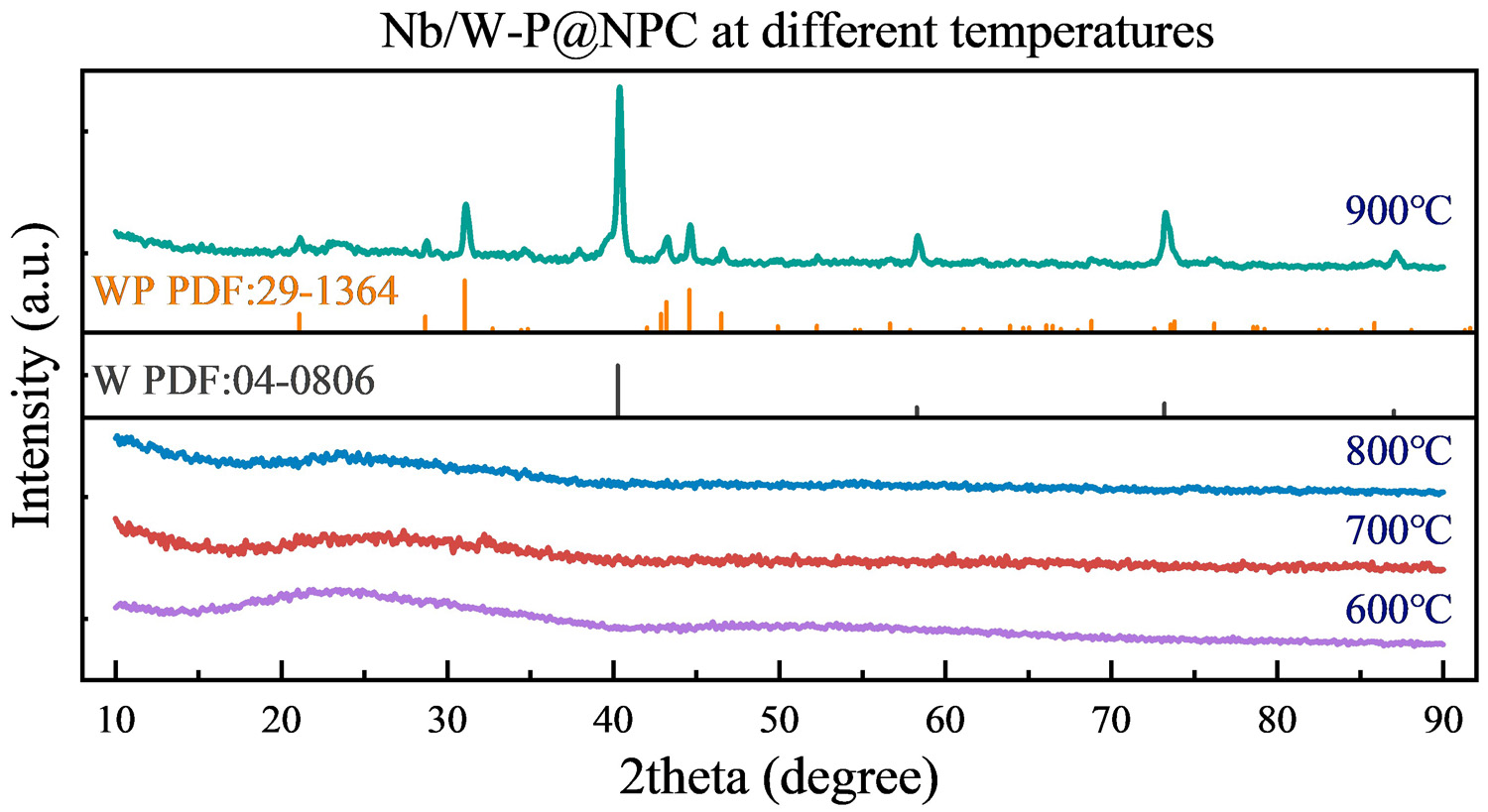


**Figure S6.** XRD patterns of Nb/W-P@NPC at different annealing temperatures.


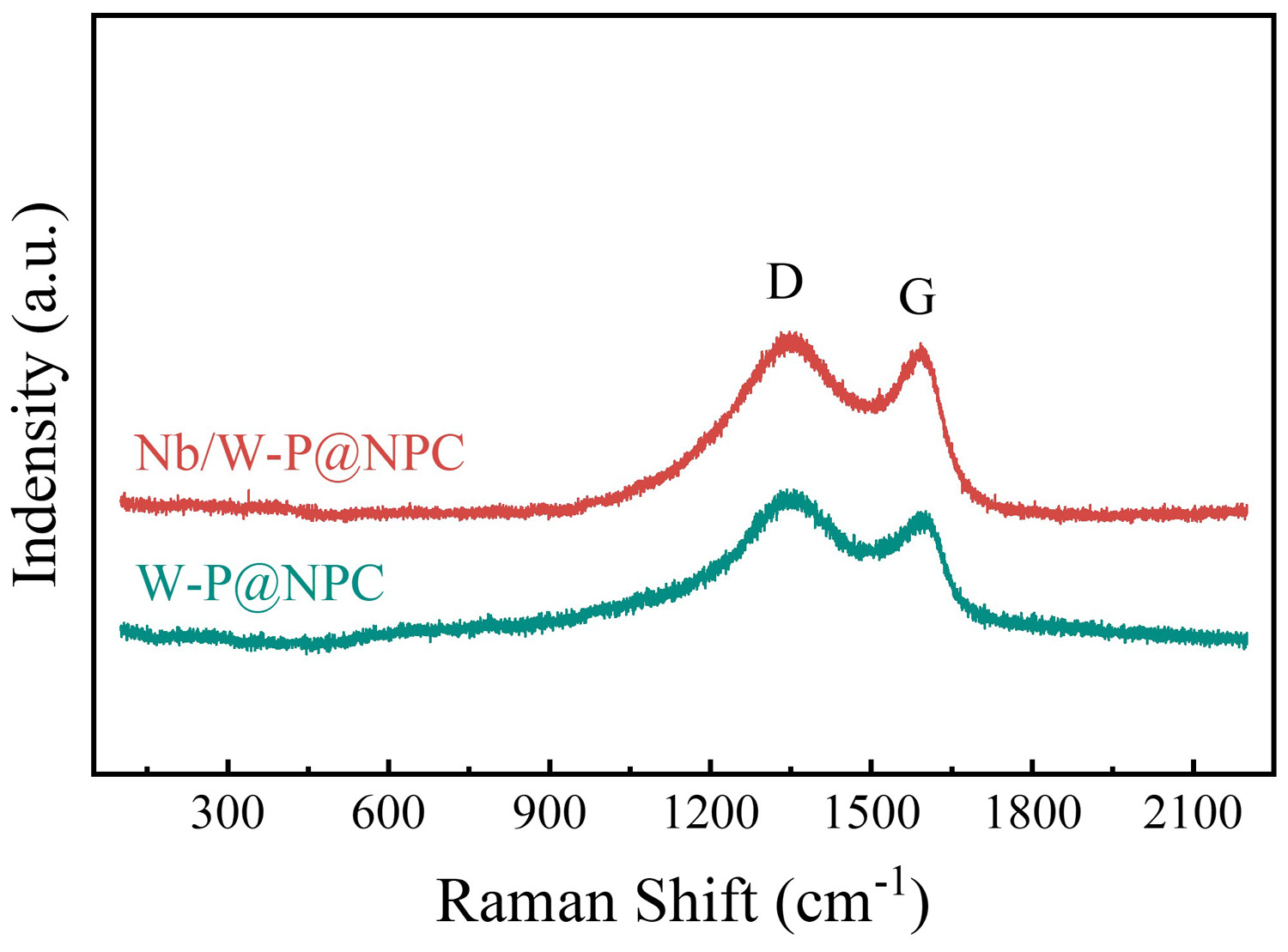


**Figure S7.** Raman spectra of W-P@NPC and Nb/W-P@NPC.


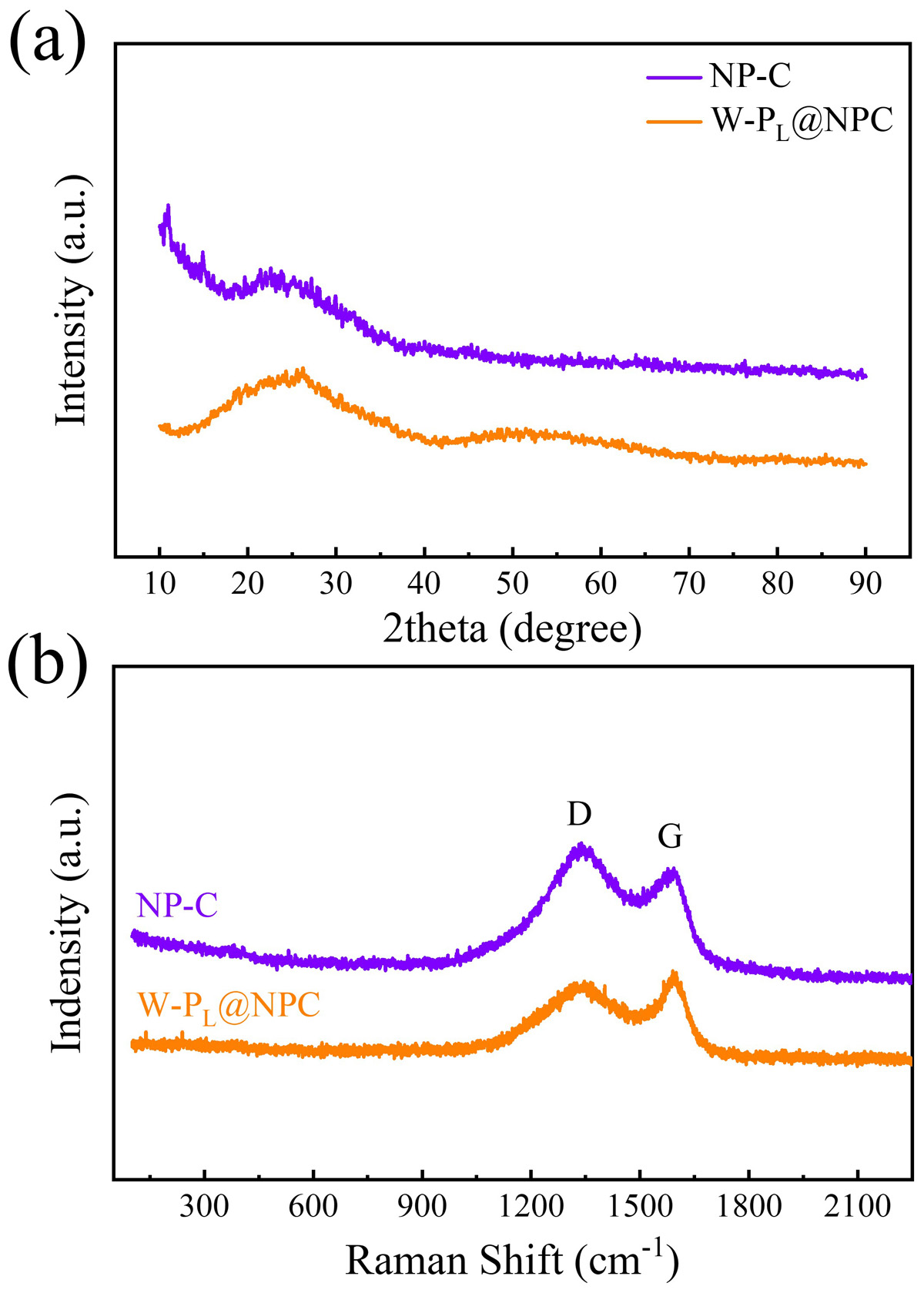


**Figure S8.** (a) XRD patterns and (b) Raman spectra of W-P_L_@NPC and NP-C.


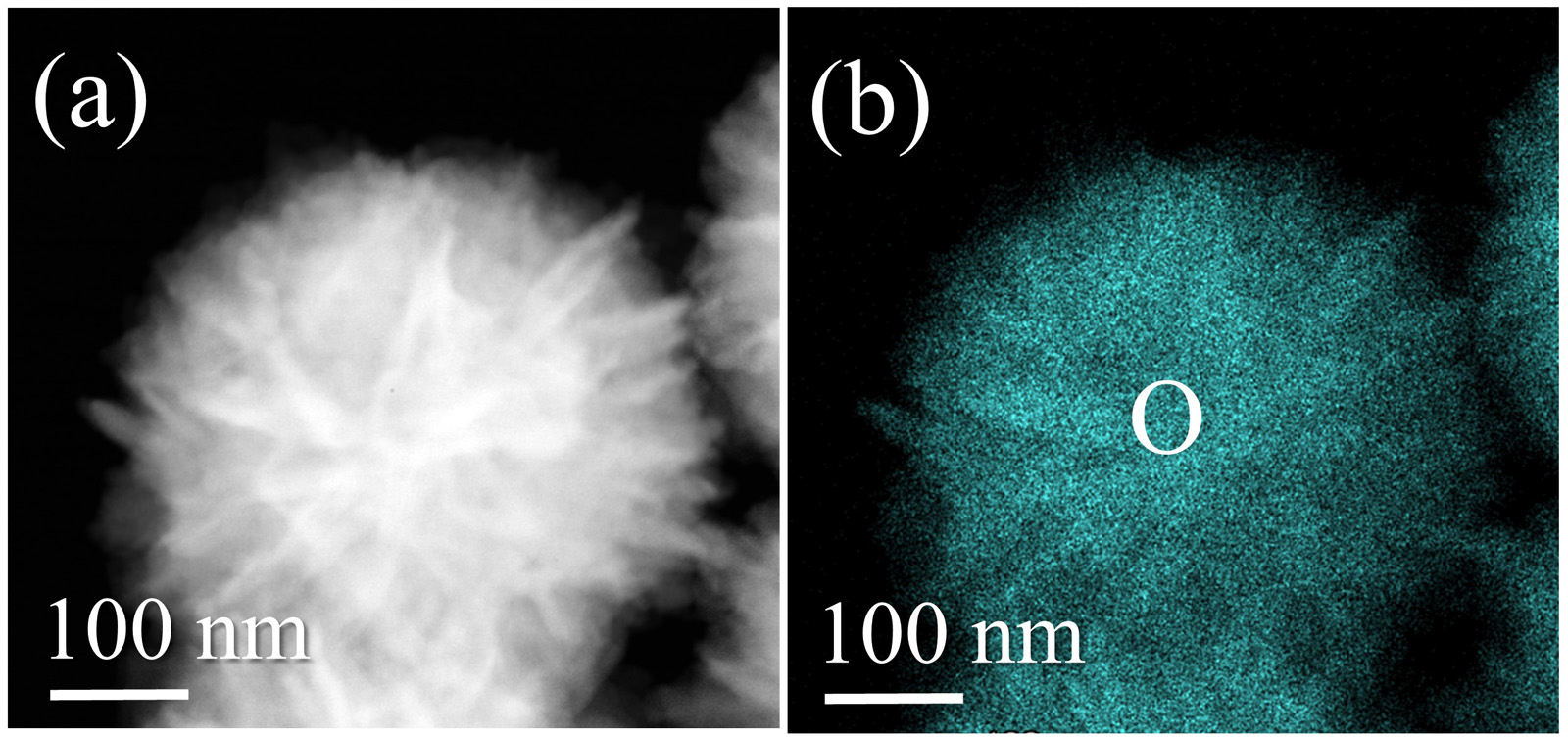


**Figure S9.** (a) HAADF-STEM image of Nb/W-P@NPC and (b) corresponding elemental mapping of O.


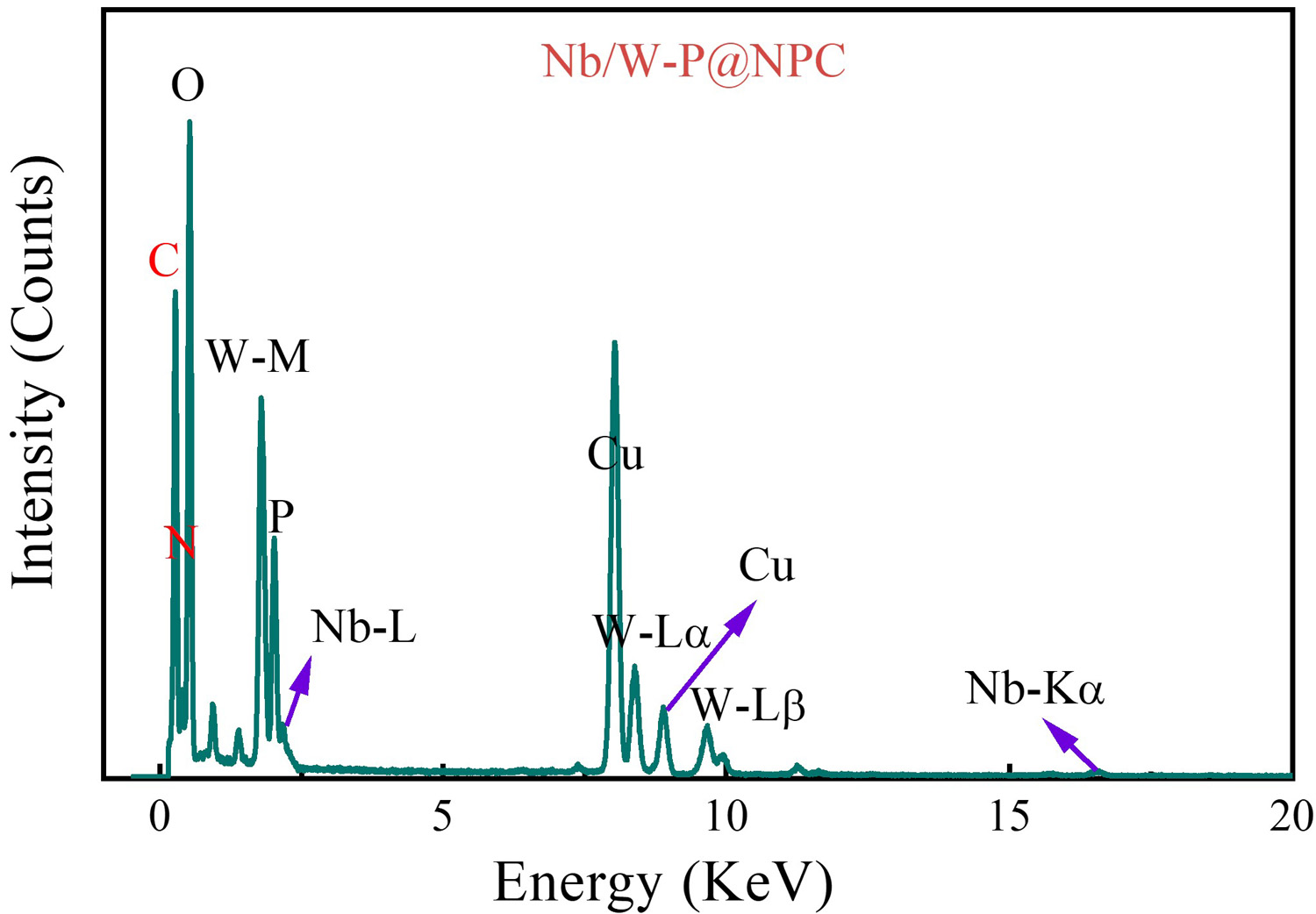


**Figure S10.** EDX spectrum of Nb/W-P@NPC.


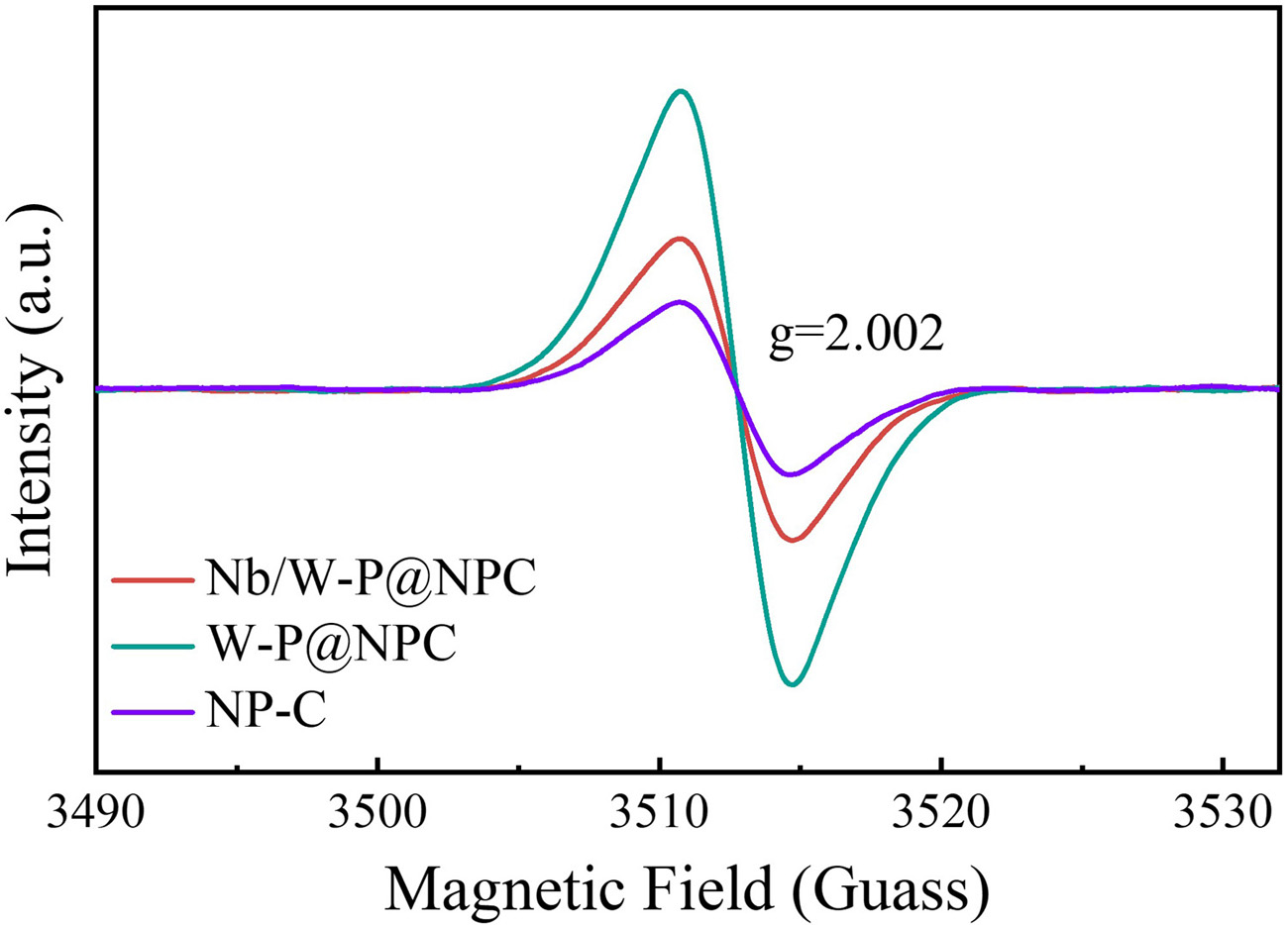


**Figure S11.** EPR spectra of NP-C, W-P@NPC and Nb/W-P@NPC.

**
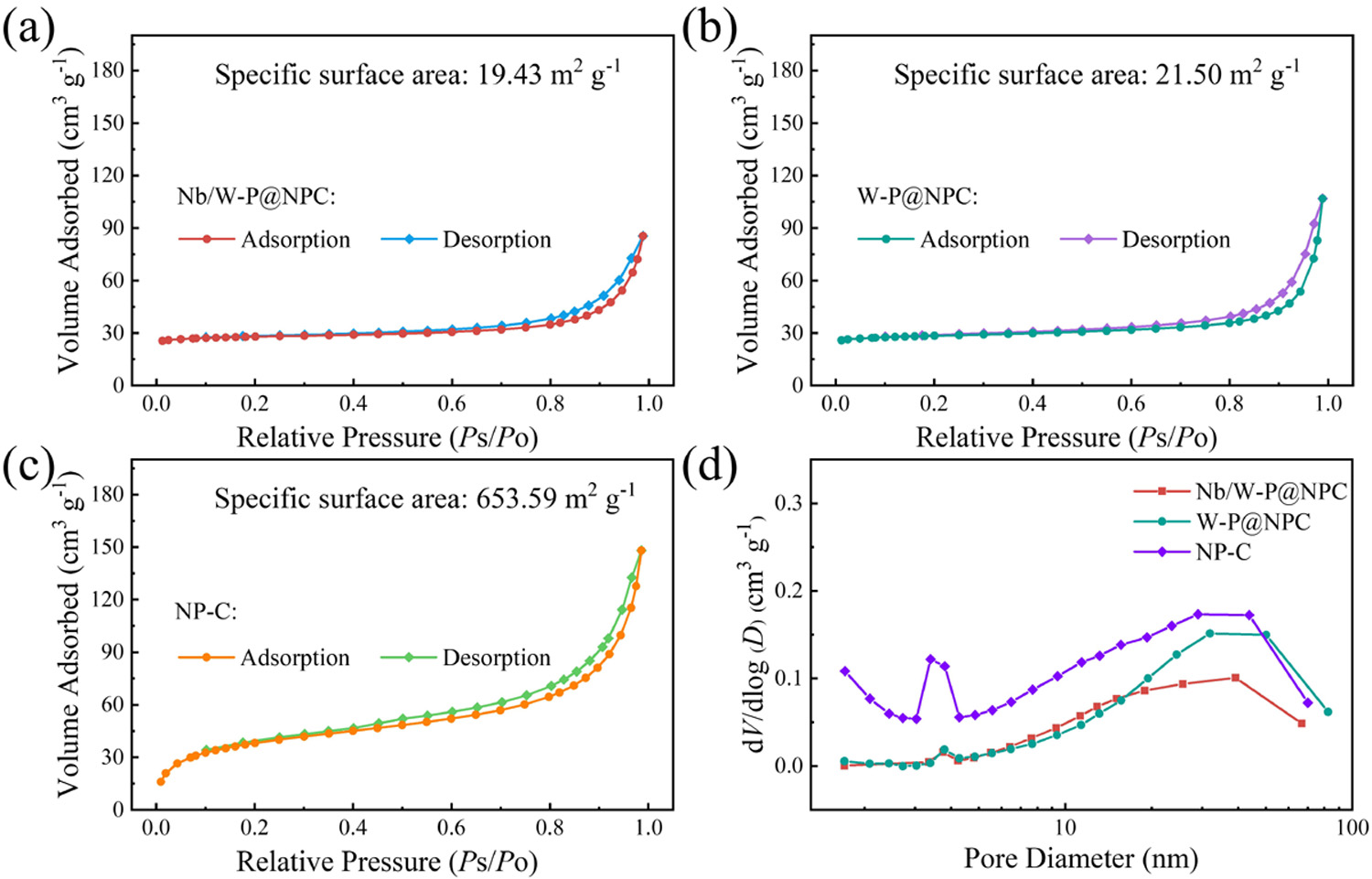
**

**Figure S12.** (a-c) N_2_ adsorption/desorption curves and (d) Pore-size distributions of Nb/W-P@NPC, W-P@NPC and NP-C.

**
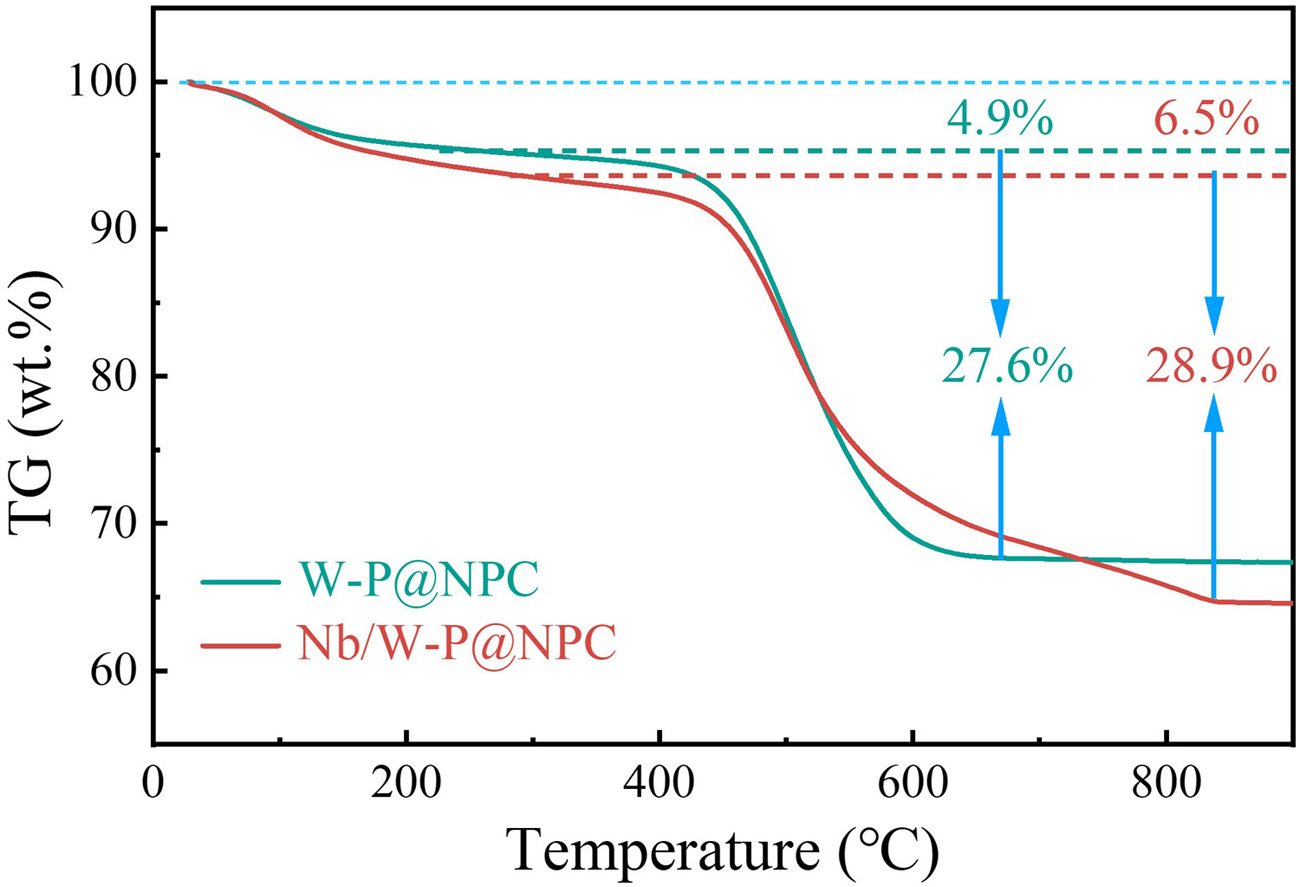
**

**Figure S13.** TG curves of Nb/W-P@NPC and W-P@NPC.

**
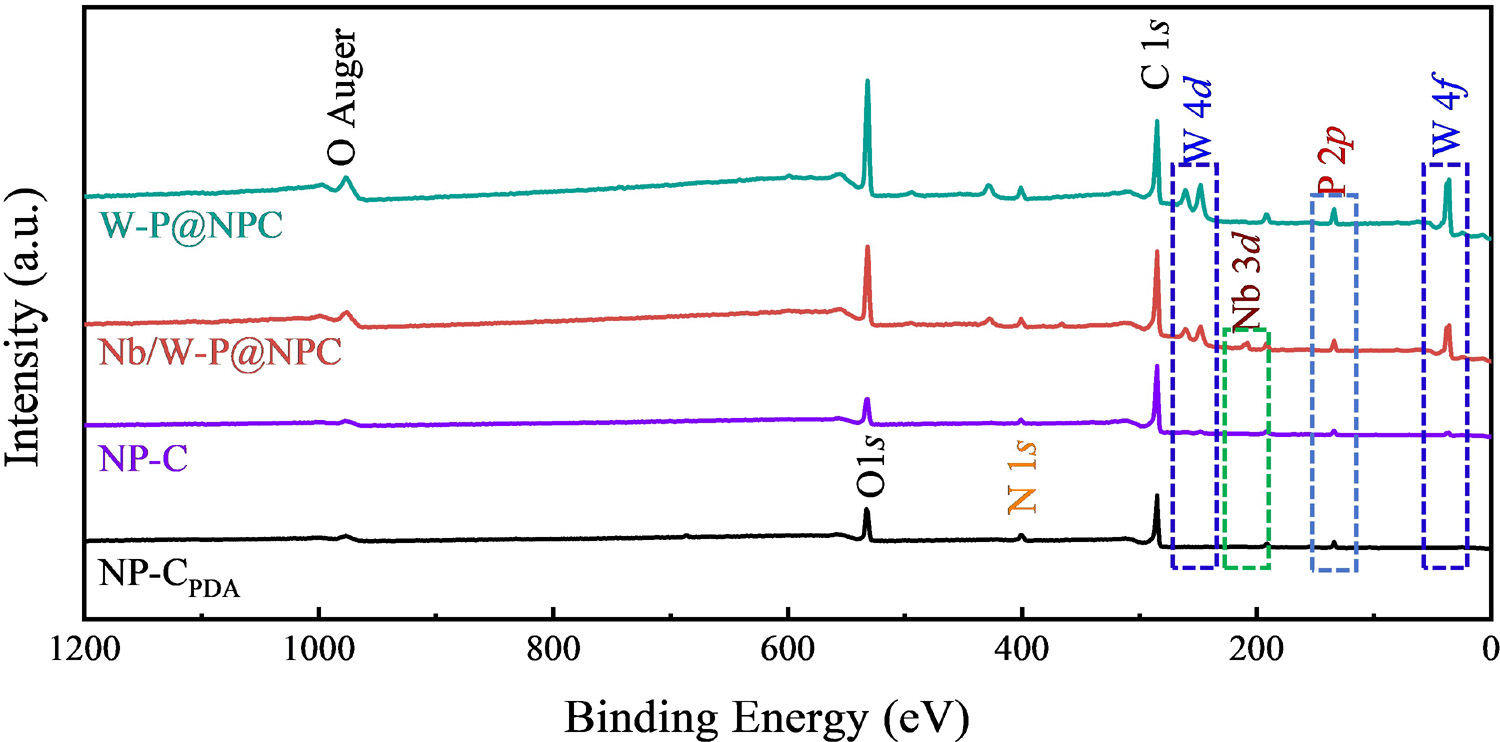
**

**Figure S14.** XPS survey spectra of NP-C_PDA_, NP-C, Nb/W-P@NPC and W-P@NPC.

**
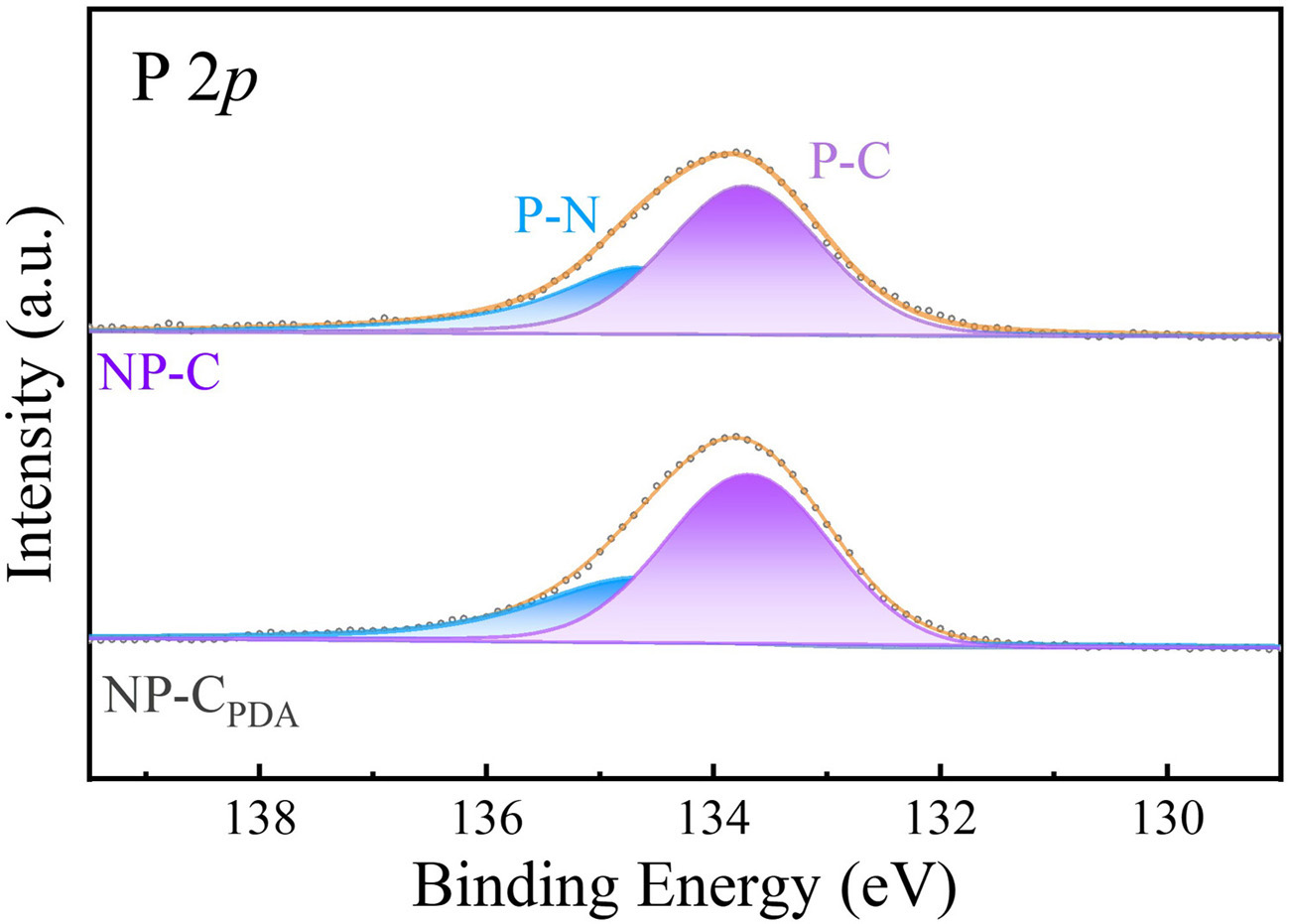
**

**Figure S15.** HR-XPS spectra of P 2*p* in NP-C_PDA_ and NP-C.

**
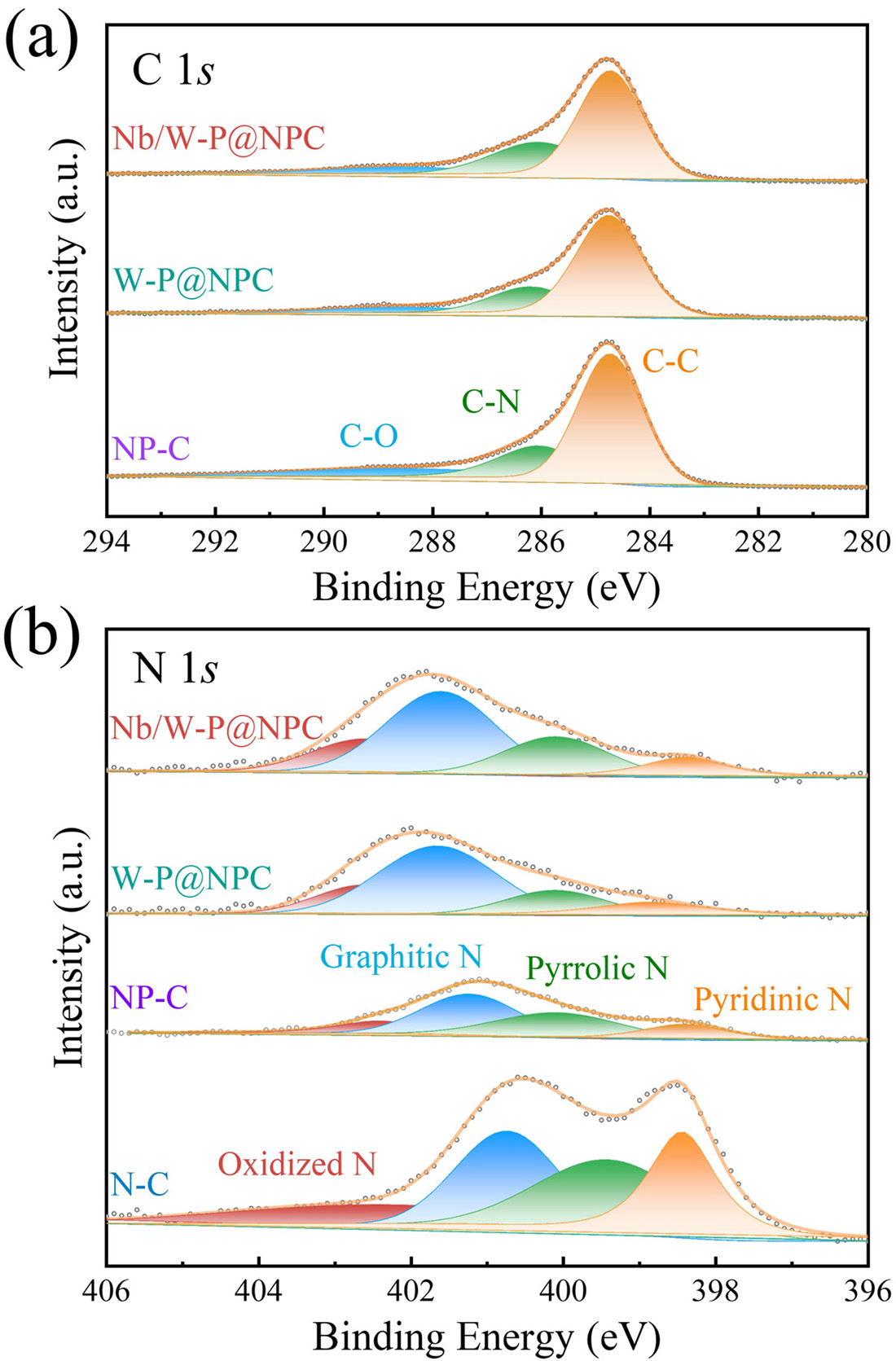
**

**Figure S16.** HR-XPS spectra of (a) C 1*s* in NP-C, W-P@NPC and Nb/W-P@NPC and (b) N 1*s* in N-C, NP-C, W-P@NPC and Nb/W-P@NPC.


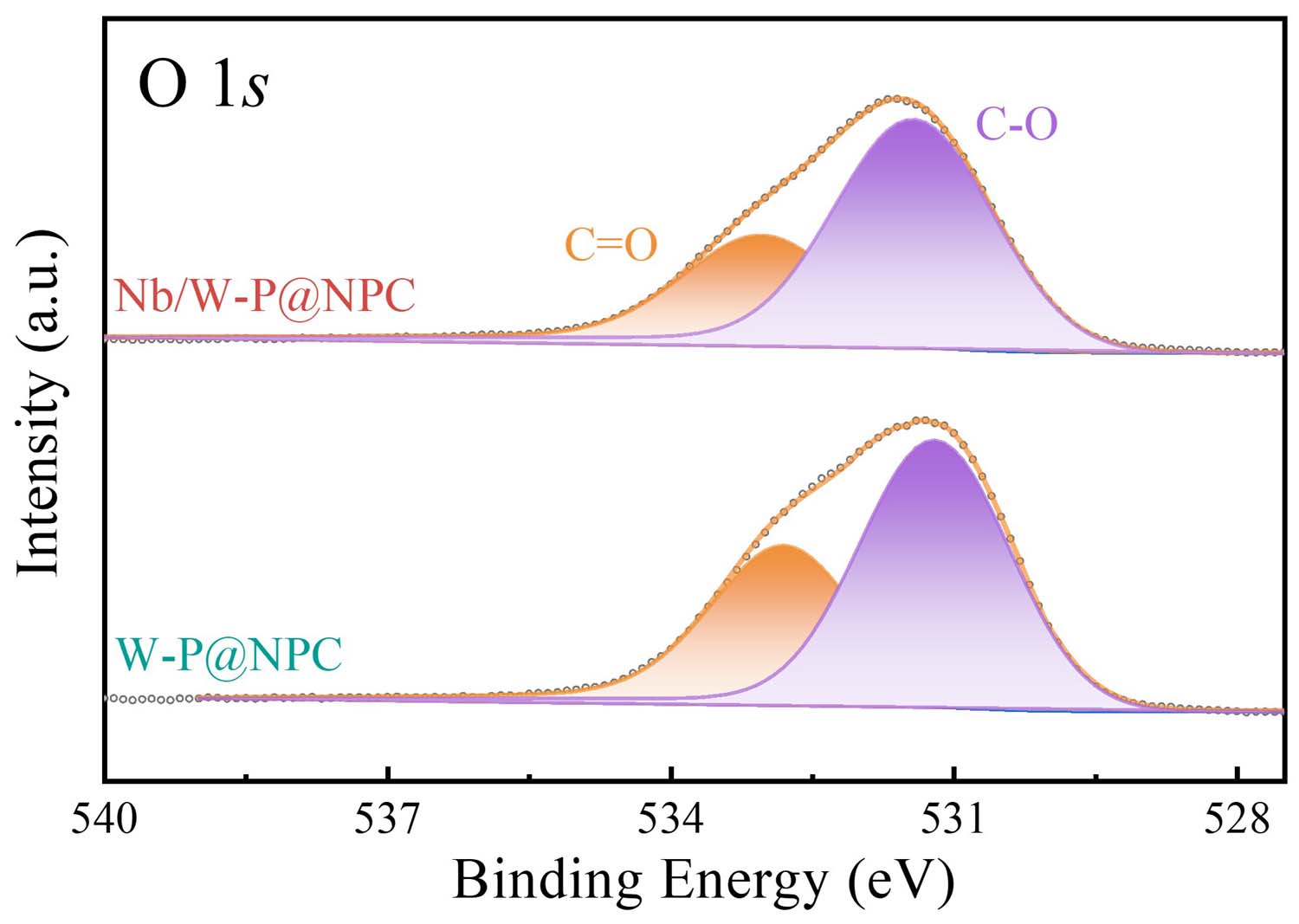


**Figure S17.** HR-XPS spectra of O 1*s* in W-P@NPC and Nb/W-P@NPC.

**
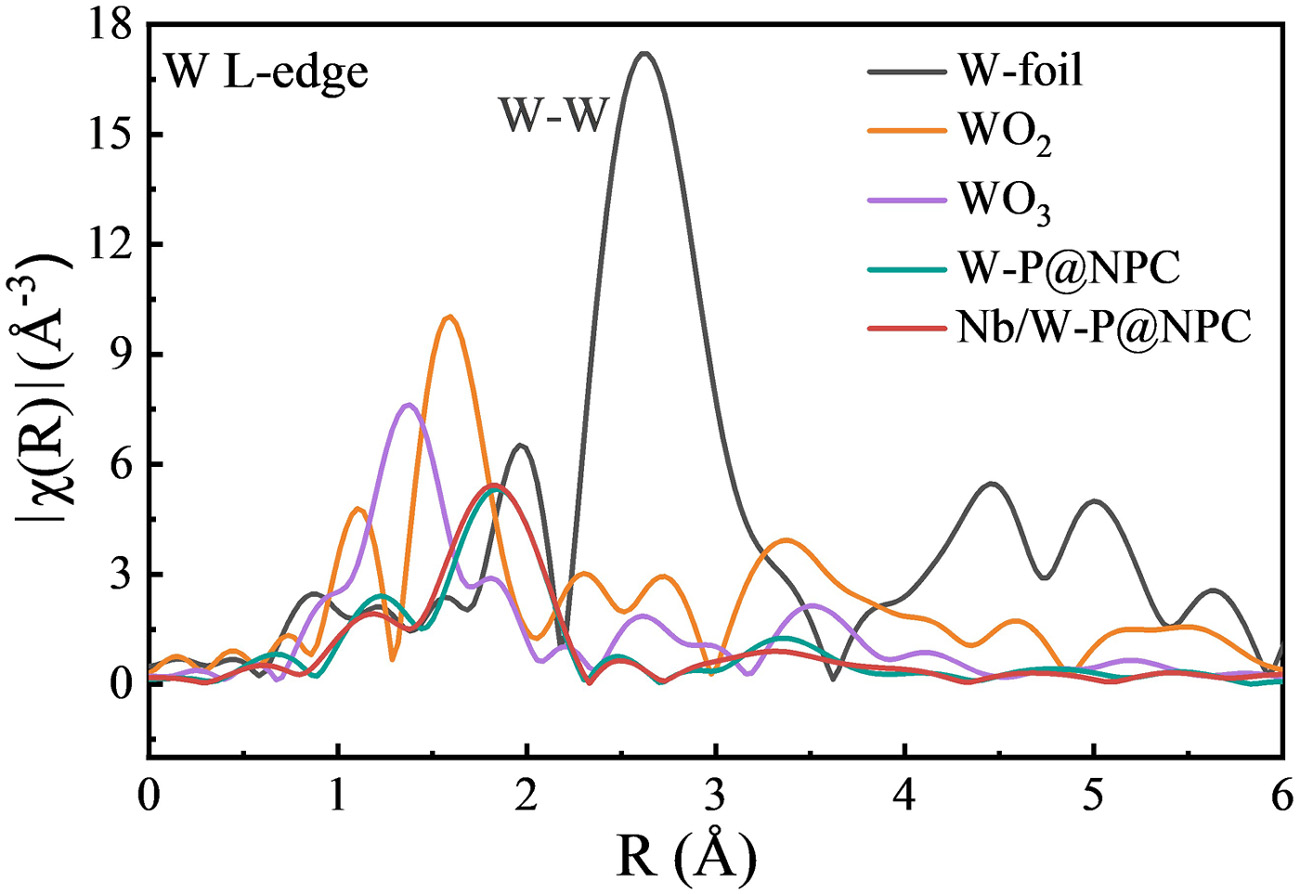
**

**Figure S18.** Normalized XANES spectra of W L-edge in W-P@NPC, Nb/W-P@NPC and reference samples.

**
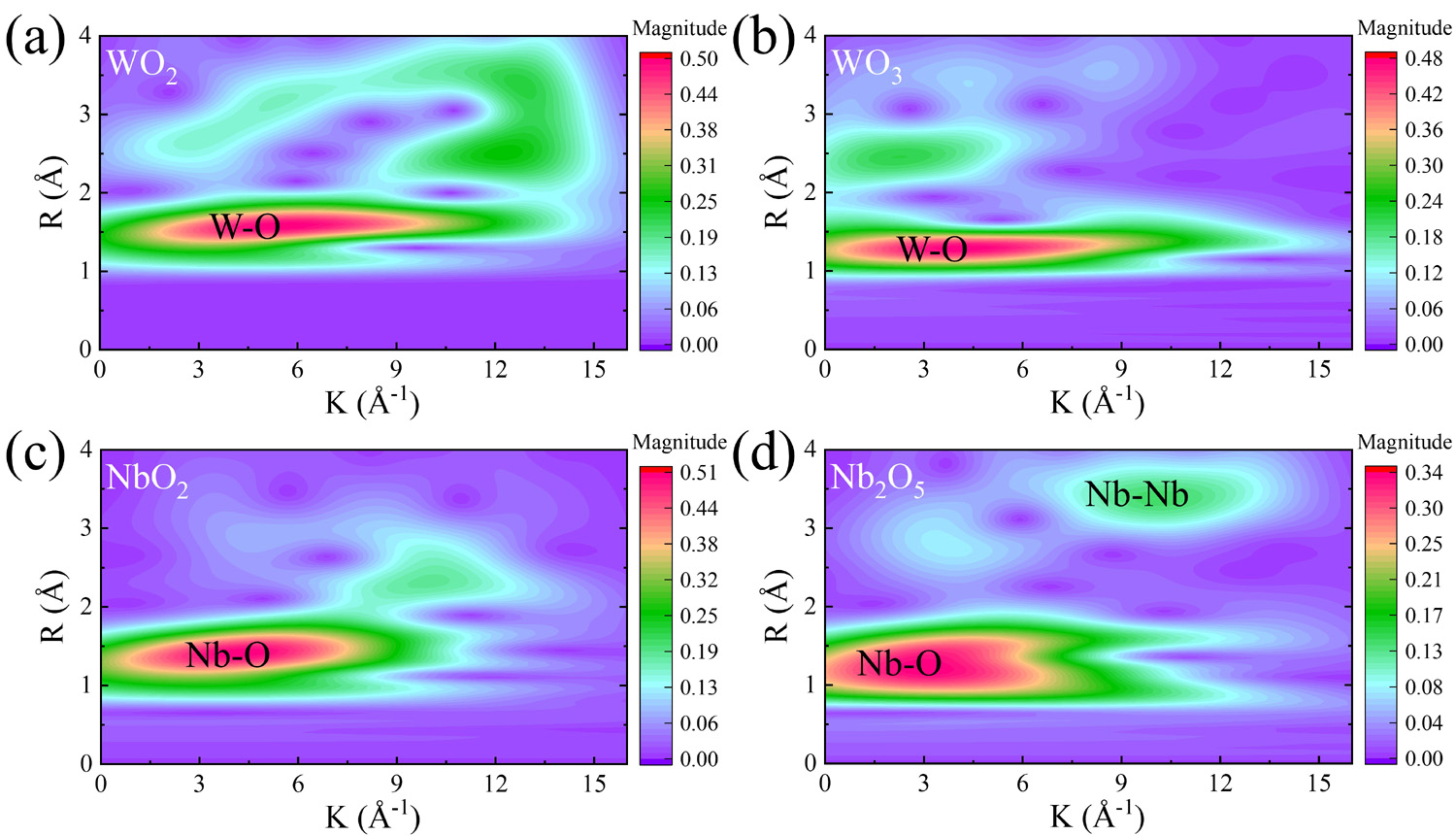
**

**Figure S19.** WT-EXAFS contour plots of (a) WO_2_, (b) WO_3_, (c) NbO_2_ and (d) Nb_2_O_5_.

**
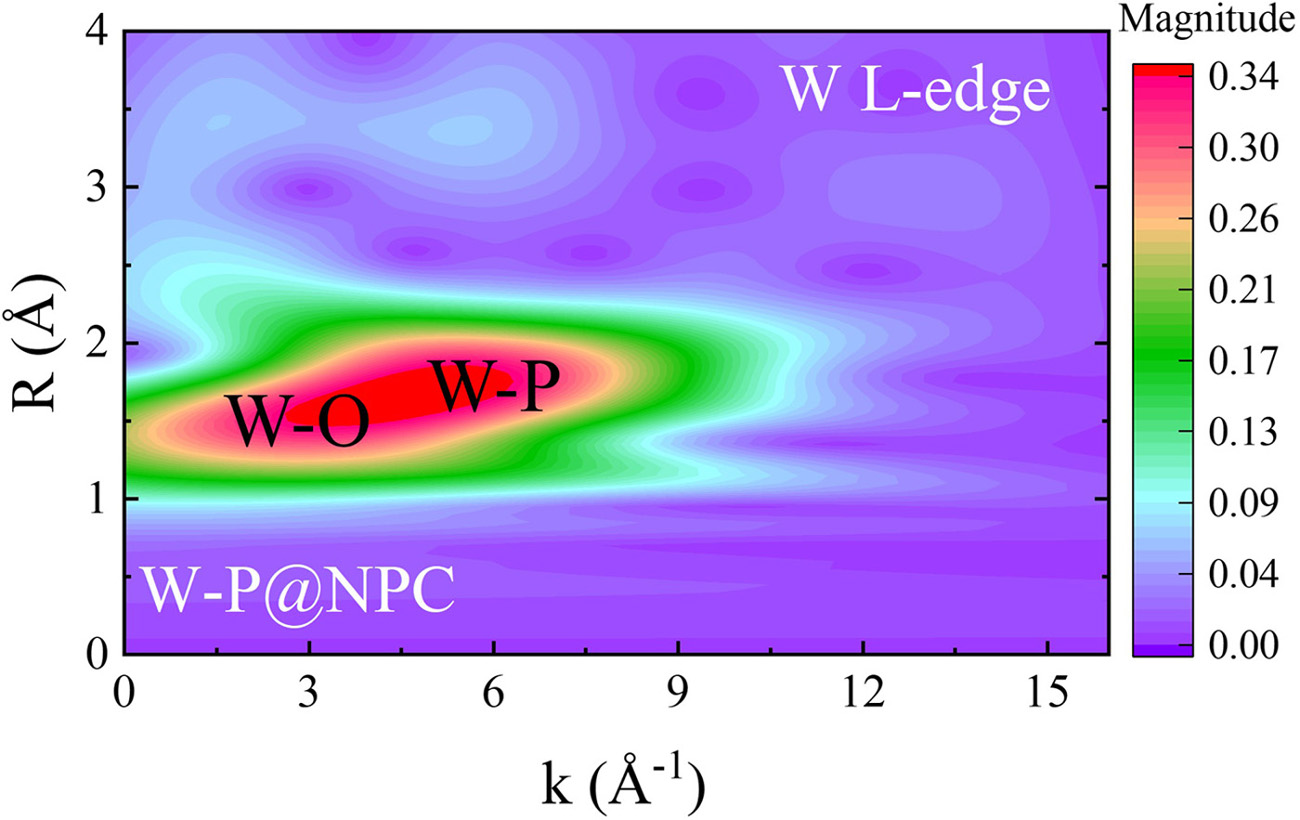
**

**Figure S20.** WT-EXAFS contour plot of W L-edge in W-P@NPC.

**
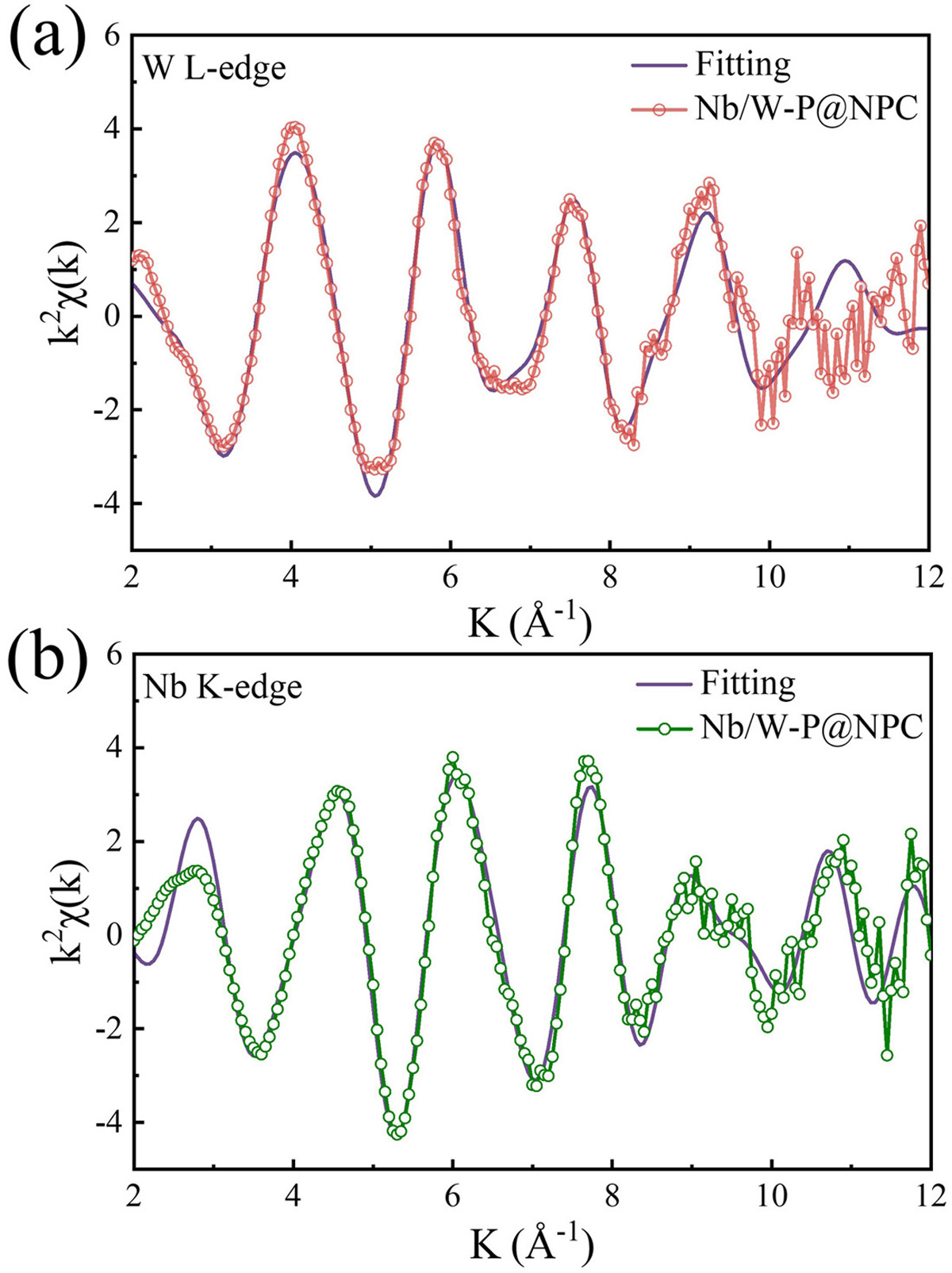
**

**Figure S21.** FT-EXAFS fittings in *K* space of (a) W L-edge and (b) Nb K-edge in Nb/W-P@NPC.

**
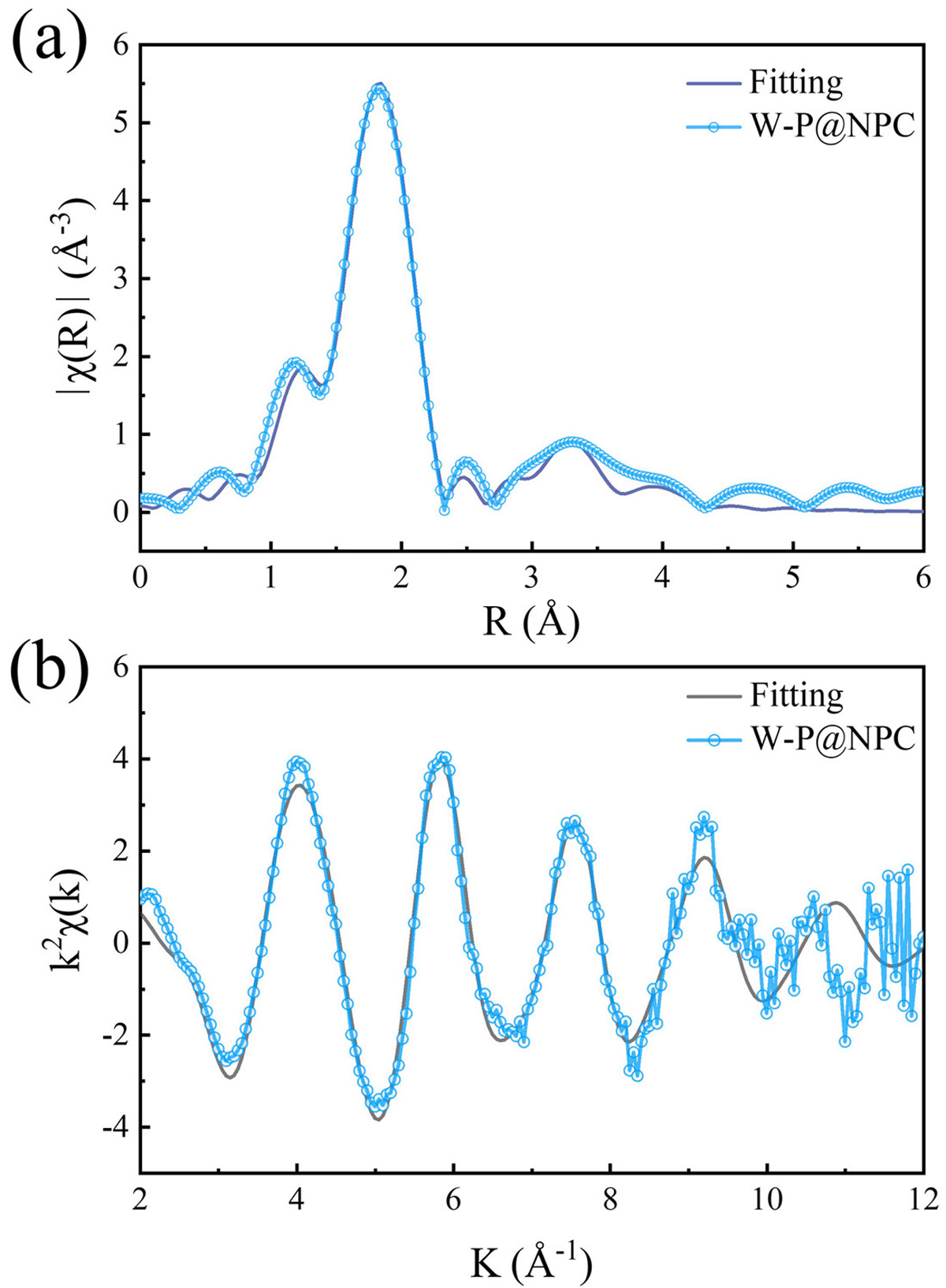
**

**Figure S22.** FT-EXAFS fittings in (a) *R* space and (b) *K* space of W-P@NPC.


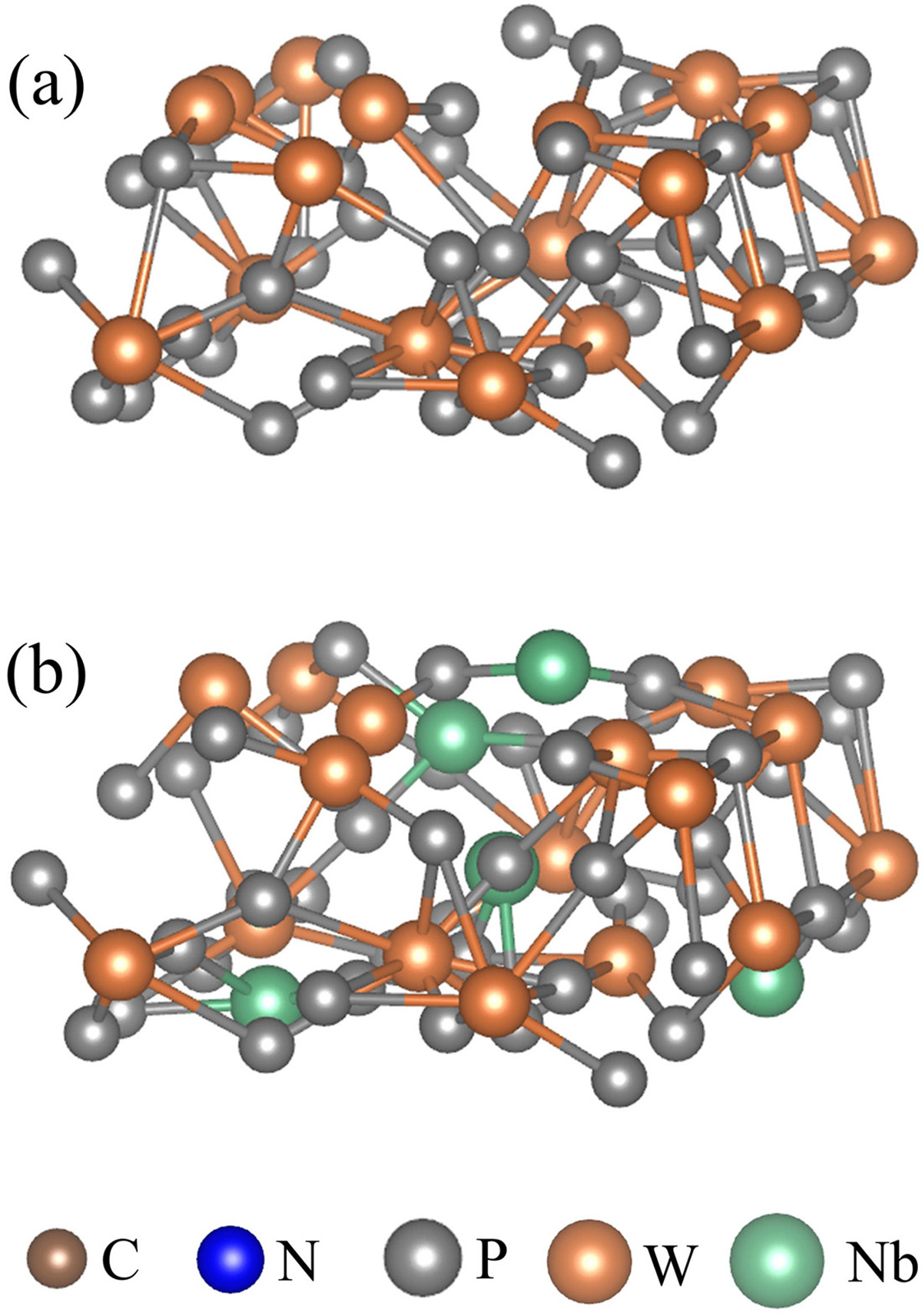


**Figure S23.** Theoretical modeling of amorphous W-P and Nb/W-P clusters.

**
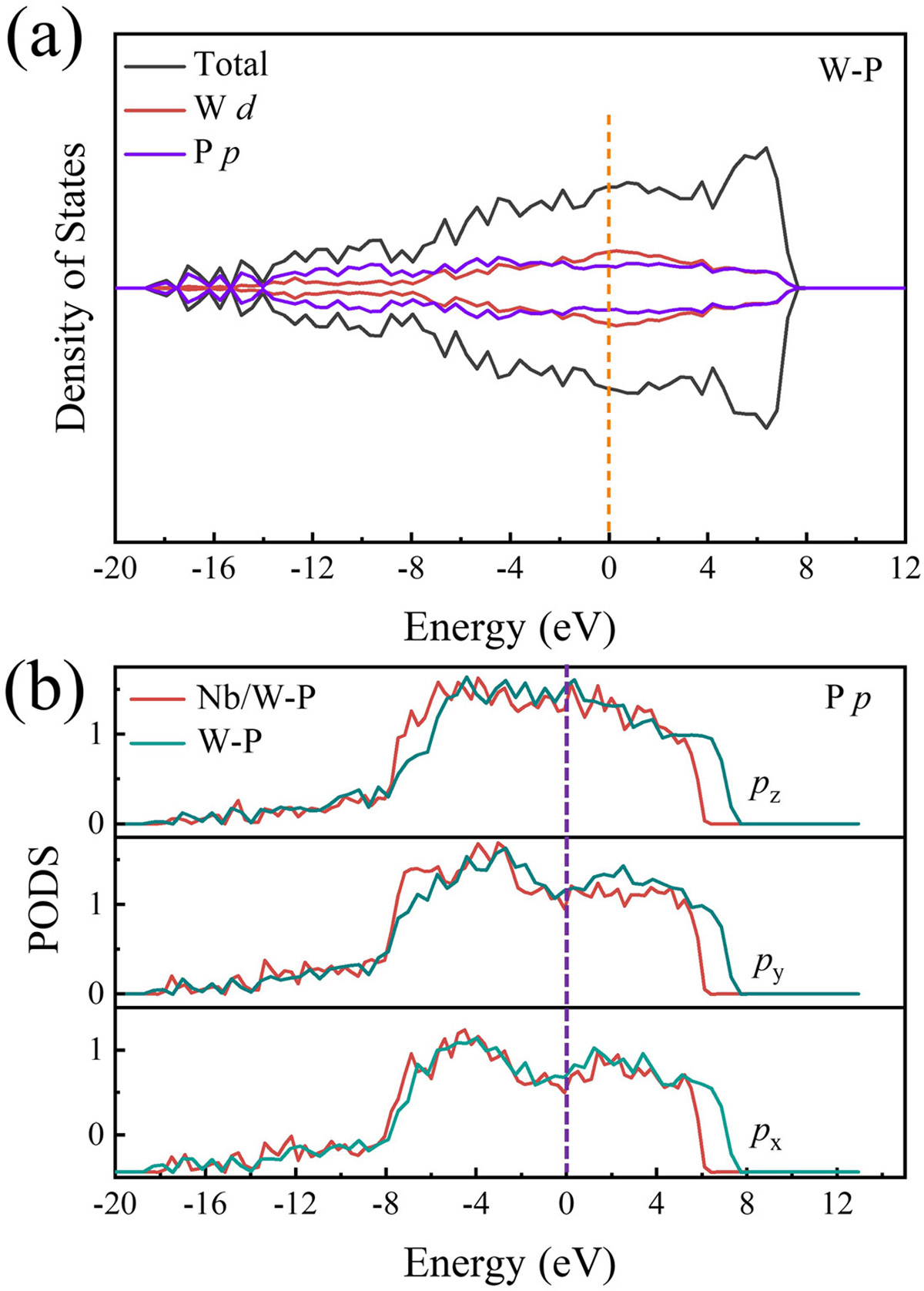
**

**Figure S24.** (a) Calculated DOS for W-P clusters and (b) *p* orbitals of P for W-P and Nb/W-P clusters in different dimensions.


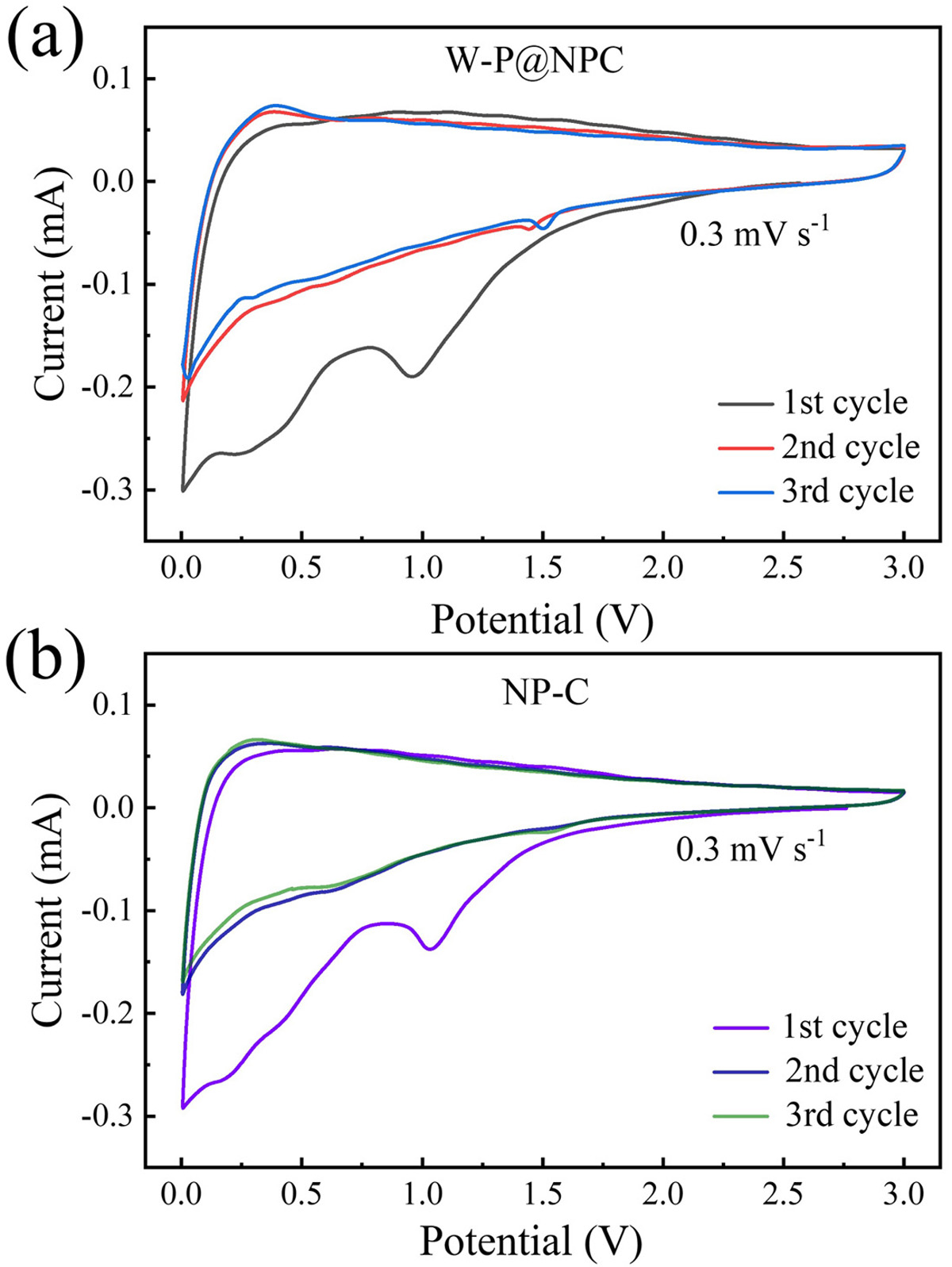


**Figure S25.** Initial three CV curves of (a) W-P@NPC and (b) NP-C at 0.3 mV s^-1^.

**
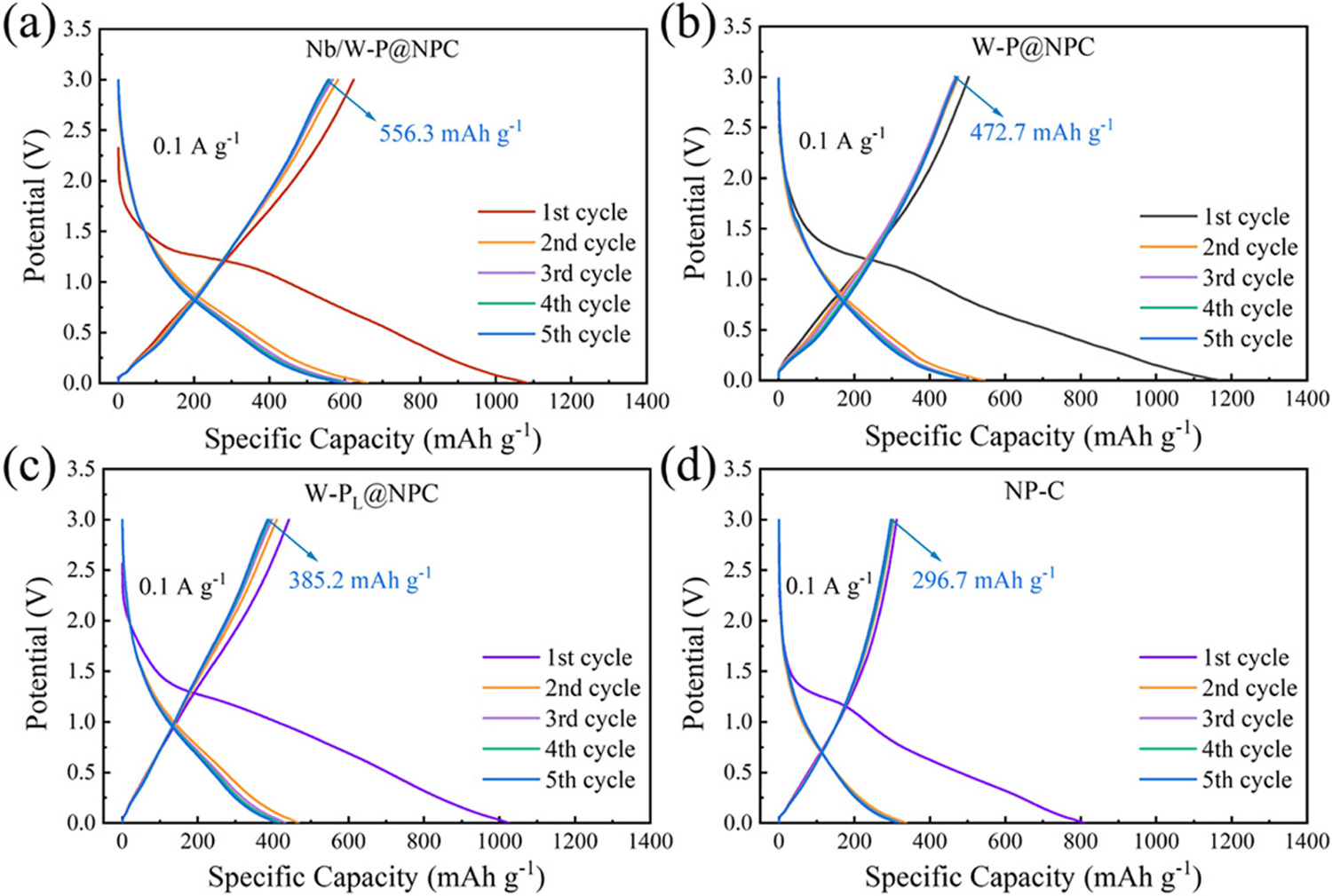
**

**Figure S26.** First five GCD curves of (a) Nb/W-P@NPC, (b) W-P@NPC, (c) W-P_L_@NPC and (d) NP-C at 0.1 A g^-1^.


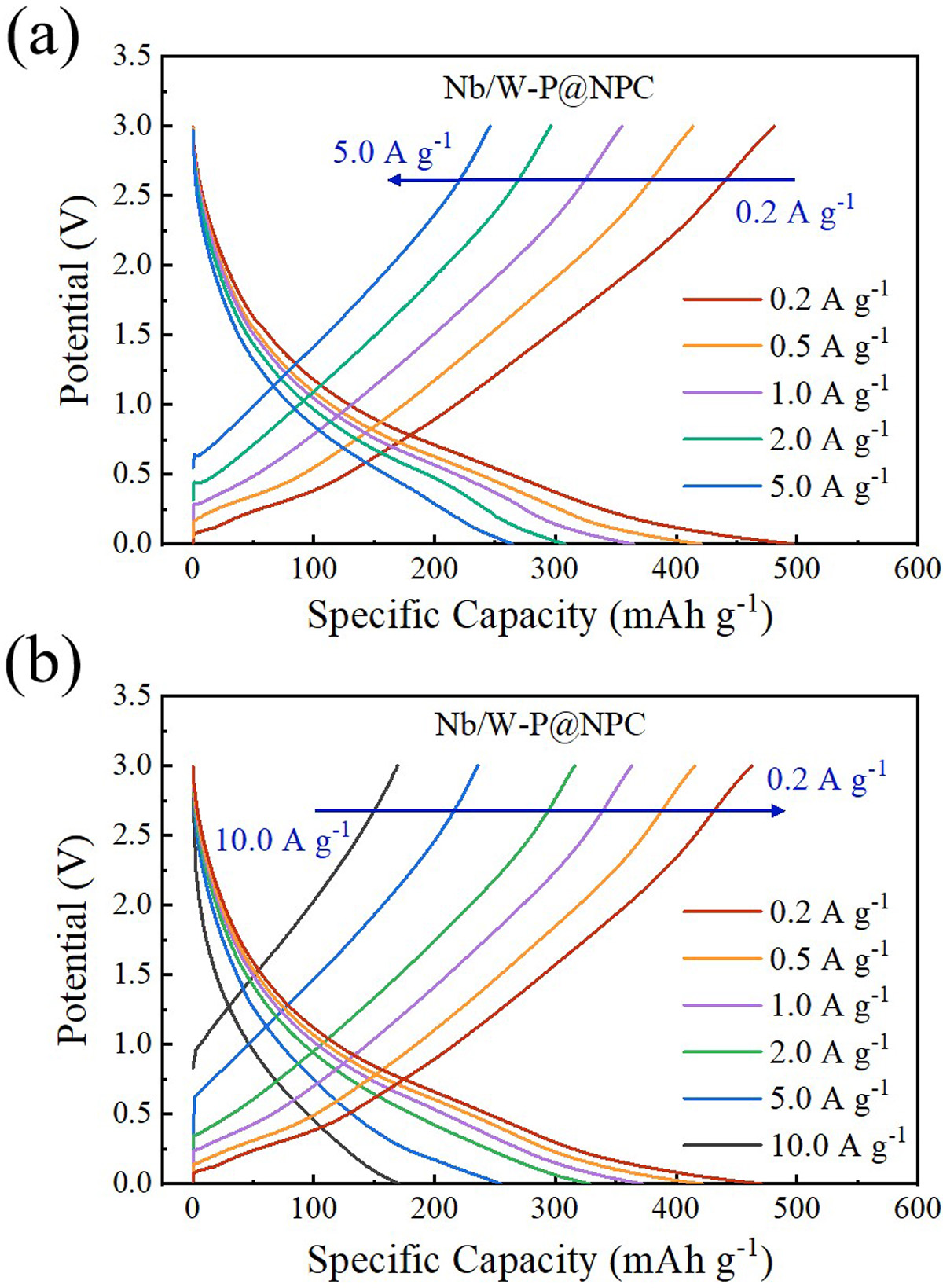


**Figure S27** Corresponding GCD curves of Nb/W-P@NPC at different current densities: (a) 0.2-5.0 A g^-1^ (increasing), (b) 10.0-0.2 A g^-1^ (decreasing).


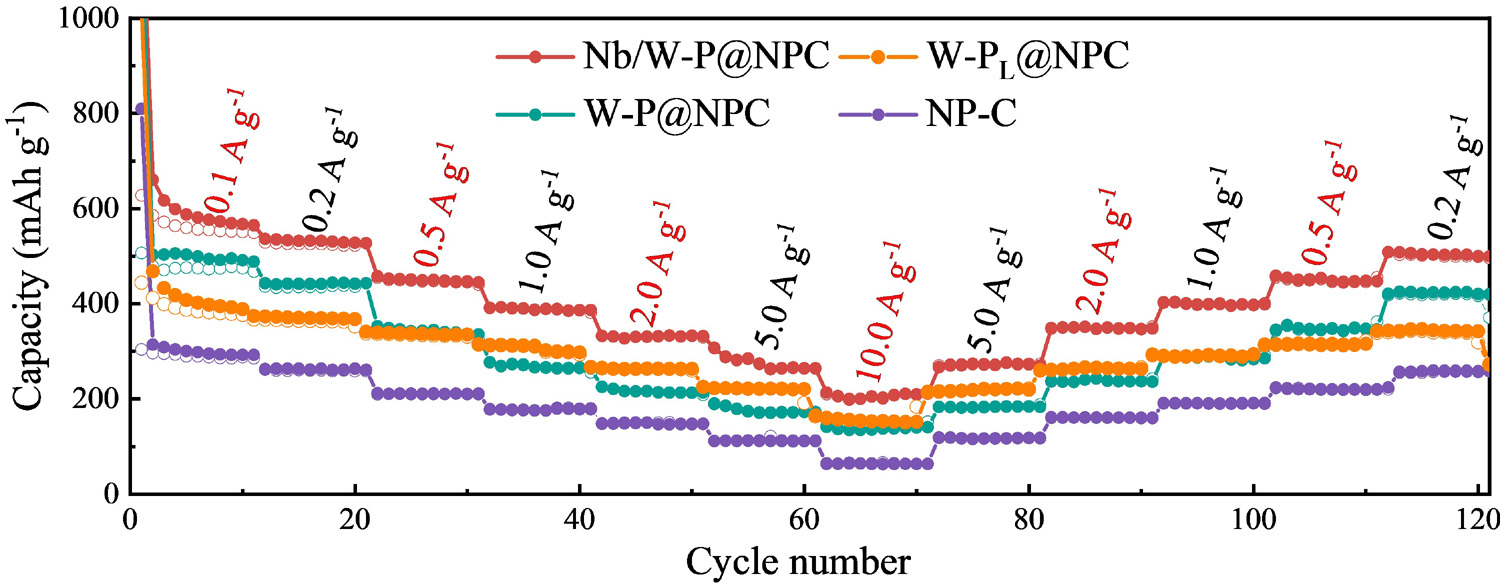


**Figure S28.** Rate capabilities of Nb/W-P@NPC, W-P@NPC, W-P_L_@NPC and NP-C.

**
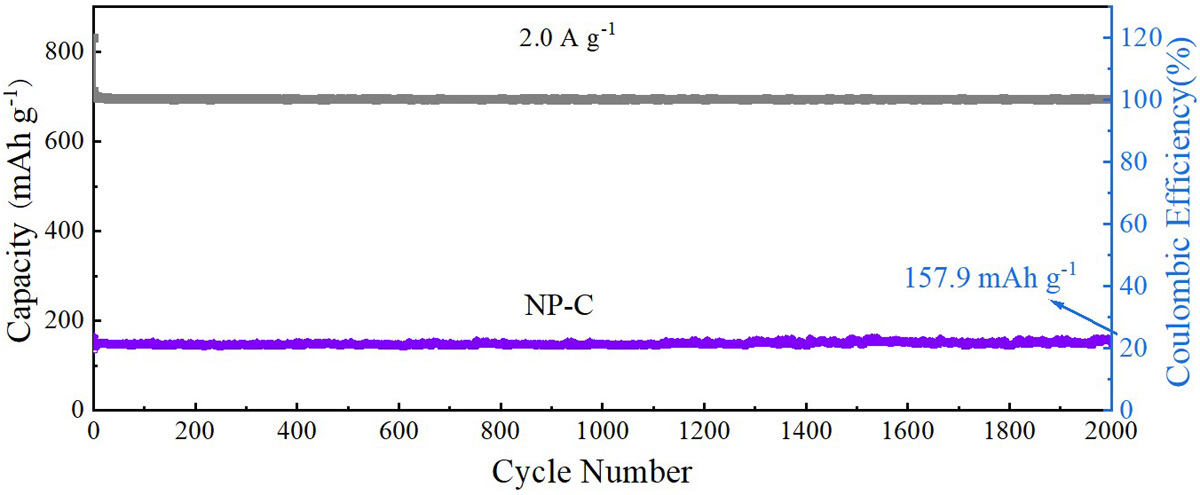
**

**Figure S29.** Long cycling stability of NP-C at 2.0 A g^-1^.

**
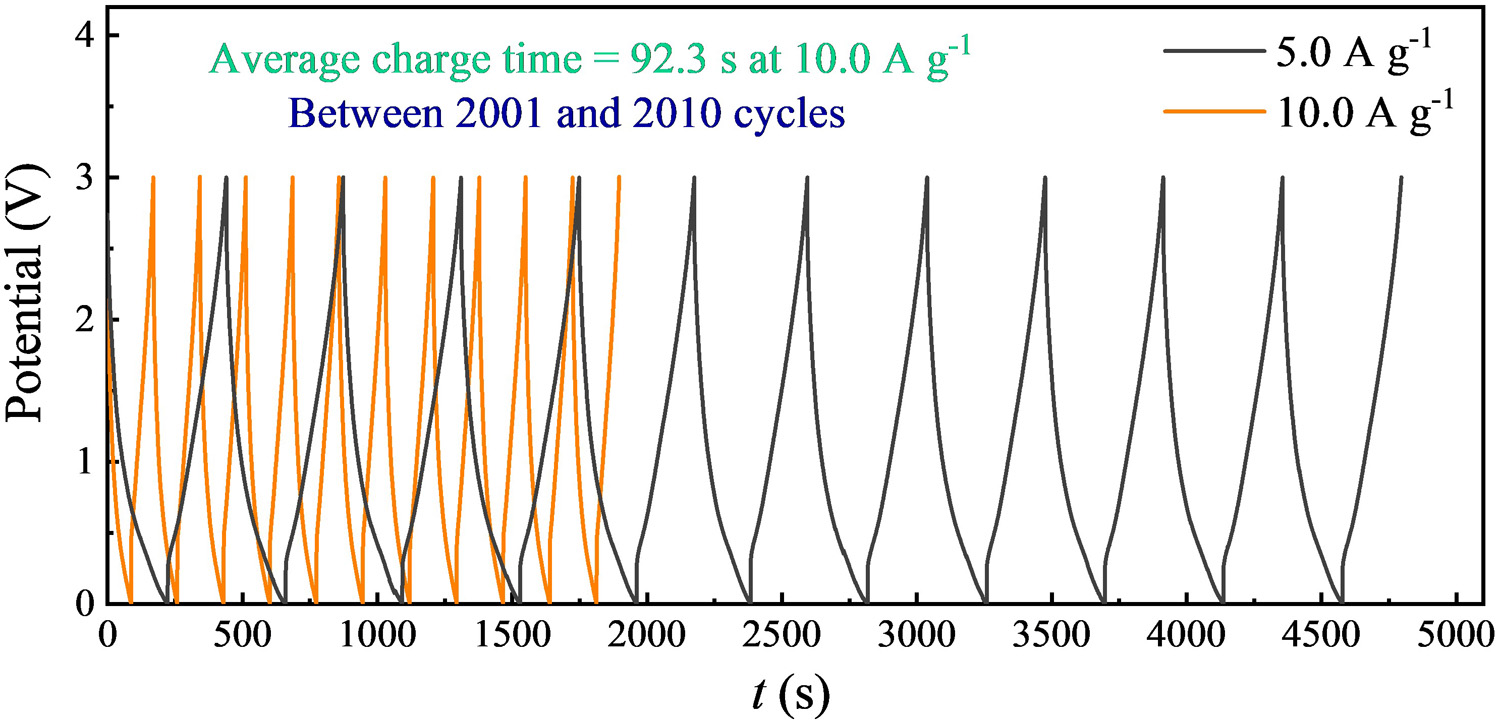
**

**Figure S30.** GCD curves of Nb/W-P@NPC at 5.0 and 10.0 A g^-1^.

**
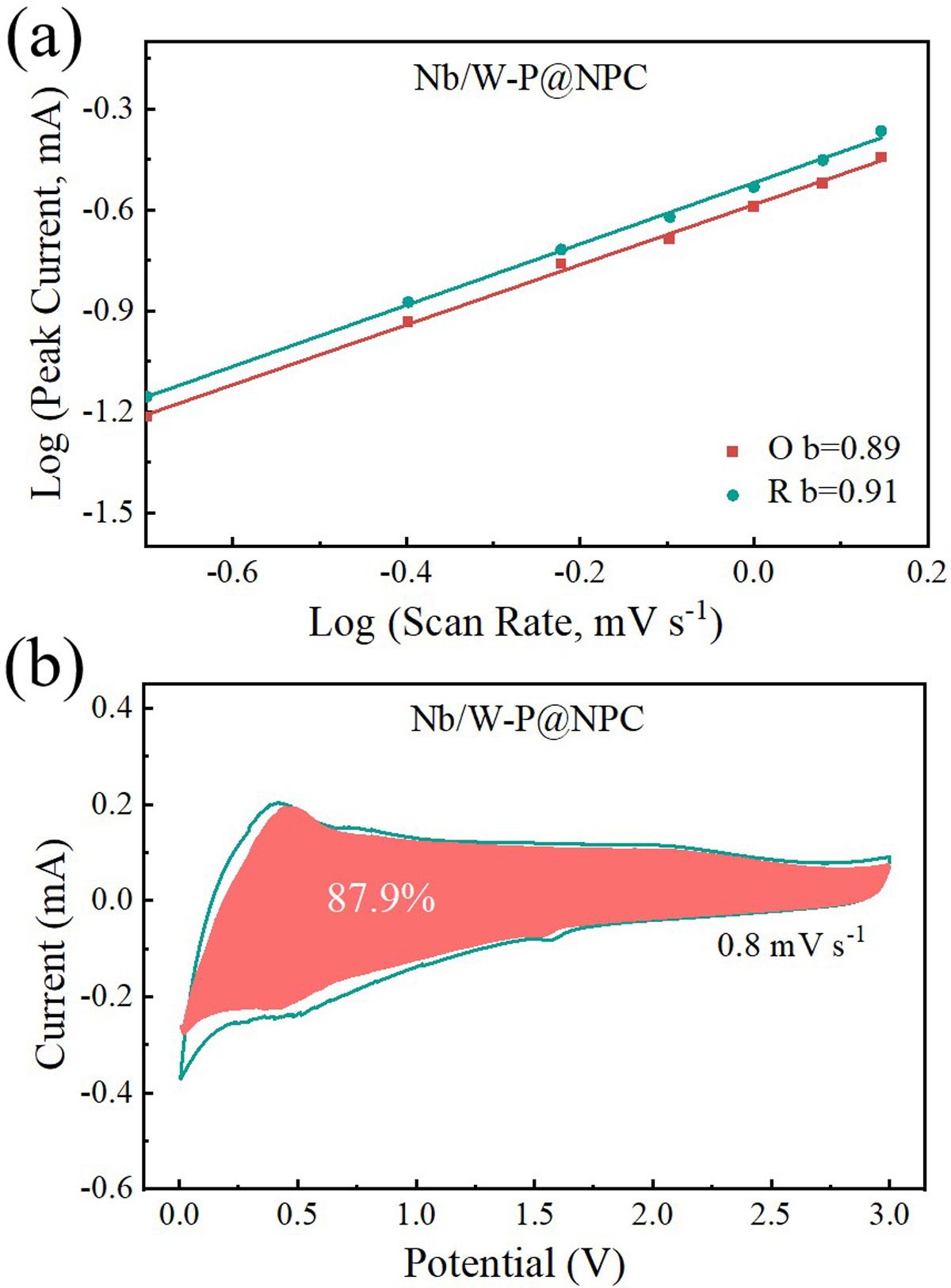
**

**Figure S31.** Kinetics analyses of Nb/W-P@NPC for SIHCs (a) Linear plots of log *i* and log *v*, (b) Capacitive contribution (shaded area) at 0.8 mV s^-1^.

**
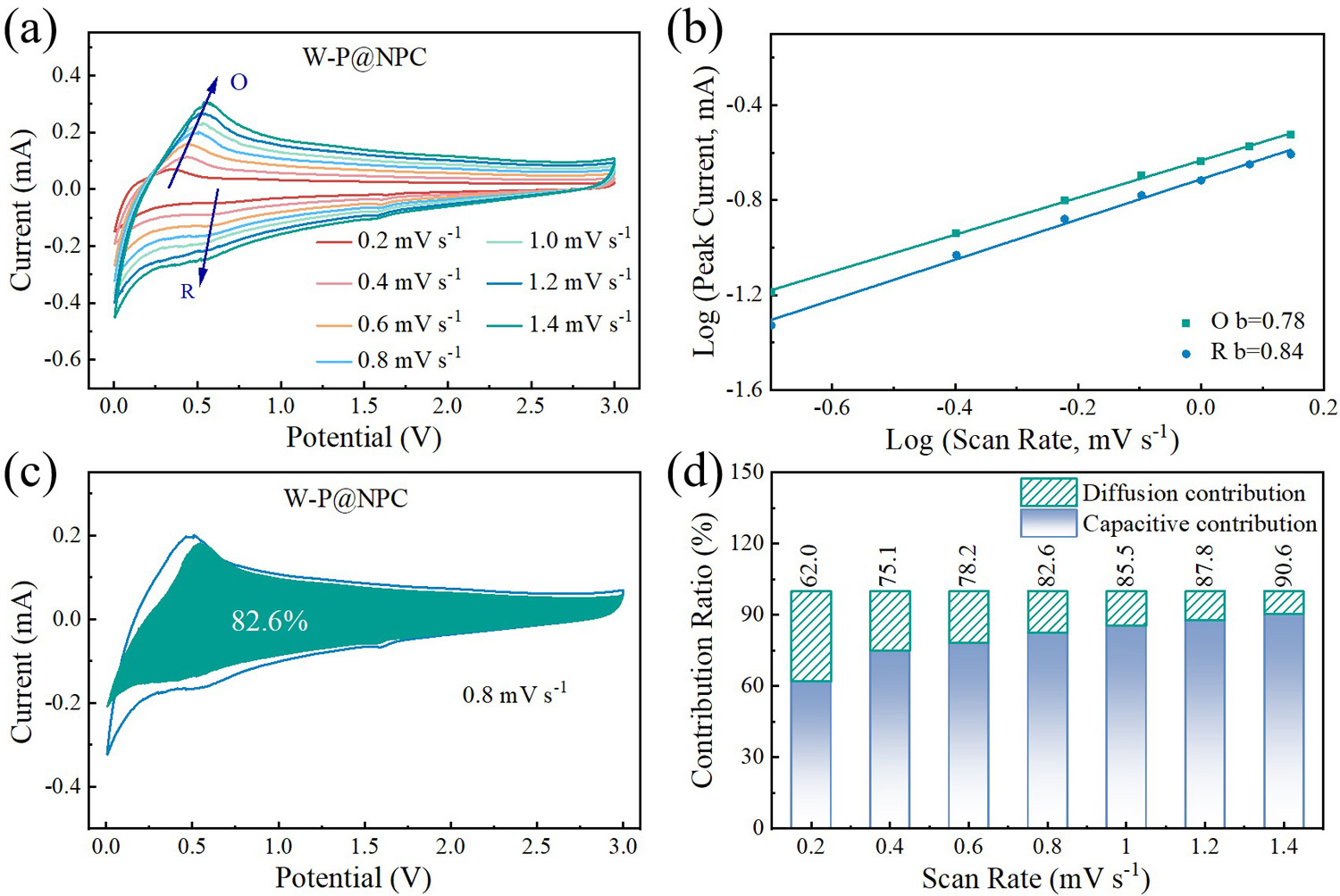
**

**Figure S32.** Kinetics analyses of W-P@NPC for SIHCs (a) CVs at various scan rates from 0.2 to 1.4 mV s^-1^, (b) Linear plots of log *i* and log *v*, (c) Capacitive contribution at 0.8 mV s^-1^; (d) Capacitive ratios at different scan rates.

**
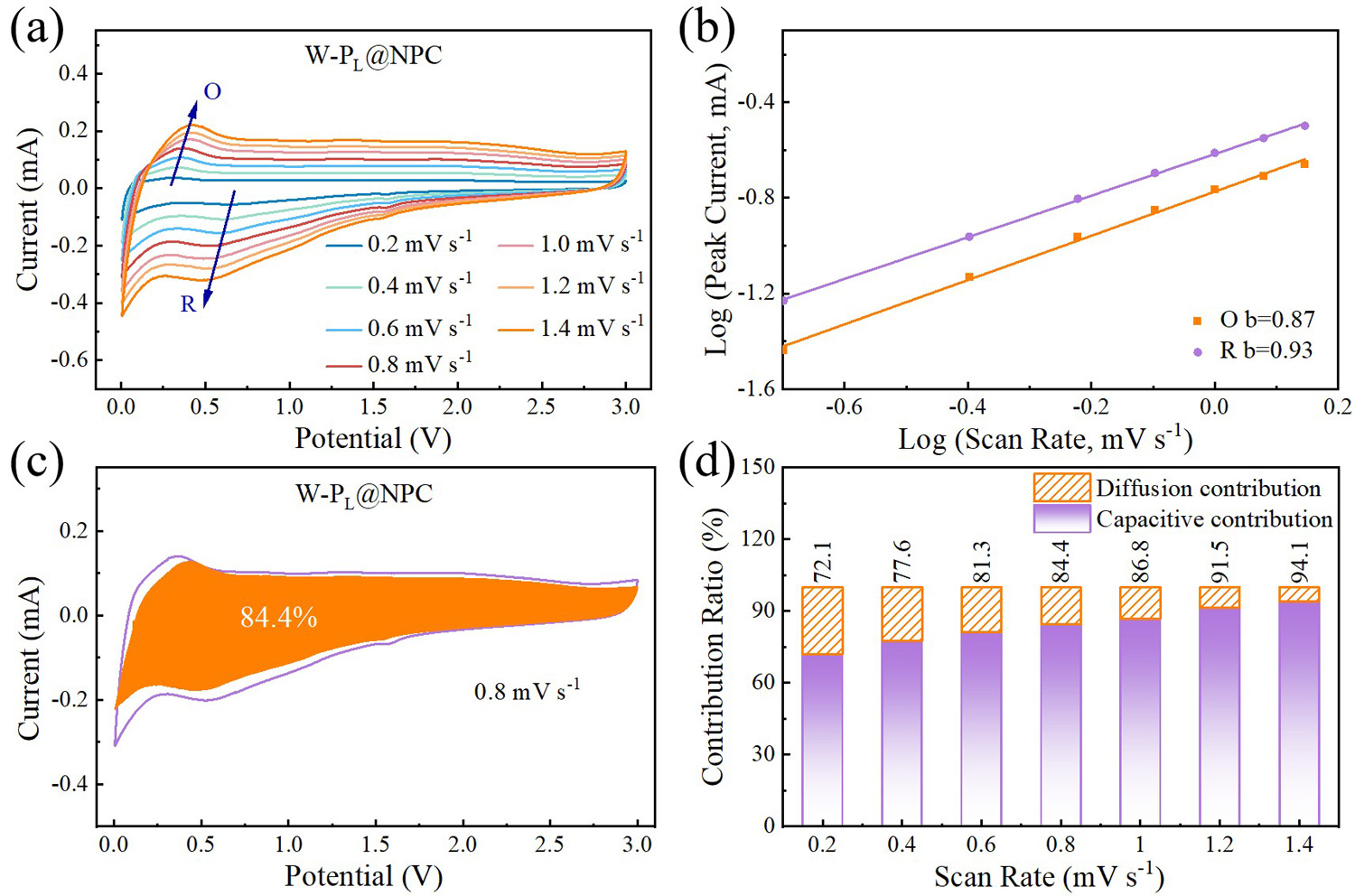
**

**Figure S33.** Kinetics analyses of W-P_L_@NPC for SIHCs (a) CVs at various scan rates from 0.2 to 1.4 mV s^-1^, (b) Linear plots of log *i* and log *v*, (c) Capacitive contribution at 0.8 mV s^-1^; (d) Capacitive ratios at different scan rates.


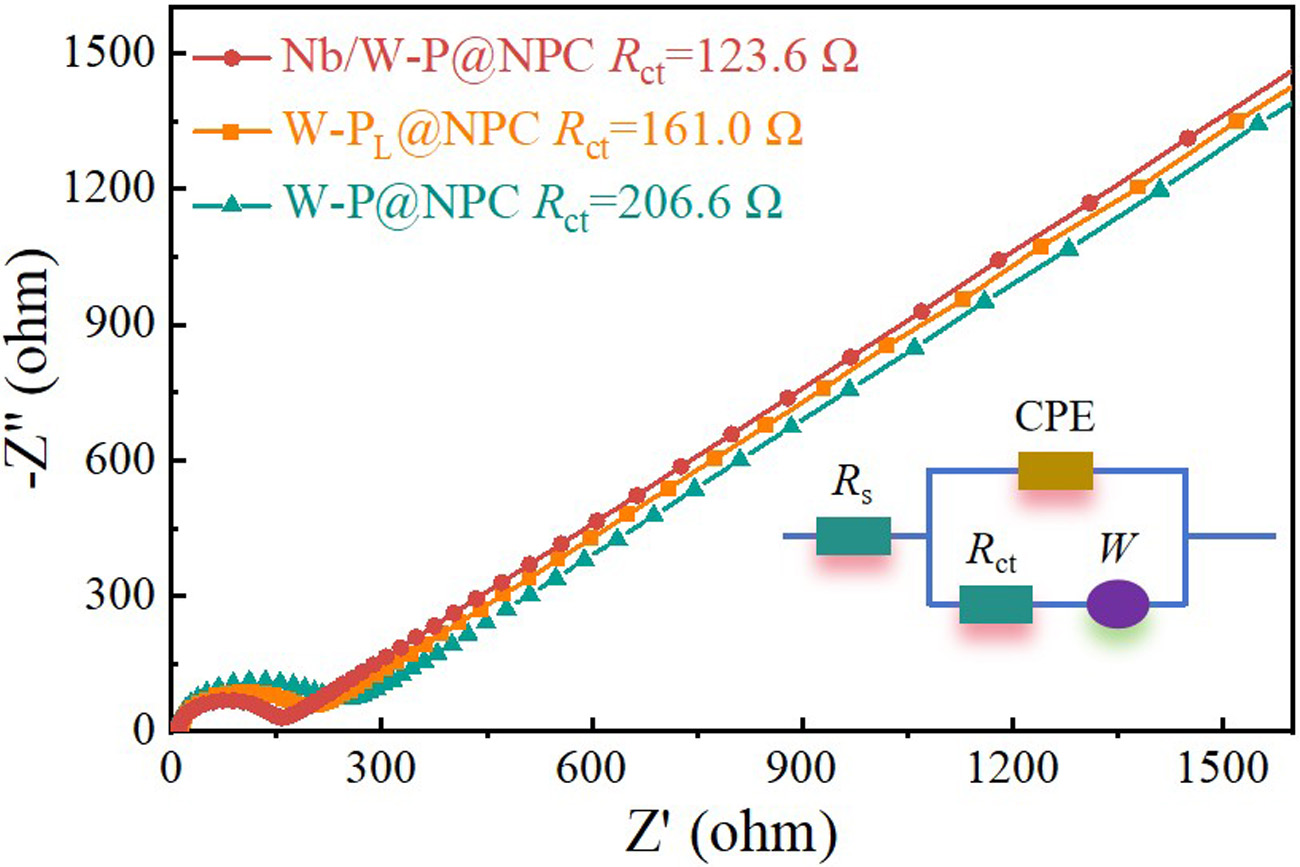


**Figure S34.** Nyquist plots of Nb/W-P@NPC, W-P@NPC and W-P_L_@NPC with an equivalent circuit (inset).


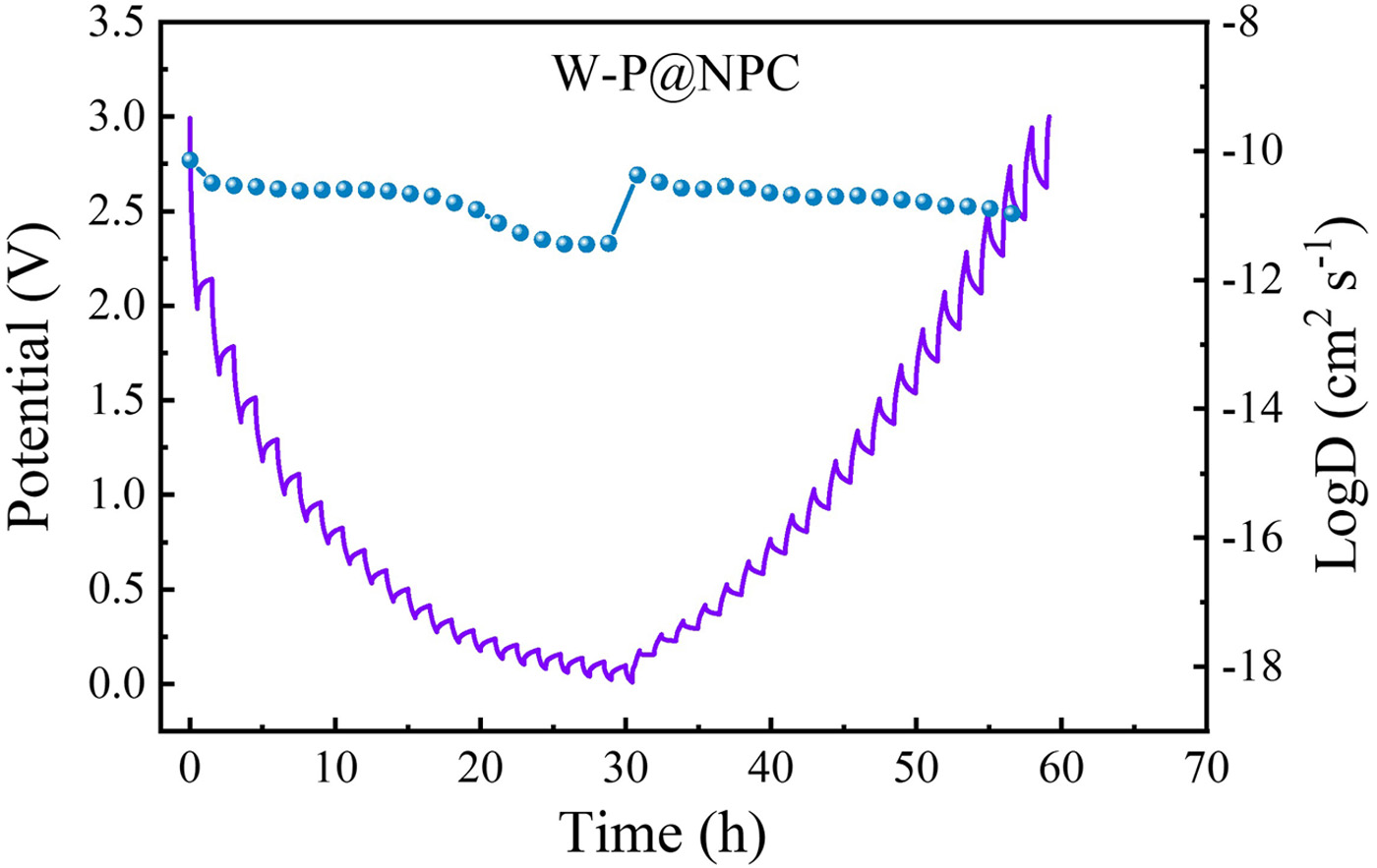


**Figure S35.** GITT profile with diffusion coefficients of Na^+^ for W-P@NPC during cycling.


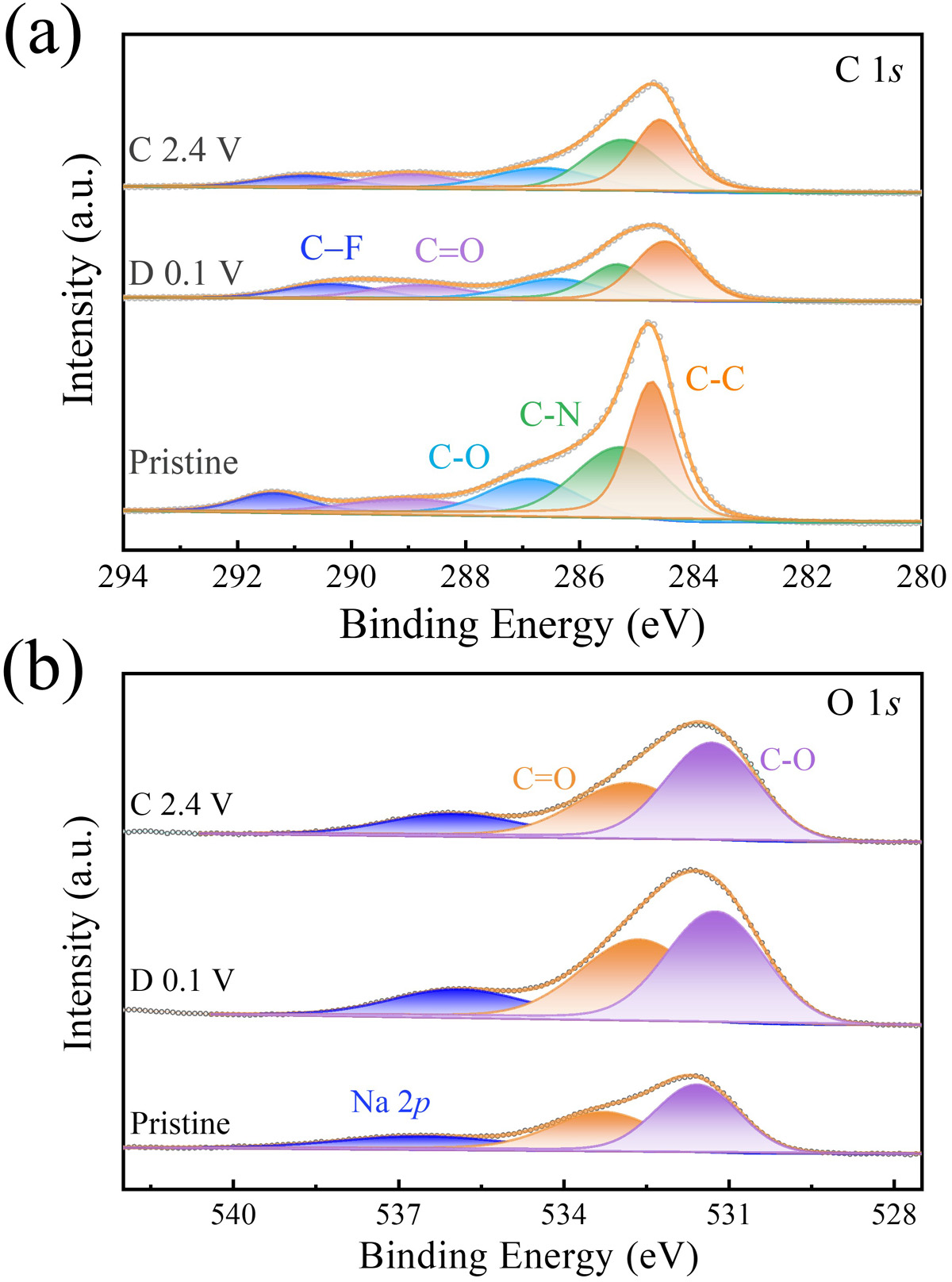


**Figure S36.** *Ex-situ* HR-XPS spectra of (a) C 1*s* and (b) O 1*s* for Nb/W-P@NPC at different sodiation states.


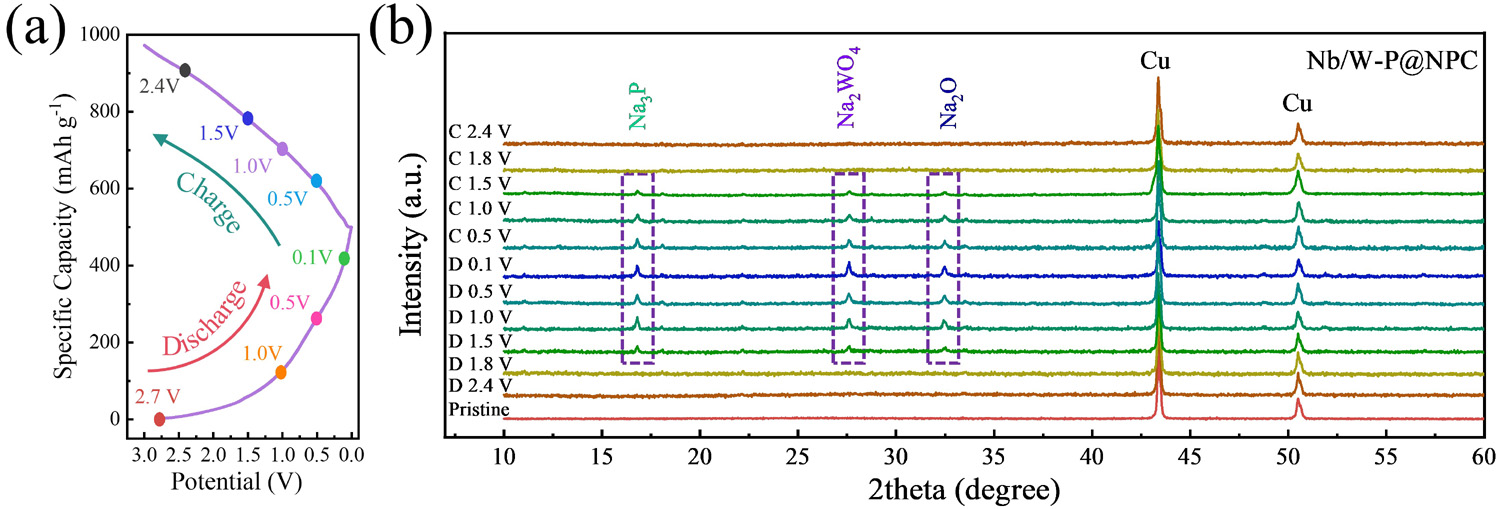


**Figure S37.** (a) GCD curve and (b) corresponding *ex-situ* XRD patterns of Nb/W-P@NPC at different sodiation states.


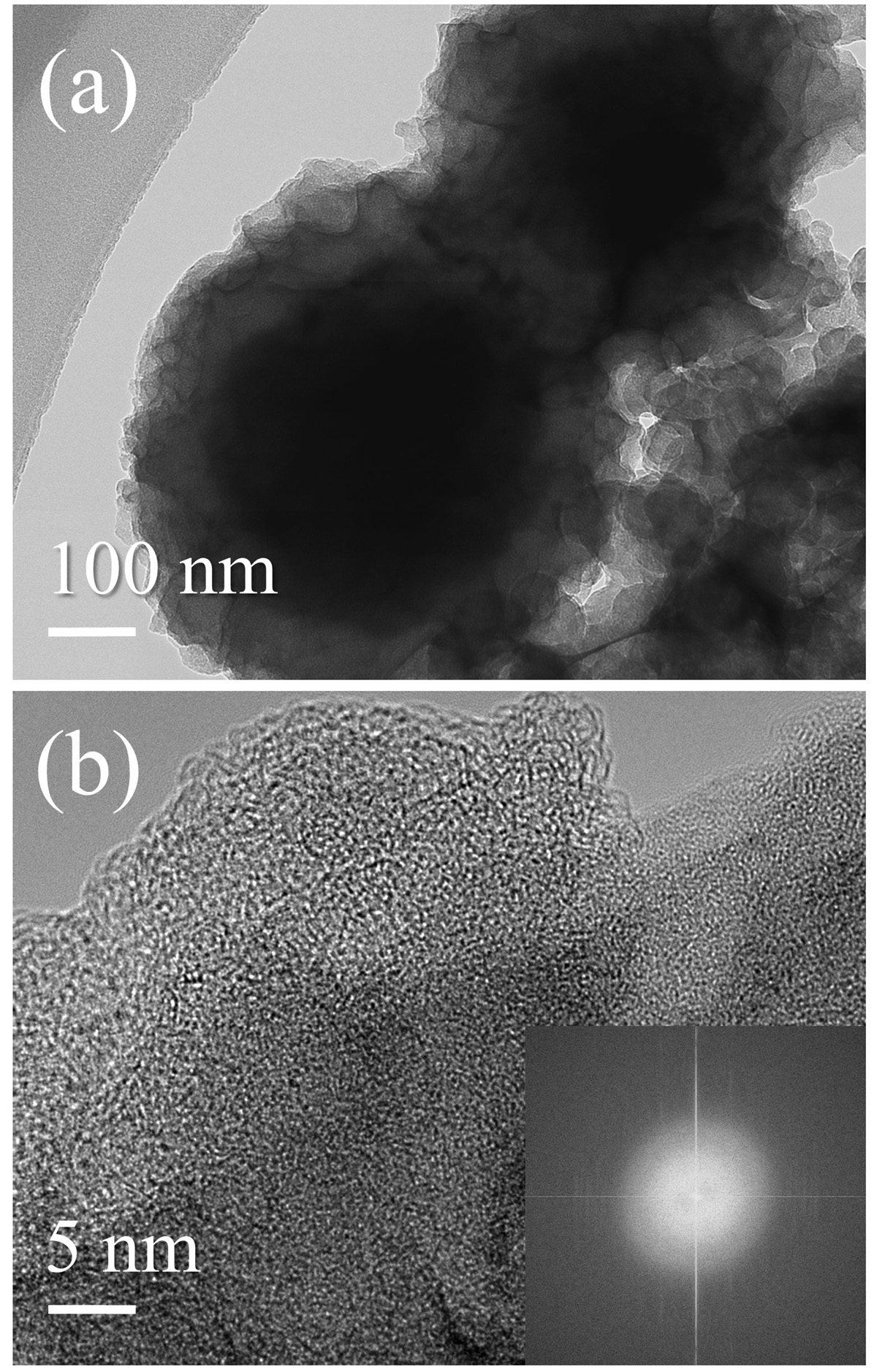


**Figure S38.** *Ex-situ* (a) TEM image and (b) HR-TEM image with FFT image (inset) of Nb/W-P@NPC after 50 cycles and discharging to 0.1 V.


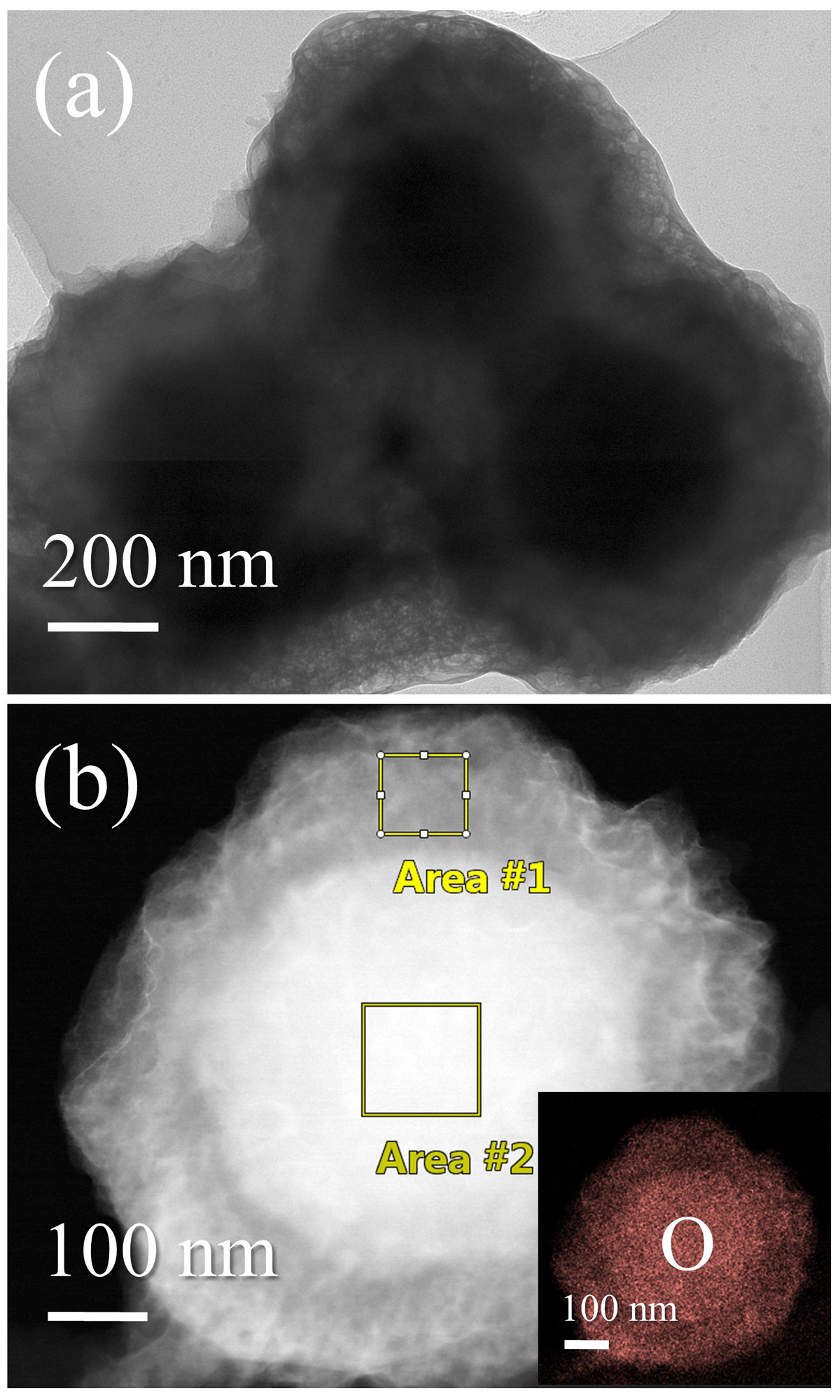


**Figure S39.** *Ex-situ* (a) TEM image and (b) HAADF-STEM image of Nb/W-P@NPC after 2000 cycles and discharged to 0.1 V (elemental mapping of O, inset).


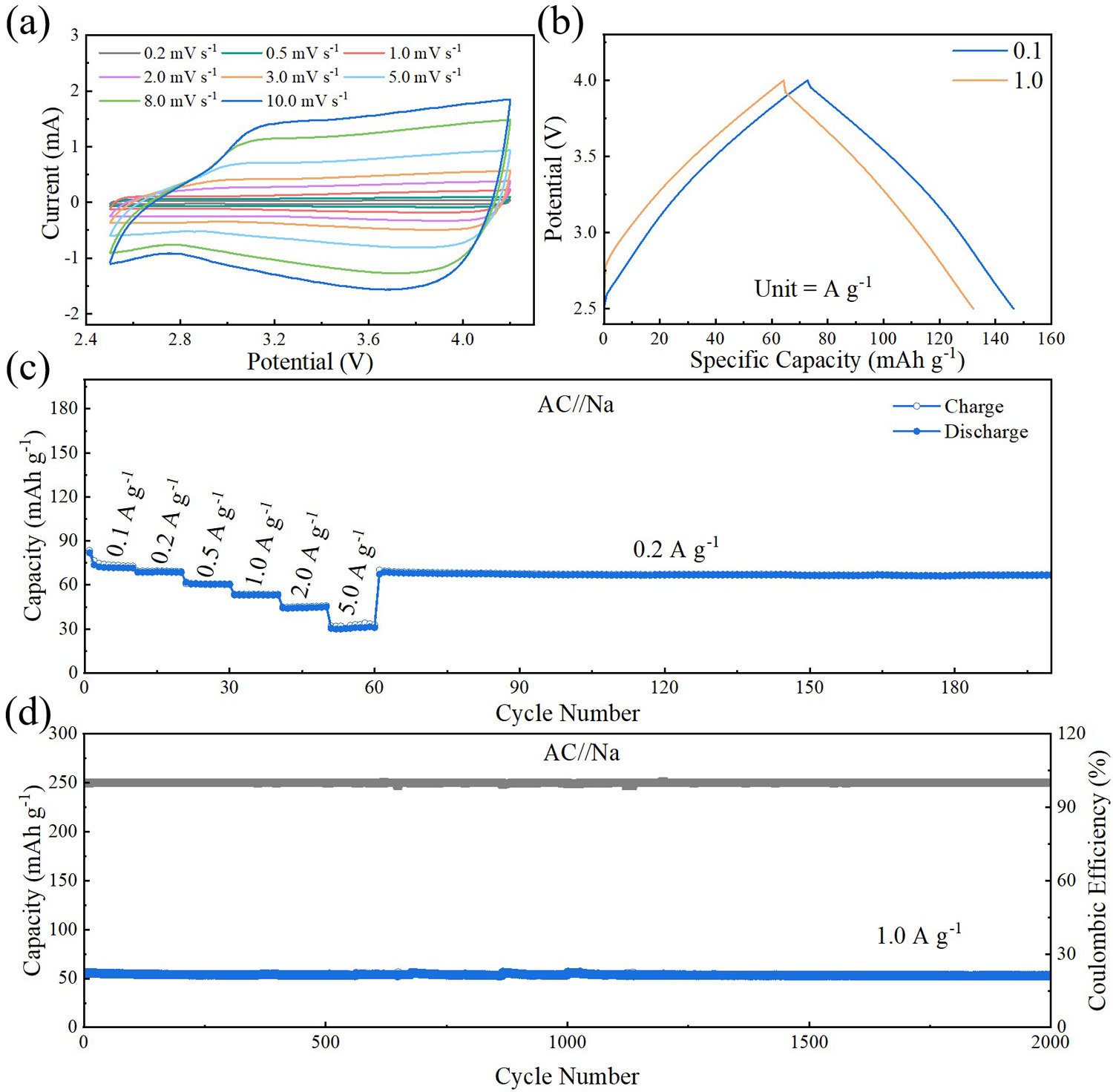


**Figure S40.** Electrochemical properties of the AC cathode: (a) CV curves at different scan rates, (b) GCD curves, (c) Rate capability, and (d) Long cycling stability at 1.0 A g^-1^.


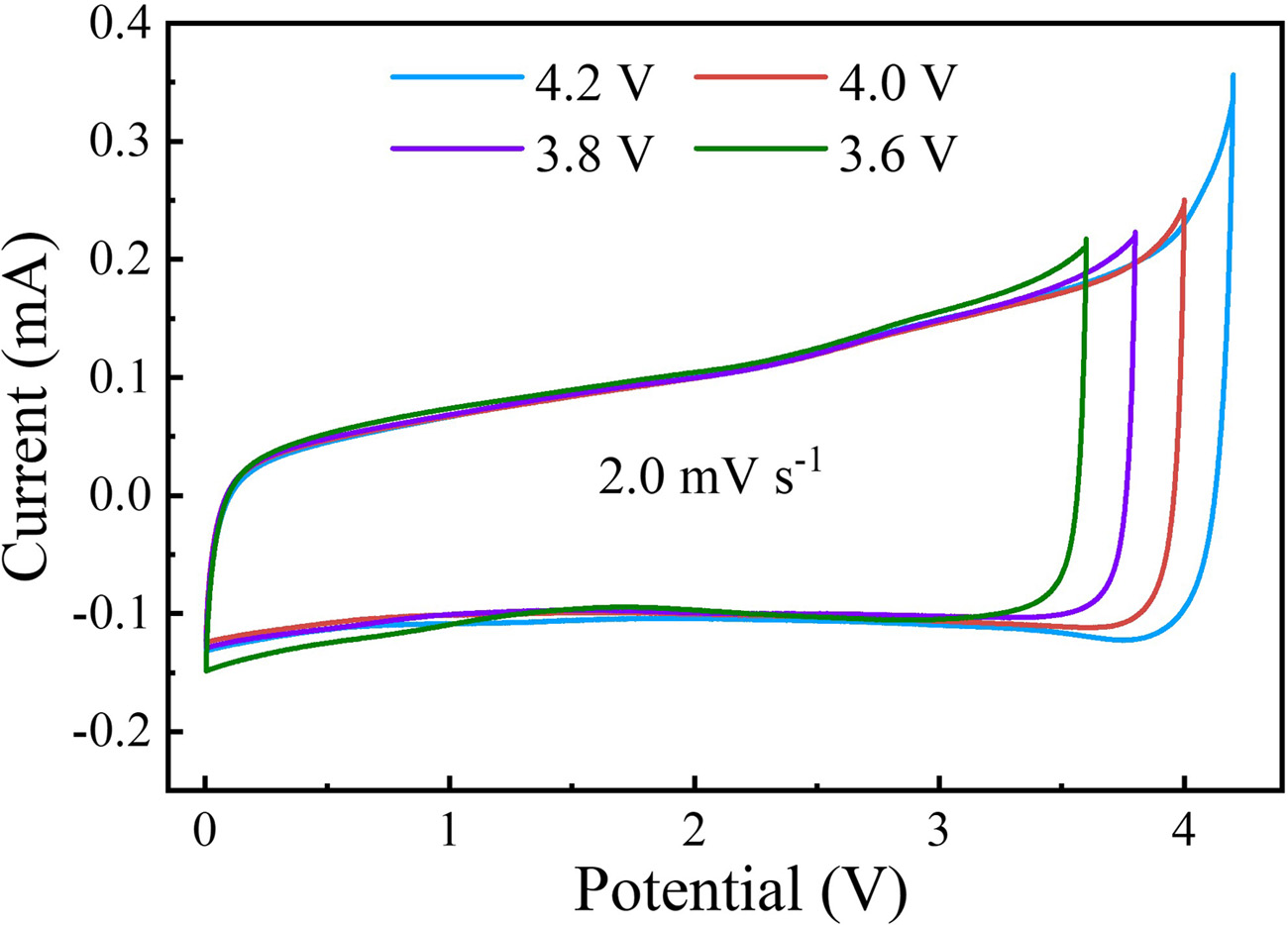


**Figure S41.** CVs of Nb/W-P@NPC//AC SIHCs at various potential ranges.


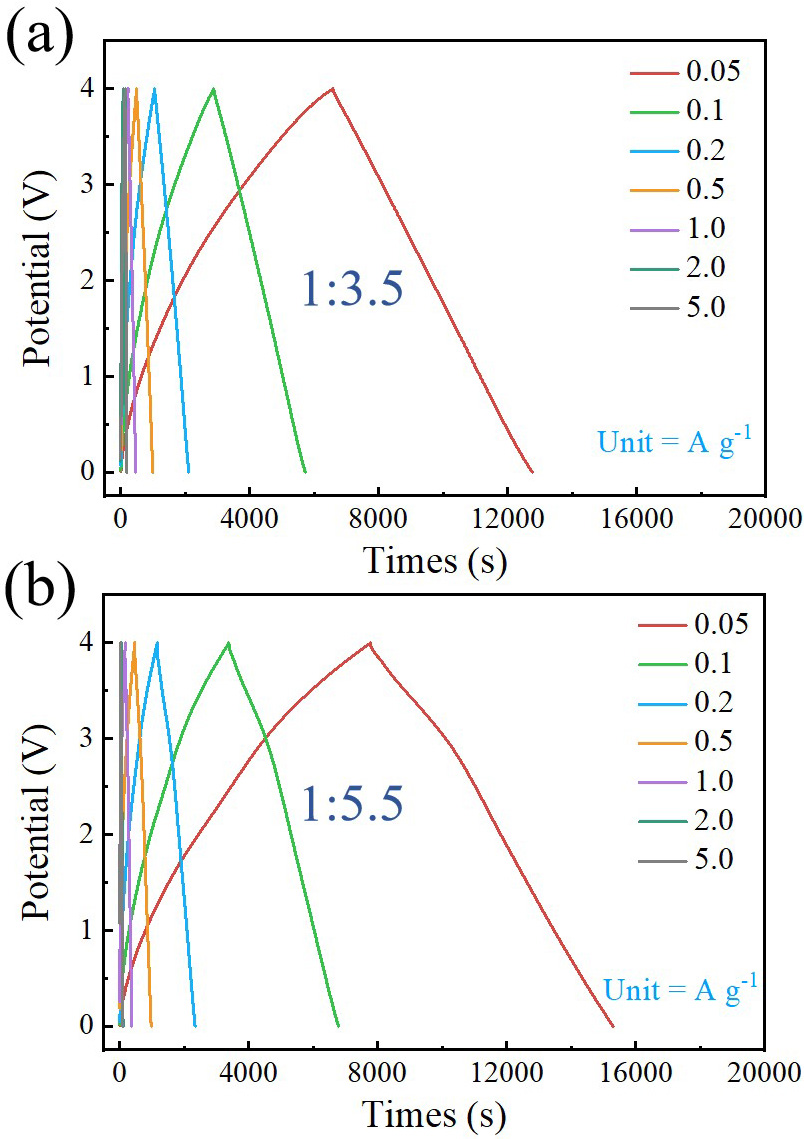


**Figure S42.** GCD curves of Nb/W-P@NPC//AC SIHCs with anode-to-cathode mass ratios of (a) 1:3.5 and (b) 1:5.5 at various current densities.


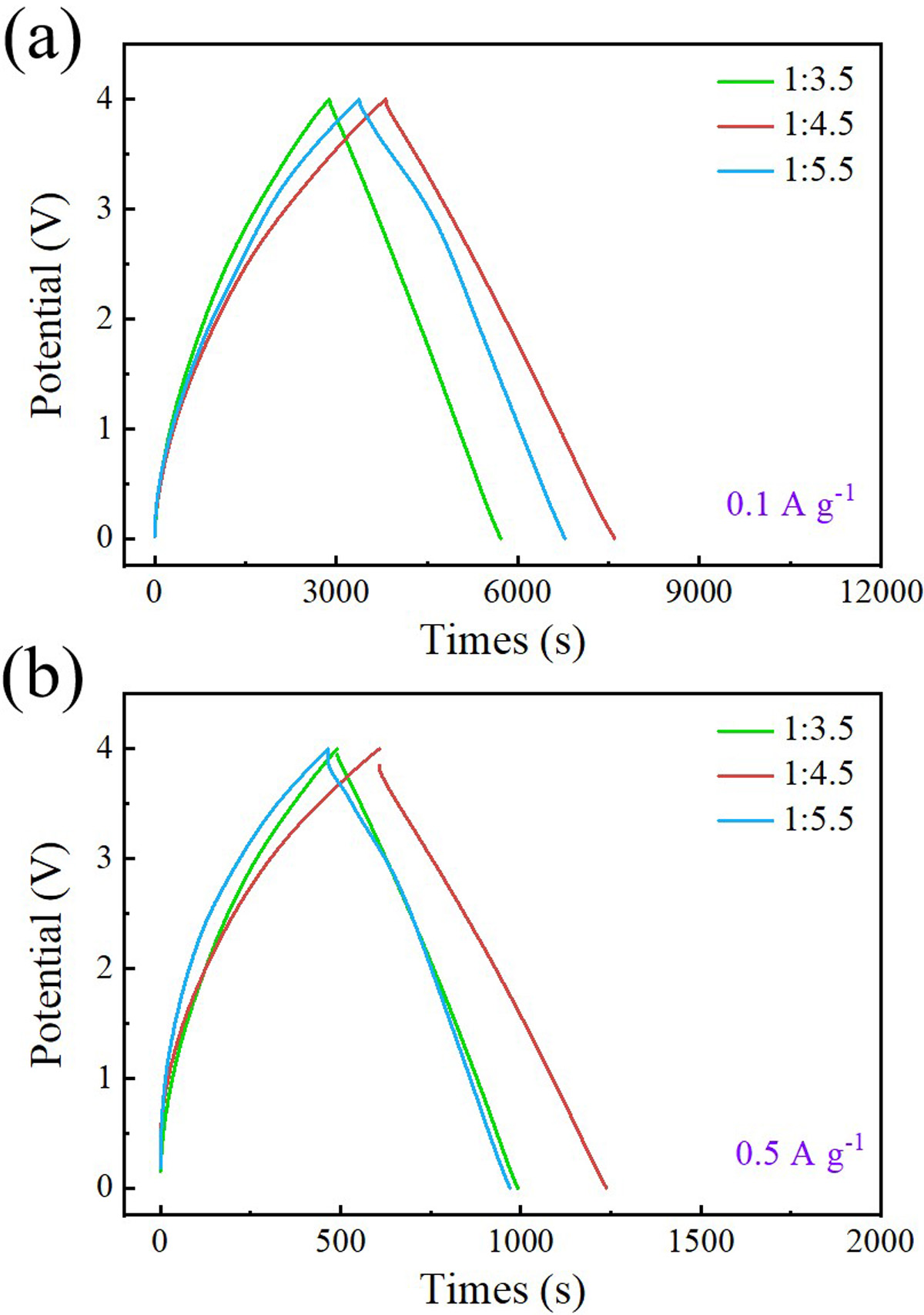


**Figure S43.** GCD curves of Nb/W-P@NPC//AC at (a) 0.1 and (b) 0.5 A g^-1^ with different mass ratios of anode to cathode.


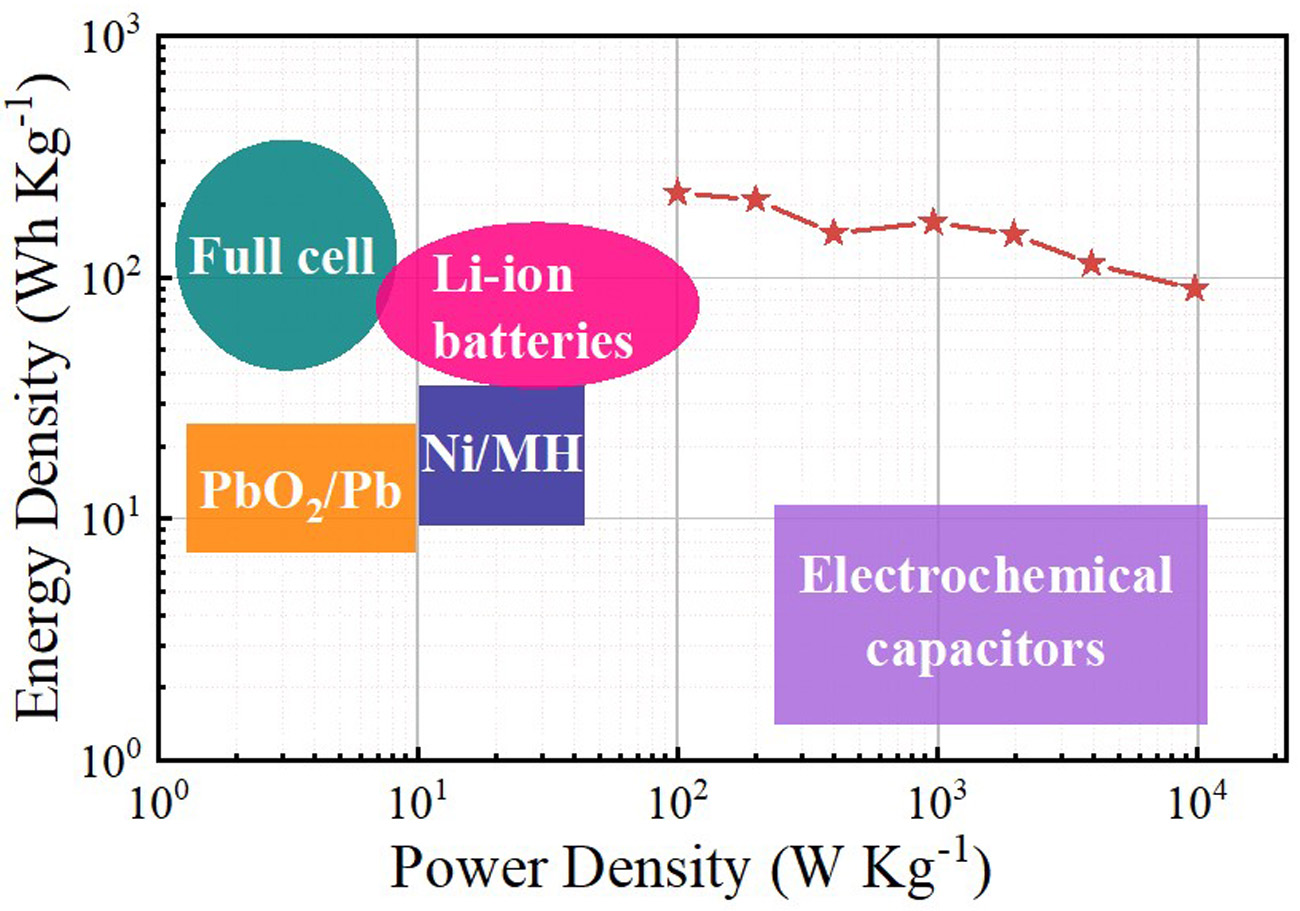


**Figure S44.** Ragone plot of Nb/W-P@NPC//AC *vs*. commercial energy storage devices.

**Table S1.** Atomic ratio of the components in Nb/W-P@NPC, W-P@NPC, W-P_L_@NPC and NP-C by XPS analyses.

| **Elements** | **Nb/W-P@NPC** **Atomic Ratio (%)** | **W-P@NPC Atomic Ratio (%)** | **W-P_L_@NPC Atomic Ratio (%)** | **NP-C**  **Atomic Ratio (%)** |
| --- | --- | --- | --- | --- |
| C 1*s* | 78.7 | 77.75 | 85.99 | 87.95 |
| N 1*s* | 7.49 | 6.54 | 6.15 | 5.92 |
| P 2*p* | 9.16 | 10.97 | 3.63 | 6.13 |
| W 4*f* | 3.43 | 4.47 | 4.23 |  |
| Nb 3*d* | 1.22 |  |  |  |

**Table S2.** Fitting parameters of W L-edge and Nb K-edge EXAFS for the different samples.

| **Sample** | **Shell** | **CN^a^** | **R(Å)^b^** | **σ^2^(Å^2^)^c^** | **ΔE_0_(eV)^d^** | **S_0_^2^** | **R factor** |
| --- | --- | --- | --- | --- | --- | --- | --- |
| W foil | W-W | 8 | 2.73 | 0.0027 | 5.1±1.3 | 0.84 | 0.0061 |
|  | W-W | 6 | 3.14 | 0.0026 |  |  |  |
| W-P@NPC | W-O | 1.0 | 1.77 | 0.0104 | 3.0±1.2 | 0.84 | 0.0129 |
|  | W-P | 4.5 | 2.26 | 0.0119 |  |  |  |
| Nb/W-P@NPC | W-O | 0.9 | 1.76 | 0.0049 | 3.4±1.0 | 0.84 | 0.0101 |
|  | W-P | 4.5 | 2.26 | 0.0116 |  |  |  |
|  | W-W/Nb | 8.6 | 3.81 | 0.0164 |  |  |  |
| Nb foil | Nb-Nb | 8 | 2.85 | 0.0063 | 4.9±0.9 | 0.92 | 0.0083 |
|  | Nb-Nb | 6 | 3.29 | 0.0067 |  |  |  |
| Nb/W-P@NPC | Nb-O | 0.3 | 1.73 | 0.0038 | -2.0±1.2 | 0.92 | 0.0133 |
|  | Nb-P | 5.6 | 2.50 | 0.0120 |  |  |  |
|  | Nb-W | 0.7 | 3.23 | 0.0203 |  |  |  |

*^a^CN*, coordination number; *^b^R*, the distance to the neighboring atom; *^c^σ*^2^, the Mean Square Relative Displacement (MSRD); *^d^ΔE*_0_, inner potential correction; *R* factor indicates the goodness of the fit. *S*0^2^ was fixed to 0.8032, according to the experimental EXAFS fit of the sample foil by fixing *CN* as the known crystallographic value. This value was fixed during EXAFS fitting, based on the known structure of W and Nb foils. Data ranges: 3.0 ≤ k ≤ 12.0 Å^-1^, 1.0 ≤ R ≤ 2.0 Å. The Debye-Waller factors and Δ*R*s are based on the *guessing* parameters and constrained for paths.

**Table S3.** Comparison of the cycling stability with previously reported relevant anodes.

| **Material description** | **Current**  **Density** | **Final**  **Capacity** | **Capacity Retention** | **Cycle**  **Number** | **Refs./Year** |
| --- | --- | --- | --- | --- | --- |
| DS-Cu_3_P | 0.05 A g^-1^ | 325 mAh g^-1^ | 87.7% | 100 cycles | ^[7]^2020 |
| FeP@NC | 10.0 A g^-1^ | 90.4 mAh g^-1^ | 82.0% | 10000 cycles | ^[8]^2021 |
| a-VO_x_/V_2_C | 2.0 A g^-1^ | 54 mAh g^-1^ | 56.3% | 1800 cycles | ^[9]^2021 |
| F-NiP_2_@carbon | 5.0 A g^-1^ | 155 mAh g^-1^ | 77.5% | 1000 cycles | ^[10]^2022 |
| Nb_2_O_5-x_@MEC | 20.0 A g^-1^ | 105 mAh g^-1^ | 100% | 5000 cycles | ^[11]^2022 |
| Co_x_P@CFC | 5.0 A g^-1^ | 230 mAh g^-1^ | 100% | 9000 cycles | ^[12]^2022 |
| CoP-C@MoS_2_/C | 5.0 A g^-1^ | 234 mAh g^-1^ | 90.2% | 1000 cycles | ^[13]^2023 |
| NbSSe/NC | 1.0 A g^-1^ | 347.8 mAh g^-1^ | 95.6% | 1000 cycles | ^[14]^2023 |
| W-N clusters/CNSs | 5.0 A g^-1^ | 255.6 mAh g^-1^ | 109.8% | 6000 cycles | ^[15]^2023 |
| ZnSe/CoSe_2_@NPC | 10.0 A g^-1^ | 133.9 mAh g^-1^ | 98.6% | 500 cycles | ^[16]^2023 |
| T-Nb_2_O_5-x_-NbSe_2_@C | 1.0 A g^-1^ | 95 mAh g^-1^ | 81.9% | 40000 cycles | ^[17]^2023 |
| Nb_2_O_5_@C-650 | 5 A g^-1^ | 90 mAh g^-1^ | 73.8% | 1000 cycles | ^[18]^2024 |
| Nb/W-P@NPC | 3 A g^-1^ | 316.7 mAh g^-1^ | 98.3% | 3000 cycles | **This Work** |
|  | 10 A g^-1^ | 190.6 mAh g^-1^ | 92.3% | 5000 cycles |  |

**Table S4.** Detailed elemental contents in the different regions (**Figure S37b**) by EDX analyses of Nb/W-P@NPC after 2000 cycles and discharged to 0.1 V.

|  | **Area #1** | | **Area #2** | |
| --- | --- | --- | --- | --- |
| **Elements** | **Atomic Ratio (%)** | **Mass Ratio (%)** | **Atomic Ratio (%)** | **Mass Ratio (%)** |
| C | 68.72 | 48.9 | 60.62 | 34.8 |
| N | 1.25 | 1.04 | 1.21 | 0.81 |
| Na | 10.18 | 13.87 | 1.88 | 13.05 |
| P | 2.03 | 3.73 | 2.92 | 4.32 |
| Nb | 0.68 | 3.74 | 1.43 | 6.35 |
| W | 1.26 | 13.67 | 2.98 | 26.17 |

**Table S5.** Comparison of cycling stability of Nb/W-P@NPC//AC with previously reported SIHCs.

| **Material description** | **Current**  **Density** | **Capacity**  **Loss Per Cycle** | **Cycle**  **Number** | **Refs./Year** |
| --- | --- | --- | --- | --- |
| HIC//AC | 0.4 A g^-1^ | 0.003% | 10000 cycles | ^[19]^2019 |
| N-Ti_3_C_2_T_x_//AC | 2.0 A g^-1^ | 0.0071% | 3500 cycles | ^[20]^2020 |
| MnO@HCNb//ACs | 1.0 A g^-1^ | 0.003% | 1000 cycles | ^[21]^2020 |
| FeVO//NVOPF | 1.2 A g^-1^ | 0.0036% | 9000 cycles | ^[22]^2021 |
| ATS/GNS//NS-PC | 5.0 A g^-1^ | 0.003% | 10000 cycles | ^[23]^2021 |
| 3DP-ZnV_2_O_4_NFs@N-PC//AC | 1.0 A g^-1^ | 0.003% | 8000 cycles | ^[24]^2022 |
| FS/TO@C//AC | 1.0 A g^-1^ | 0.0257% | 1000 cycles | ^[25]^2022 |
| CAC1300//AC900 | 1.0 A g^-1^ | 0.0064% | 2000 cycles | ^[26]^2022 |
| Zn-N_3_S-NSC//AC | 1.0 A g^-1^ | 0.005% | 1000 cycles | ^[27]^2023 |
| TH-HCS//AC | 1.0 A g^-1^ | 0.0038% | 5000 cycles | ^[28]^2023 |
| Co-MOF-74\|FGO-180//AC | 1.0 A g^-1^ | 0.0045% | 2000 cycles | ^[29]^2023 |
| CoS-C//AC | 2.0 A g^-1^ | 0.029% | 1000 cycles | ^[30]^2023 |
| NPC@MoS_2_/MXene//AC | 2.0 A g^-1^ | 0.0115% | 1000 cycles | ^[31]^2024 |
| MoSeTe/N, F@C//AC | 1.0 A g^-1^ | 0.0214% | 3500 cycles | ^[32]^2024 |
| Nb/W-P@NPC//AC | 1.0 A g^-1^ | 0.0034% | 8000 cycles | **This Work** |

**Equations S4 and S5** ^[33, 34]^**:**

$Z^{'}=R_{s}+R_{ct}+\sigma\omega^{-\frac{1}{2}}$ (S4)

$D_{{Na}^{+}}=\frac{R^{2}T^{2}}{2A^{2}n^{2}F^{4}C^{2}\sigma^{2}}$ (S5)

where *R*_ct_ is the charge-transfer resistance, *σ* is the slope, ω is the angular frequency, *D*_Na_^+^ is the ion diffusion coefficients, the *R* is the gas constant, *T* is the absolute temperature, *A* is the contact area of electrode, *n* is the transferred charge, *F* is the Faraday constant and *C* is the concentration of Na^+^.

**Equations S6** ^[35]^**:**

$D_{{Na}^{+}}=\frac{4}{\pi}{\left( \frac{V_{M}}{AFZ_{i}} \right)^{2}\left[ \frac{I_{0}\left( \frac{dE}{d\delta} \right)}{\left( \frac{dE}{d\sqrt{t}} \right)} \right]}^{2}, t\ll\frac{L^{2}}{D_{{Na}^{+}}}$ (S6)

where *D*_Na_^+^ is the ion diffusion coefficients, *V*_M_ is the molar volume, *A* is the contact area of electrode, *F* is the Faraday constant, *Z_i_* is the valence of substance, *I*_0_ is the applied constant current, *E* is the electrode voltage, *δ* is the stoichiometric ratio, *t* is the time during constant current pulse and *L* is the thickness of the electrode.

**References:**

[1] G. Kresse, J. Furthmüller, *Phys Rev B* **1996**, *54*, 11169.

[2] J. P. Perdew, K. Burke, M. Ernzerhof, *Phys Rev Lett* **1996**, *77*, 3865.

[3] G. Kresse, D. Joubert, *Phys Rev B* **1999**, *59*, 1758.

[4] P. E. Blöchl, *Phys Rev B* **1994**, *50*, 17953.

[5] S. Grimme, J. Antony, S. Ehrlich, H. Krieg, *J Chem Phys* **2010**, *132*, 154104.

[6] G. Henkelman, B. P. Uberuaga, H. Jónsson, *J Chem Phys* **2000**, *113*, 9901.

[7] Z. Hu, Q. Liu, W. Lai, Q. Gu, L. Li, M. Chen, W. Wang, S. Chou, Y. Liu, S. Dou, *Adv Energy Mater* **2020**, *10*, 1903542.

[8] C. Wang, J. Yan, T. Li, Z. Lv, X. Hou, Y. Tang, H. Zhang, Q. Zheng, X. Li, *Angew Chem Int Edit* **2021**, *60*, 25013.

[9] W. Zhang, J. Peng, W. Hua, Y. Liu, J. Wang, Y. Liang, W. Lai, Y. Jiang, Y. Huang, W. Zhang, H. Yang, Y. Yang, L. Li, Z. Liu, L. Wang, S. Chou, *Adv Energy Mater* **2021**, *11*, 2100757.

[10] L. Wu, L. Wang, X. Cheng, M. Ma, Y. Wu, X. Wu, H. Yang, Y. Yu, C. He, *Nano Res* **2022**, *15*, 2147.

[11] D. Luo, C. Ma, J. Hou, Z. Zhang, R. Feng, L. Yang, X. Zhang, H. Lu, J. Liu, Y. Li, Y. Zhang, X. Wang, Z. Chen, *Adv Energy Mater* **2022**, *12*, 2103716.

[12] G. Yuan, D. Liu, X. Feng, M. Shao, Z. Hao, T. Sun, H. Yu, H. Ge, X. Zuo, Y. Zhang, *Adv Mater* **2022**, *34*, 2108985.

[13] Y. Xia, T. Yang, Z. Wang, T. Mao, Z. Hong, J. Han, D. Peng, G. Yue, *Adv Funct Mater* **2023**, *33*, 2302830.

[14] Y. Liu, M. Qiu, X. Hu, J. Yuan, W. Liao, L. Sheng, Y. Chen, Y. Wu, H. Zhan, Z. Wen, *Nano-Micro Lett* **2023**, *15*, 104.

[15] S. Chu, M. Yu, Y. Pan, S. Hu, B. Liu, T. Lu, F. Zeng, S. Luo, *Small* **2023**, *19*, 2300619.

[16] Z. Cao, J. Cui, D. Yu, Y. Wang, J. Liu, J. Zhang, J. Yan, Y. Zhang, S. Sun, Y. Wu, *Adv Funct Mater* **2023**, *33*, 2306862.

[17] C. Liu, B. Wang, Z. Song, X. Xiao, Z. Cao, D. Xiong, W. Deng, H. Hou, Y. Yang, G. Zou, X. Ji, *Adv Funct Mater* **2024**, *34*, 2312905.

[18] J. Ma, J. Qin, S. Zheng, Y. Fu, L. Chi, Y. Li, C. Dong, B. Li, F. Xing, H. Shi, Z. S. Wu, *Nano-Micro Lett* **2024**, *16*, 67.

[19] Z. Xu, M. Wu, Z. Chen, C. Chen, J. Yang, T. Feng, E. Paek, D. Mitlin, *Adv Sci* **2019**, *6*, 1802272.

[20] Z. Fan, C. Wei, L. Yu, Z. Xia, J. Cai, Z. Tian, G. Zou, S. X. Dou, J. Sun, *ACS Nano* **2020**, *14*, 867.

[21] J. Qin, H. M. K. Sari, X. Wang, H. Yang, J. Zhang, X. Li, *Nano Energy* **2020**, *71*, 104594.

[22] Q. Wei, Q. Li, Y. Jiang, Y. Zhao, S. Tan, J. Dong, L. Mai, D. Peng, *Nano-Micro Lett* **2021**, *13*, 55.

[23] Z. Sun, K. Zhu, P. Liu, Y. Si, H. Li, L. Jiao, *Energy Storage Mater* **2021**, *41*, 32.

[24] J. Yuan, M. Qiu, J. X. Chen, X. Hu, Y. Liu, B. Yu, G. Zhong, Z. Weng, H. Zhan, Z. Wen, *Adv Funct Mater* **2022**, *32*, 2203732.

[25] X. Xiao, X. Duan, Z. Song, X. Deng, W. Deng, H. Hou, R. Zheng, G. Zou, X. Ji, *Adv Funct Mater* **2022**, *32*, 2110476.

[26] K. Wang, F. Sun, H. Wang, D. Wu, Y. Chao, J. Gao, G. Zhao, *Adv Funct Mater* **2022**, *32*, 2203725.

[27] L. Liu, Z. Du, J. Sun, S. He, K. Wang, M. Li, L. Xie, W. Ai, *Small* **2023**, *19*, 2300556.

[28] J. Ruan, S. Luo, S. Wang, J. Hu, F. Fang, F. Wang, M. Chen, S. Zheng, D. Sun, Y. Song, *Adv Energy Mater* **2023**.

[29] Y. Fan, C. Li, X. Liu, J. Ren, Y. Zhang, J. Chi, L. Wang, *Chem Eng J* **2023**, *452*, 139585.

[30] Y. Yang, Y. Ma, X. Wang, Z. Gao, J. Yu, T. Liu, *Chem Eng J* **2023**, *455*, 140610.

[31] H. Pan, Y. Huang, X. Cen, M. Zhang, J. Hou, C. Wu, Y. Dou, B. Sun, Y. Wang, B. Zhang, L. Zhang, *Adv Sci* **2024**, *11*, 2400364.

[32] D. Yang, W. Guo, F. Guo, J. Zhu, G. Wang, H. Wang, G. Yuan, S. Ma, B. Wang, *J Energy Chem* **2024**, *90*, 652.

[33] N. Takami, A. Satoh, M. Hara, T. Ohsaki, *J Electrochem Soc* **1995**, *142*, 371.

[34] C. Ho, I. D. Raistrick, R. A. Huggins, *J Electrochem Soc* **1980**, *127*, 343.

[35] C. J. Wen, B. A. Boukamp, R. A. Huggins, W. Weppner, *J Electrochem Soc* **1979**, *126*, 2258.
